# Supplementary material for: Microtubules are not required to generate a nascent axon in embryonic spinal neurons in vivo
Source: EMBO Rep. 2022 Oct 4;23(11):e52493. doi: 10.15252/embr.202152493 (PMC9638849; doi:10.15252/embr.202152493)
Supplement: Supplementary file 1 — Appendix S1 [file EMBR-23-e52493-s006.pdf]

APPENDIX

Table of Contents:

Appendix Figure S1. Cell labelling randomly targets all early embryonic neuronal subtypes.
Appendix Figure S2. Ɣ-tubulin accumulates at one concentrated point during axon initiation.
Appendix Figure S3. Lifeact-Ruby is enriched in the nascent axon before nascent axon establishment.
Appendix Figure S4. EB3-GFP is not enriched in the nascent axon during nascent axon establishment.
Appendix Figure S5. Lifeact-Ruby is enriched in the nascent axon before EB3-GFP.
Appendix Figure S6. Kif5c560-YFP is enriched in the nascent axon following nascent axon establishment.
Appendix Figure S7. Lifeact-Ruby is enriched in the nascent axon before kif5c650-YFP.
Appendix Figure S8. Lifeact-Ruby is enriched in the nascent axon before EB3-GFP or kif5c560-YFP
Appendix Figure S9. Nocodazole treatment completely disrupts the microtubule array in newborn neurons and neuroepithelial cells.
Appendix Figure S10. LamMO-injected embryo have a similar phenotype to *Sly*$^{-/-}$ embryos.

A

RB	29
DoLA	9
CoPA	5
CoSA	28
CiA	19
CiD	19
CoB	14
VeLD	4
M	1
UCoD	24

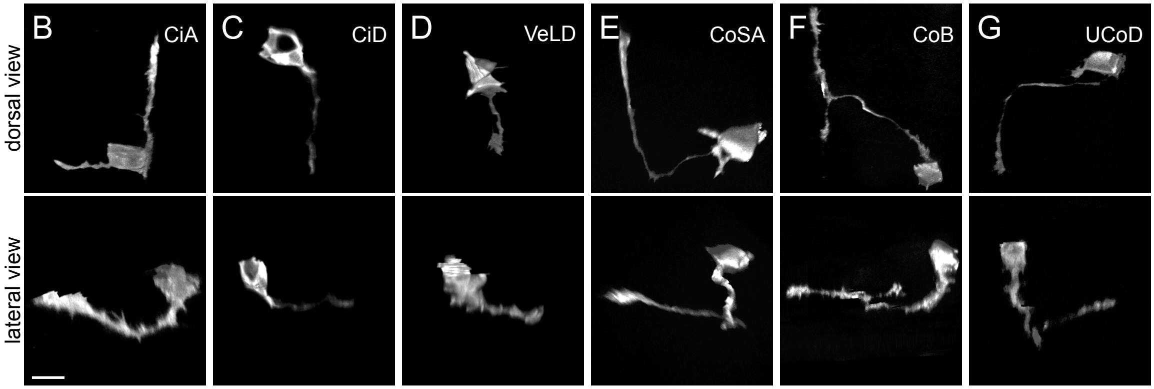

Appendix Figure S1 - Cell labelling randomly targets all early embryonic neuronal subtypes.

A Number of each neuronal subtype labelled.

B-G Examples of CiA (B), CiD (C), VeLD (D), CoSA (E), CoB (F) and CoD (G) neurons and their axon trajectories as dorsal and lateral reconstructions from confocal z-stacks. Scale bar = 10 µm.

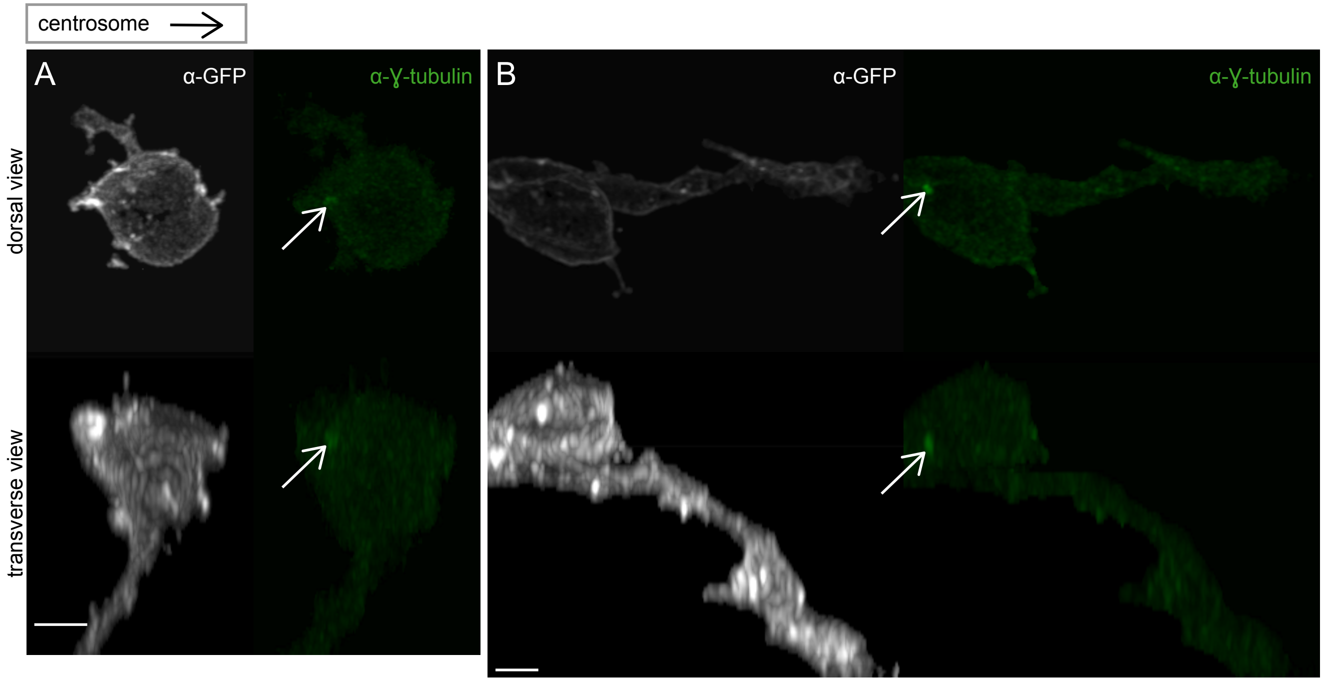

Appendix Figure S2 - γ-tubulin accumulates at one concentrated point during axon initiation.

A, B Two examples of neurons labelled with membrane label EGFP-CAAX in embryos processed for immunohistochemistry against GFP and γ-tubulin. γ-tubulin accumulations appear to correspond with the centrosome. Dorsal views are maximum projections from confocal z-stacks. Transverse views are 3D reconstructions from confocal z-stacks. Scale bars = 5 μm.

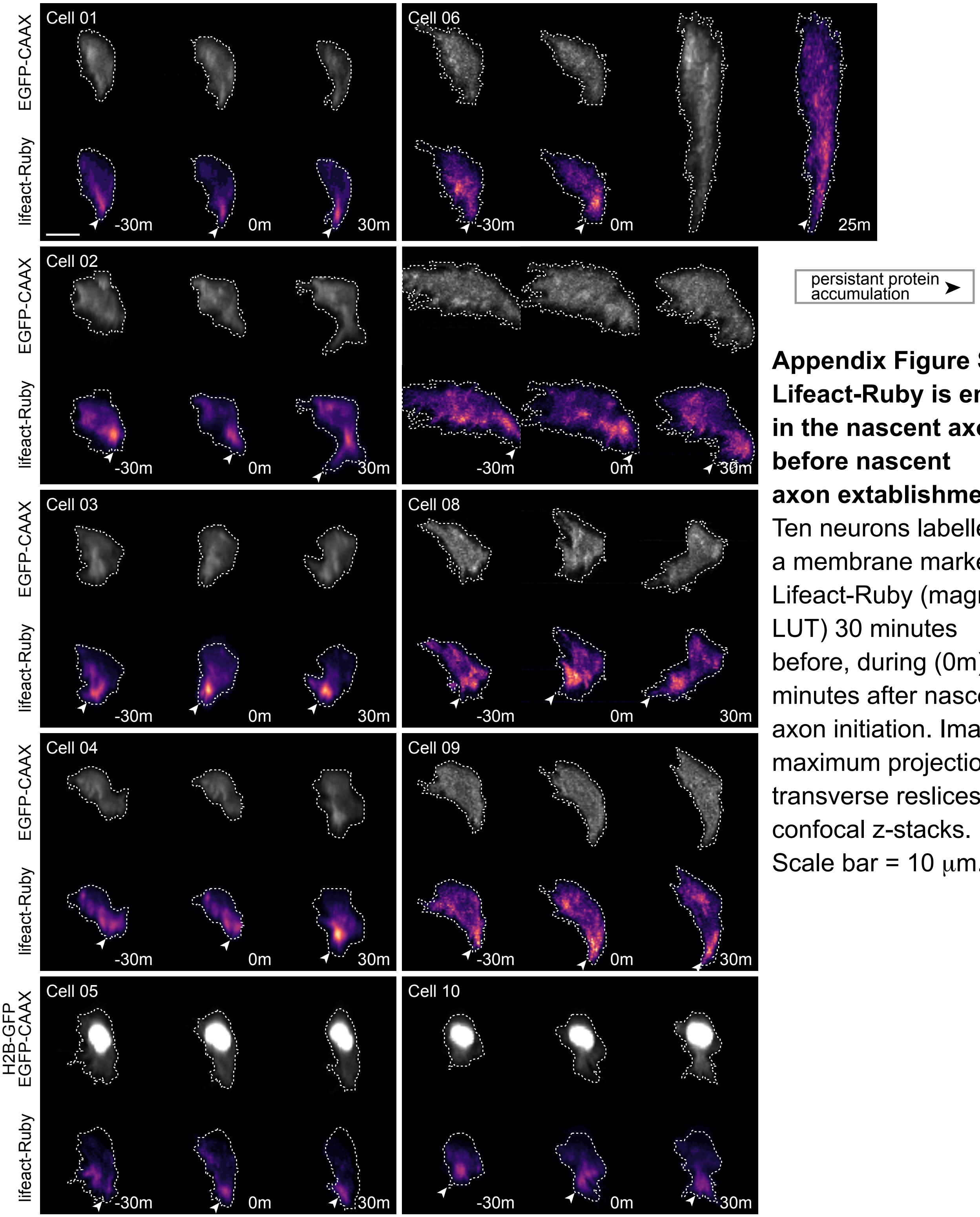

Appendix Figure S3 - Lifeact-Ruby is enriched in the nascent axon before nascent axon extablishment. Ten neurons labelled with a membrane marker and Lifeact-Ruby (magma LUT) 30 minutes before, during (0m) and 30 minutes after nascent axon initiation. Images are maximum projections of transverse reslices of confocal z-stacks. Scale bar = 10 μm.

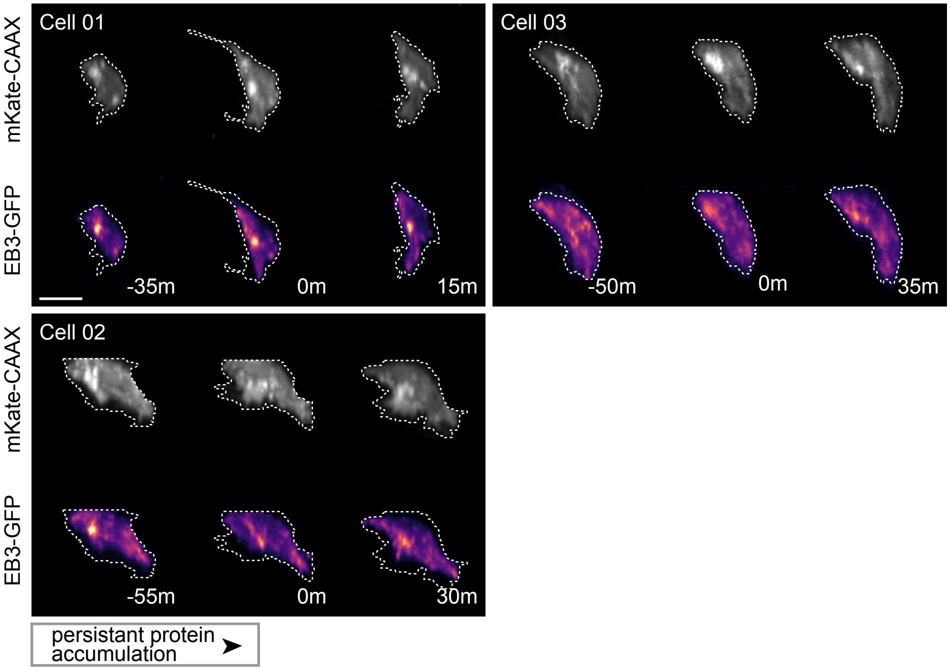

Appendix Figure S4 - EB3-GFP is not enriched in the nascent axon during nascent axon establishment.
Three neurons labelled with a membrane marker and EB3-GFP (magma LUT) 30 minutes before, during (0m) and 30 minutes after nascent axon initiation. Images are maximum projections of transverse reslices of confocal z-stacks. Scale bar = 10 μm.

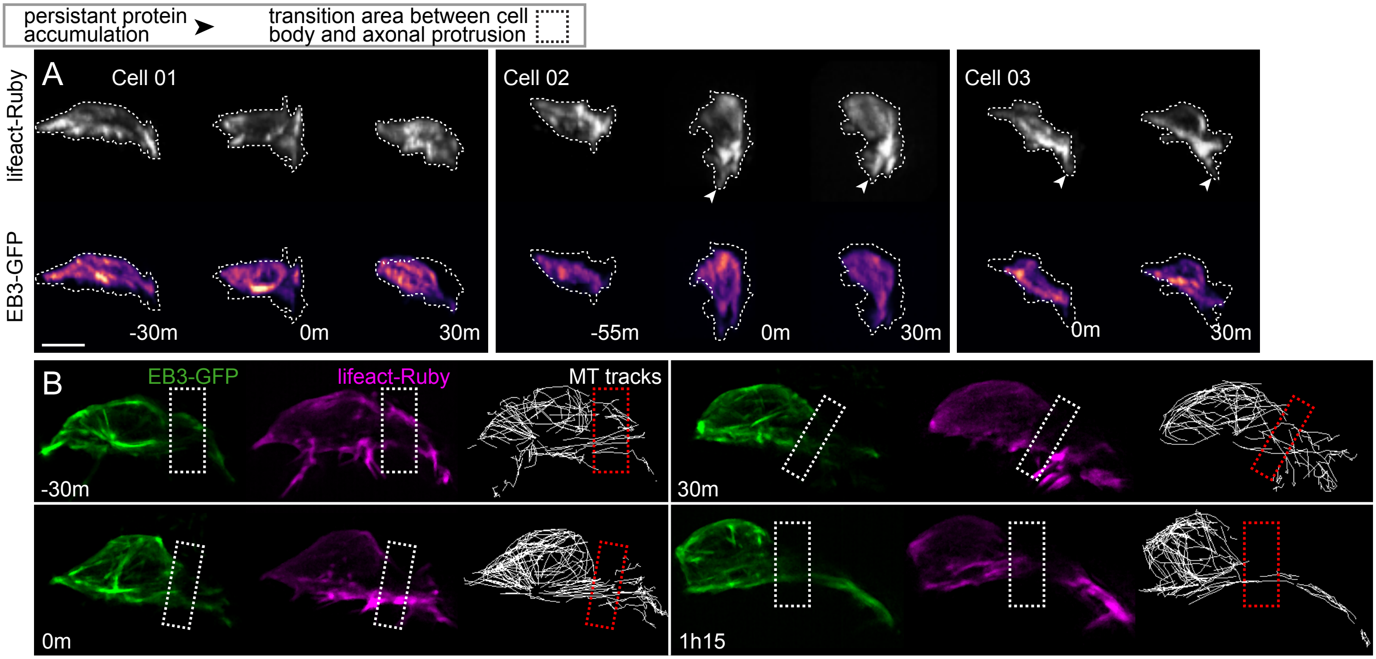

Appendix Figure S5 - Lifeact-Ruby is enriched in the nascent axon before EB3-GFP.

A Three neurons labelled with lifeact-Ruby and EB3-GFP (magma LUT) 30 minutes before, during (0m) and 30 minutes after nascent axon initiation. Images are maximum projections of transverse reslices of confocal z-stacks. Scale bar = 10 μm.

B Maximum projections of neuron in Figure 4C showing EB3-GFP, lifeact-Ruby and microtubule tracks before, during (0m) and after axon initiation.

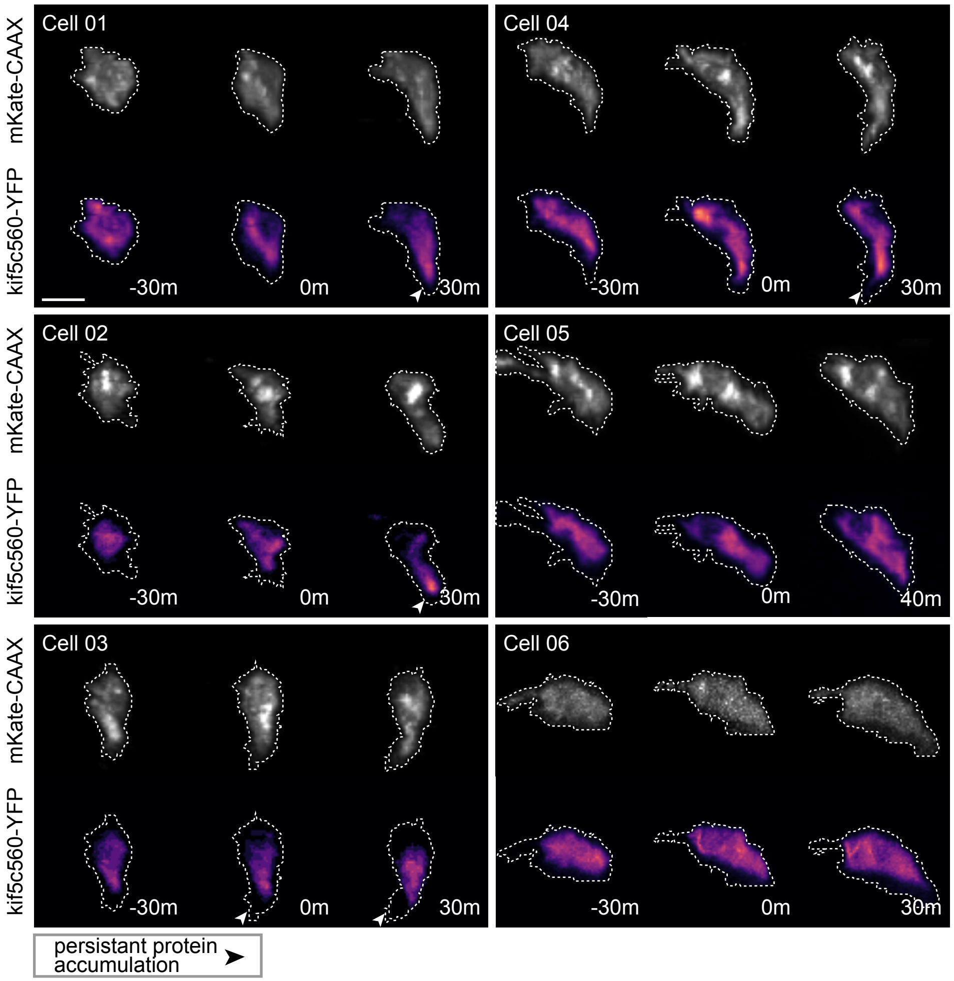

Appendix Figure S6 - Kif5c560-YFP is enriched in the nascent axon following nascent axon extablishment.

Six neurons labelled with a membrane marker and kif5c560-YFP (magma LUT) 30 minutes before, during (0m) and 30 minutes after nascent axon initiation. Images are maximum projections of transverse reslices of confocal z-stacks. Scale bar = 10 μm.

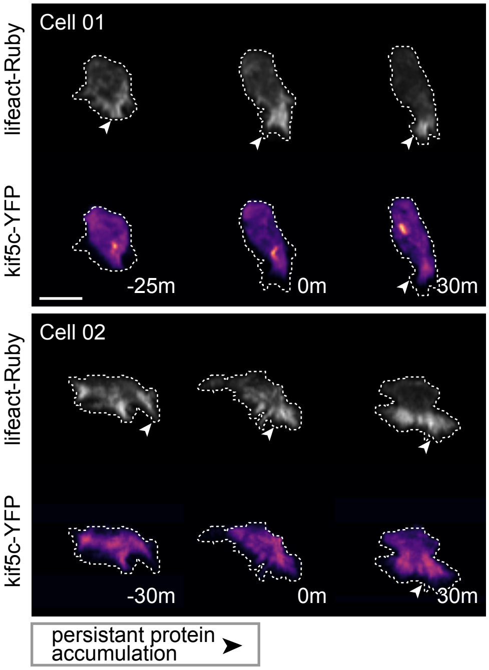

Appendix Figure S7 - Lifeact-Ruby is enriched in the nascent axon before kif5c560-YFP. Two neurons labelled with lifeact-Ruby and kif5c560-YFP (magma LUT) 30 minutes before, during (0m) and 30 minutes after nascent axon initiation. Images are maximum projections of transverse reslices of confocal z-stacks. Scale bar = 10 μm.

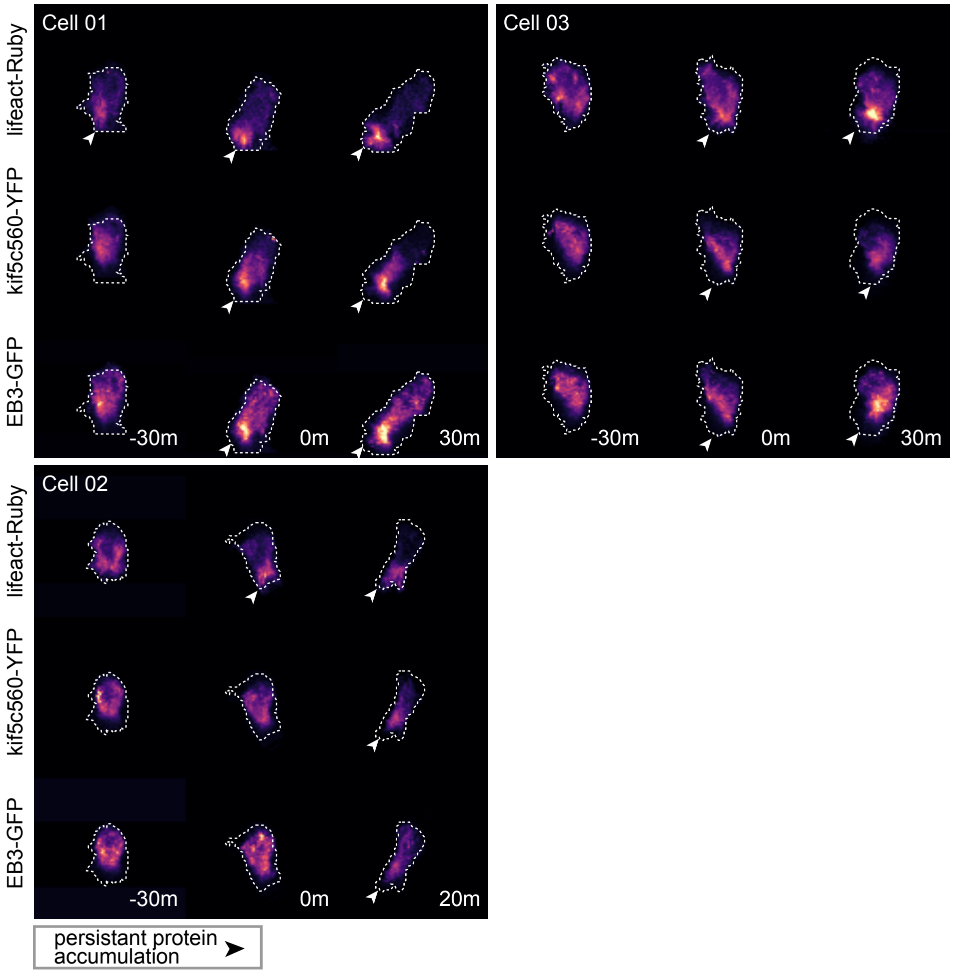

Appendix Figure S8 - Lifeact-Ruby is enriched in the nascent axon before EB3-GFP or kif5c560-YFP.

Four neurons labelled with lifeact-Ruby, kif5c560-YFP and EB3-GFP (all magma LUT) 30 minutes before, during (0m) and 30 minutes after nascent axon initiation. Images are maximum projections of transverse reslices of confocal z-stacks. Scale bar = 10 μm.

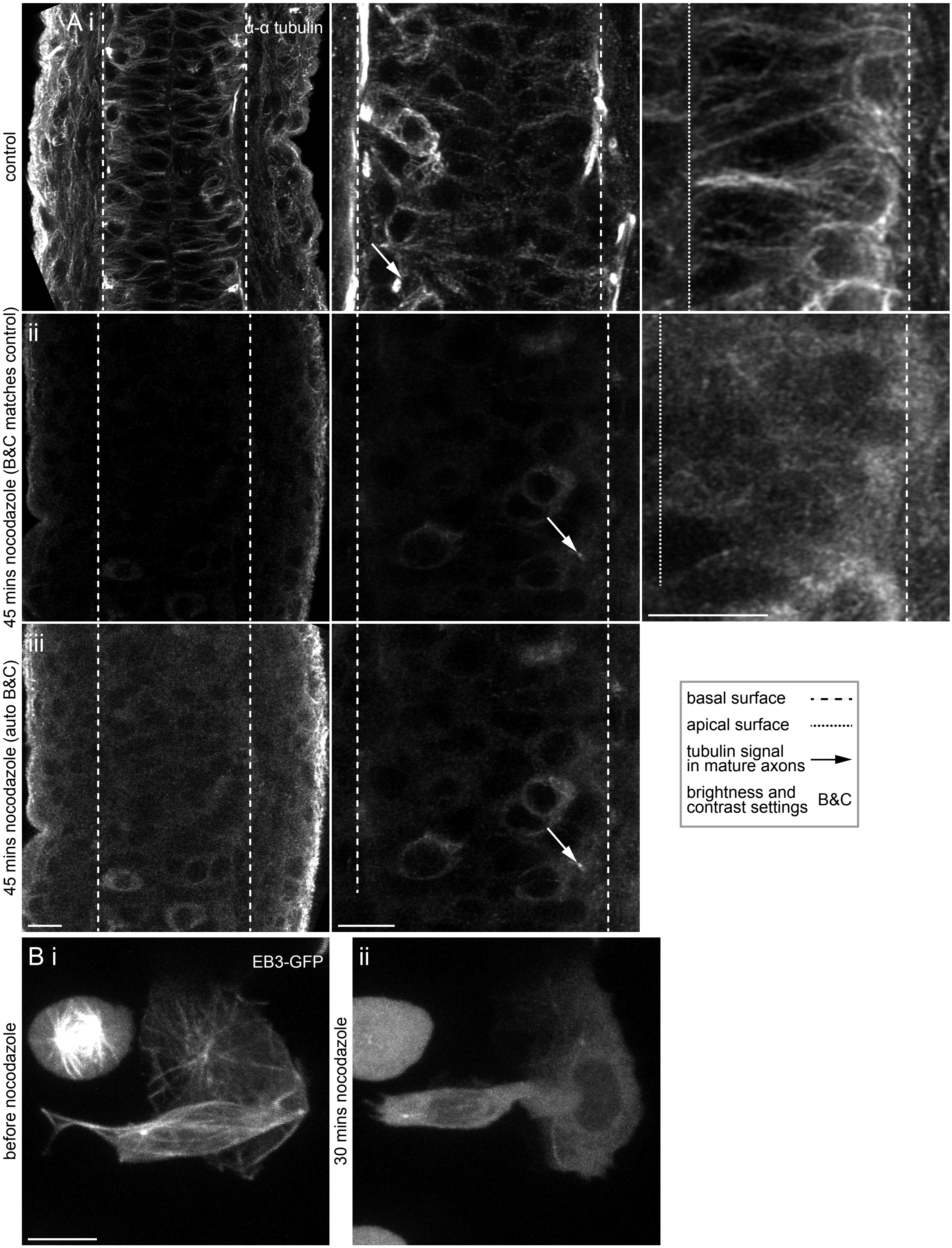
A i
α-α tubulin
control
ii
45 mins nocodazole (B&C matches control)
iii
45 mins nocodazole (auto B&C)
basal surface
apical surface
tubulin signal in mature axons
brightness and contrast settings
B&C
B i
EB3-GFP
before nocodazole
ii
30 mins nocodazole

Appendix Figure S9 - Nocodazole treatment completely disrupts the microtubule array in newborn neurons and neuroepithelial cells.

A Maximum projections of shallow z-stacks showing zebrafish embryos incubated in (i) fish water (control), or (ii, iii) in 5 μg/mL nocodazole for 45 minutes and processed for immunohistochemistry against α-tubulin. Three panels showing three different embryos imaged at different magnifications are shown for each condition. (ii) and (iii) show the same image but with brightness and contrast adjested to be the same as control (ii) or automatically adjusted (iii). Control images show clear microtubule arrays in all cells. After 45 minutes of nocodazole treatment the microtubule array is completely disrupted in neuroepithelial cells and newborn neurons, although remnants can be seen in mature neurons and their axons.

B Maximum projections of cells labelled with EB3-GFP before and after nocodazole treatment. EB3 comets labelling microtubule plus-ends present before nocodazole treatment are not visible within 30 minutes of nocodazole treatment.

Scale bars = 10 μm.

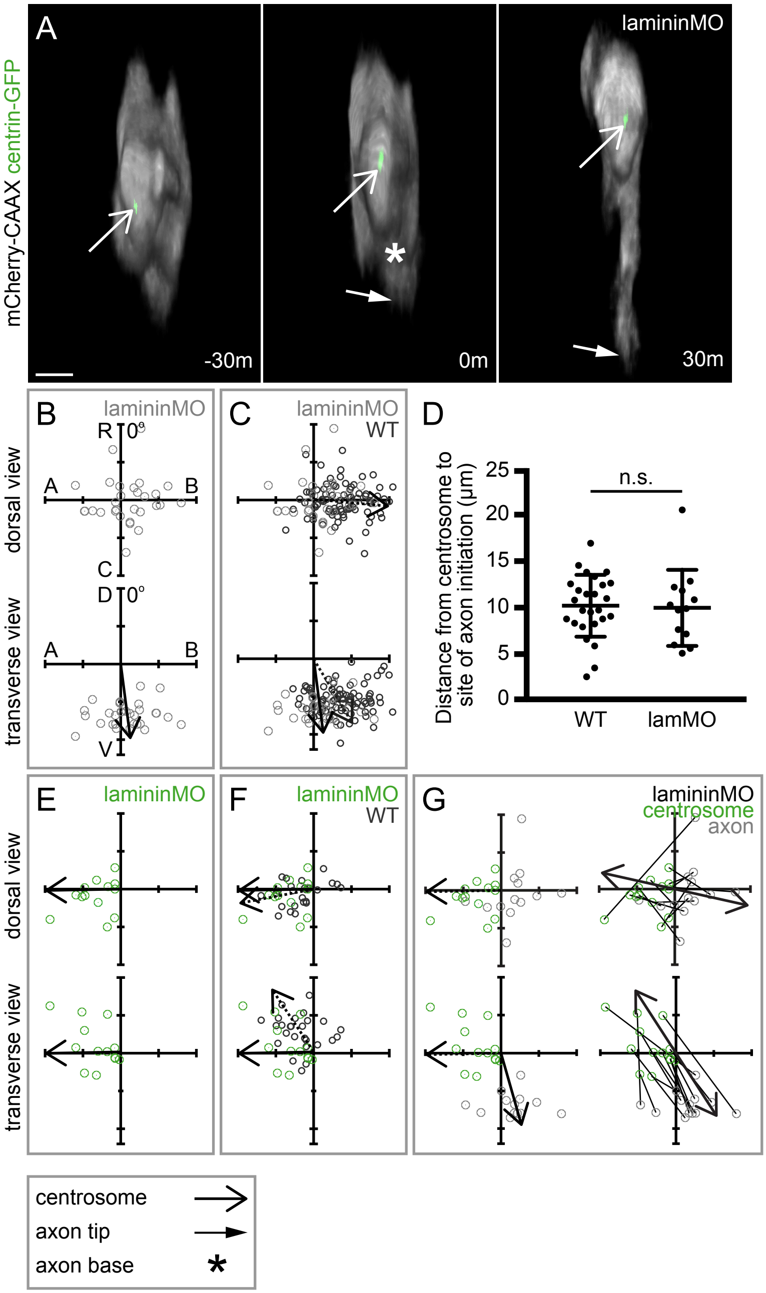

A
mCherry-CAAX centrin-GFP
lamininMO
-30m
0m
30m
B
laminin MO
dorsal view
transverse view
R
0°
A
B
C
D
V
C
lamininMO
WT
D
Distance from centrosome to site of axon initiation (µm)
n.s.
25
20
15
10
5
0
WT
lamMO
E
lamininMO
F
lamininMO
WT
G
lamininMO
centrosome
axon
centrosome
axon tip
axon base

Appendix Figure S10 - LamMO-injected embryos have similar phenotype to *Sly*$^{-/-}$ embryos.

A Image sequence from confocal time lapse shows a neuron in a lamMO-injected embryo labelled with membrane and centrosome markers before (-30m), during (0m) and after (30m) axon initiation. Images are transverse reconstructions from confocal z-stacks.

B Plots showing axon position on the soma relative to the cell centroid at 0,0 for dorsal and transverse view in lamMO-injected embryos (n = 29 cells from three experiments). Axon position is random in dorsal view (0.1 < *P* < 0.5) but not in transverse view (*P* < 0.001, mean = 172.6^{o}; Moore's modification of Rayleigh's test).

C Plots showing merge of WT and lamMO axon positions on the cell body relative to the cell centroid at 0,0 for dorsal and transverse views. WT: n = 86 cells from 8 experiments; lamMO: n = 29 cells from three experiments. Axon positions in WT and lamMO-injected embryos are significantly different (dorsal view 0.01 < *P* < 0.02, transverse view *P* < 0.001; Batschelet's alternative to Hotelling test).

D Graph showing distance between centrosome and base of axon at time of axon initiation in WT and lamMO-injected embryos. Bars show mean and standard deviation. WT: n = 26 cells from three experiments, mean = 10.13 μm, s.d. 3.35; lamMO: n = 13 cells from three experiments, mean = 9.9 μm, s.d. = 4.11. One-way ANOVA, *P* = 0.980.

E Plots showing centrosome position relative to the cell centroid at 0,0 for dorsal and transverse view in lamMO-injected embryos (n = 13 cells from three experiments). Centrosome position is not random (dorsal view *P* < 0.001, mean = -91.0^{o}; transverse view *P* < 0.001, mean = -91.1^{o}; Moore's modification of Rayleigh's test).

F Plots showing merge of WT and lamMO centrosome positions on the cell body relative to the cell centroid at 0,0 for dorsal and transverse views. WT: n = 26 cells from three experiments. lamMO: n = 13 cells from three experiments. Centrosome positions are not significantly different between WT and lamMO-injected embryos in dorsal view (0.2 < *P* < 0.5) but are in transverse view (0.02 < *P* < 0.05; Batschelet's alternative to Hotelling test).

G Plots showing positions of the centrosome and base of axon in lamMO-injected embryos at the time of axon initiation relative to the cell centroid at 0,0 for dorsal and transverse view (n = 13 cells from three experiments). Left-hand plots: centrosome position is not random (dorsal view *P* < 0.001; mean = -91.0^{o}; transverse view *P* < 0.001, mean = -91.1^{o}) and axon position is random in dorsal view (0.05 < *P* < 0.1) but not in transverse view (*P* < 0.001, mean = 164.1^{o}; Moore's modification of Rayleigh's test). Centrosome and axon positions are significantly different (dorsal view *P* < 0.001, transverse view *P* < 0.001; Moore's test for paired data). Right-hand plots: vectors linking centrosome and nascent axon from the same cell are not random (dorsal view *P* < 0.001, mean = 102.6^{o}; transverse view *P* < 0.001, mean = 147.1^{o}).

Scale bars = 10 μm.
