# Supplementary material for: Microtubules are not required to generate a nascent axon in embryonic spinal neurons in vivo
Source: EMBO Rep. 2022 Oct 4;23(11):e52493. doi: 10.15252/embr.202152493 (PMC9638849; doi:10.15252/embr.202152493)
Supplement: Supplementary file 2 — Expanded View Figures PDF [file EMBR-23-e52493-s001.pdf]

Expanded View Figures

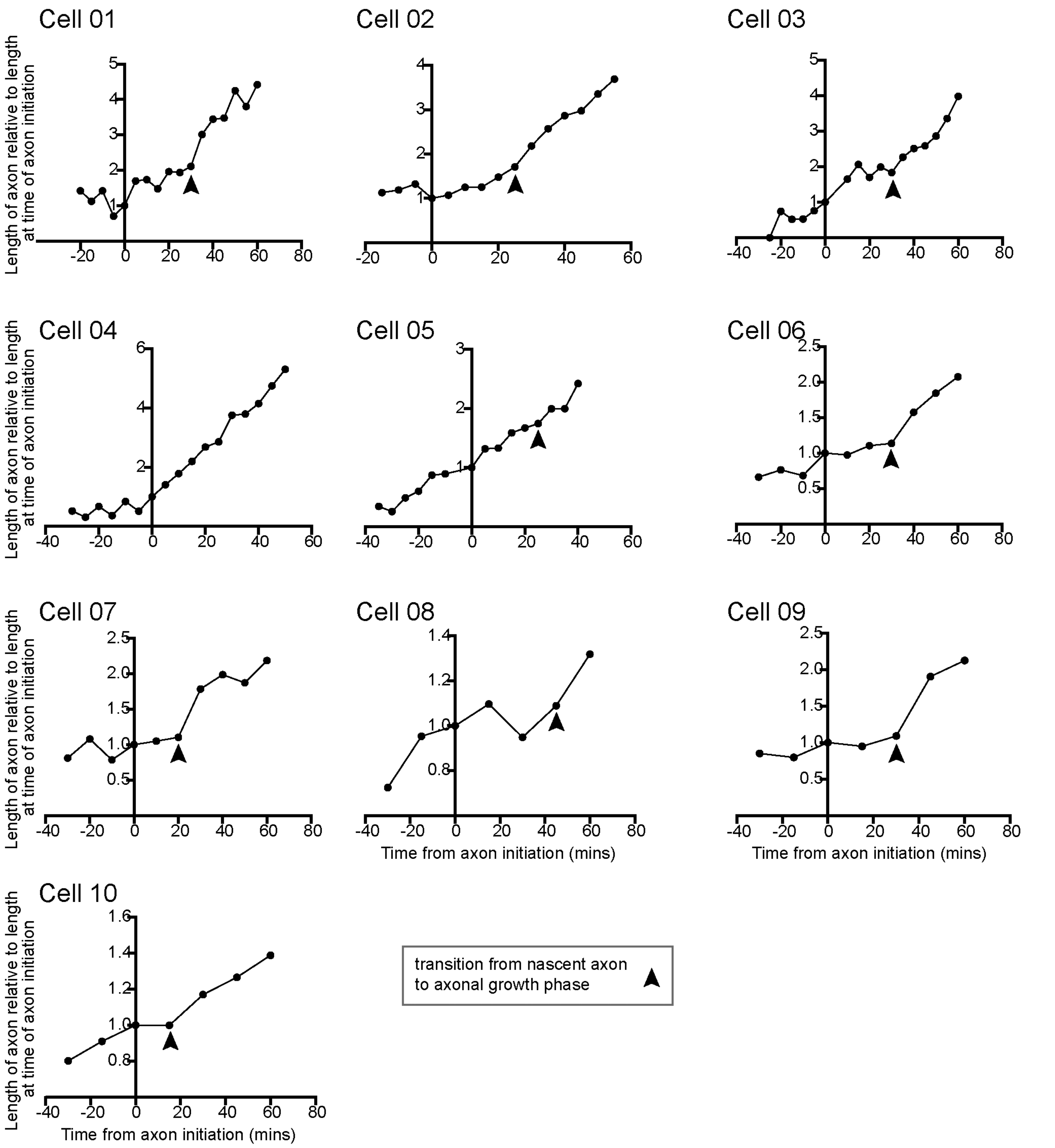

Figure EV1. Axon initiation often has two distinct phases.

Graphs showing the maximum protrusion length from ten cells from four experiments before, during (0 min) and after axon initiation. Length is shown relative to length at the time of axon initiation (0 min). Axon initiation identified by the persistent length and position of a dilated protrusion that later transitions to growing axon. Arrowheads show transition from nascent axon to growth phase.

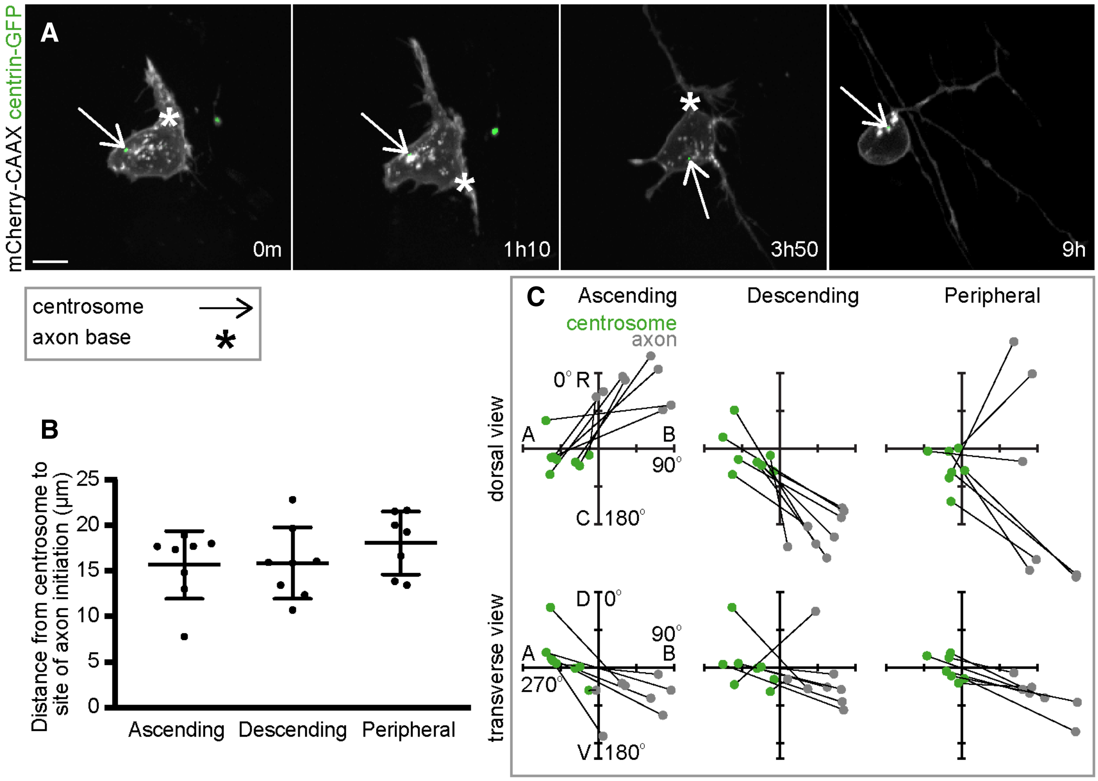

Figure EV2. The centrosome is not close to Rohon-Beard neuron axons during initiation.

A Image sequence from confocal time lapse shows a Rohon-Beard neuron labelled with membrane and centrosome markers during the initiation of the ascending (0 m), descending (1 h10 min) and peripheral axons (3 h 50 min), and during axon pathfinding. The centrosome is located away from the base of each axon but moves close to the peripheral axon during pathfinding. Images are maximum projections from confocal z-stacks. Scale bar = 10 µm.

B Graph showing distance between centrosome and base of the axon at time of initiation of each axon ($n = 23$ events from 8 cells from three experiments). Bars show mean and standard deviation.

C Plots showing the positions of the centrosome and base of the axon at the time of axon initiation relative to the cell centroid at 0,0 for dorsal and transverse views for ascending ($n = 8$ cells), descending ($n = 8$ cells) and peripheral axons ($n = 7$ cells). Lines connect centrosome and nascent axon from the same cell.

 © 2022 The Authors
