# Supplementary material for: Microtubules are not required to generate a nascent axon in embryonic spinal neurons in vivo
Source: EMBO Rep. 2022 Oct 4;23(11):e52493. doi: 10.15252/embr.202152493 (PMC9638849; doi:10.15252/embr.202152493)
Supplement: Supplementary file 3 — Movie EV1 [file EMBR-23-e52493-s018.zip › Movie EV1/Movie EV1.docx]

**Movie EV1 -Early steps of differentiation in the zebrafish spinal cord.** Maximum projection of confocal timelapse, dorsal view. A neuron labelled with a membrane marker extends two transient protrusions along the basal surface (open arrows). They are retracted, along with the apical attachment (arrowhead), before the axon is extended (closed arrow).
