# Supplementary material for: Microtubules are not required to generate a nascent axon in embryonic spinal neurons in vivo
Source: EMBO Rep. 2022 Oct 4;23(11):e52493. doi: 10.15252/embr.202152493 (PMC9638849; doi:10.15252/embr.202152493)
Supplement: Supplementary file 4 — Movie EV2 [file EMBR-23-e52493-s021.zip › Movie EV2/Movie EV2.docx]

**Movie EV2 - Axon initiation is highly stereotyped.** Transverse reconstruction of confocal time lapse. The neuron, labelled with a membrane marker, first extends multiple transient pre-axonal protrusions (arrowheads). It then extends a nascent axon (0 mins), which is maintained for approximately 30 minutes before axon growth begins. Arrows show axon tip.
