# Supplementary material for: Microtubules are not required to generate a nascent axon in embryonic spinal neurons in vivo
Source: EMBO Rep. 2022 Oct 4;23(11):e52493. doi: 10.15252/embr.202152493 (PMC9638849; doi:10.15252/embr.202152493)
Supplement: Supplementary file 5 — Movie EV3 [file EMBR-23-e52493-s008.zip › Movie EV3/Movie EV3.docx]

**Movie EV3 - Axon initiation can be separated from axon growth.** 3D reconstructions of confocal time lapse in transverse and lateral views. A DoLA neuron has stereotypical baso-ventral axon initiation. After nascent axon formation, the axon turns and grows rostrally to establish its characteristic axon trajectory. Arrows show axon tip.
