# Supplementary material for: Microtubules are not required to generate a nascent axon in embryonic spinal neurons in vivo
Source: EMBO Rep. 2022 Oct 4;23(11):e52493. doi: 10.15252/embr.202152493 (PMC9638849; doi:10.15252/embr.202152493)
Supplement: Supplementary file 6 — Movie EV4 [file EMBR-23-e52493-s013.zip › Movie EV4/Movie EV4.docx]

**Movie EV4 - Most zebrafish spinal neurons do not undergo apical abscission.** Maximum projection of confocal time lapse, dorsal view. A neuron (asterisk) labelled with a membrane marker retracts its apical process from the apical surface of the spinal cord. No abscission is observed. Solid line shows position of apical surface; arrowheads show tip of retracting apical process.
