# Supplementary material for: Microtubules are not required to generate a nascent axon in embryonic spinal neurons in vivo
Source: EMBO Rep. 2022 Oct 4;23(11):e52493. doi: 10.15252/embr.202152493 (PMC9638849; doi:10.15252/embr.202152493)
Supplement: Supplementary file 7 — Movie EV5 [file EMBR-23-e52493-s005.zip › Movie EV5/Movie EV5.docx]

**Movie EV5 - The centrosome and cilium stay in close proximity and move together towards the basal surface.** Maximum projection of confocal time lapse, dorsal view. Cilia are tagged with GFP and centrosomes are shown in magenta. Arrowheads show one cilium-centrosome pair as it moves from the apical surface towards the basal surface.
