# Supplementary material for: Microtubules are not required to generate a nascent axon in embryonic spinal neurons in vivo
Source: EMBO Rep. 2022 Oct 4;23(11):e52493. doi: 10.15252/embr.202152493 (PMC9638849; doi:10.15252/embr.202152493)
Supplement: Supplementary file 8 — Movie EV6 [file EMBR-23-e52493-s007.zip › Movie EV6/Movie EV6.docx]

**Movie EV6 - The centrosome is not close to the nascent axon during axon initiation.** Transverse reconstruction of confocal time lapse. A neuron is labelled with membrane (grey) and centrosome (green) markers. Open arrows show centrosome position before, during (0 mins) and after axon initiation. Closed arrows show axon tip; asterisk indicates base of axon.
