# Supplementary material for: Microtubules are not required to generate a nascent axon in embryonic spinal neurons in vivo
Source: EMBO Rep. 2022 Oct 4;23(11):e52493. doi: 10.15252/embr.202152493 (PMC9638849; doi:10.15252/embr.202152493)
Supplement: Supplementary file 9 — Movie EV7 [file EMBR-23-e52493-s016.zip › Movie EV7/Movie EV7.docx]

**Movie EV7 - F-actin is persistently localised baso-ventrally before nascent axon initiation.** Transverse reconstruction from confocal time lapse. A neuron is labelled with a membrane marker (grey) and lifeact-Ruby to mark F-actin (green). Frames are every 2 minutes. Actin is persistently localised baso-ventrally before a persistent nascent axon protrusion. Arrowheads at -60 mins show persistent actin localisation, arrows at 0 mins show axon tip.
