# Supplementary material for: Microtubules are not required to generate a nascent axon in embryonic spinal neurons in vivo
Source: EMBO Rep. 2022 Oct 4;23(11):e52493. doi: 10.15252/embr.202152493 (PMC9638849; doi:10.15252/embr.202152493)
Supplement: Supplementary file 10 — Movie EV8 [file EMBR-23-e52493-s004.zip › Movie EV8/Movie EV8.docx]

**Movie EV8 - Microtubule plus-end marker EB3 is not enriched in the nascent axon.** Transverse reconstruction from confocal time lapse. A neuron is labelled with lifeact-Ruby to mark F-actin (greys) and EB3-GFP to mark microtubule plus-ends (green). Most EB3 is located in the cell body before axon initiation and during (0 mins) nascent axon establishment. EB3 is first enriched in the axonal growth cone during axon growth (arrowheads). Arrows show axon tip.
