# Supplementary material for: Microtubules are not required to generate a nascent axon in embryonic spinal neurons in vivo
Source: EMBO Rep. 2022 Oct 4;23(11):e52493. doi: 10.15252/embr.202152493 (PMC9638849; doi:10.15252/embr.202152493)
Supplement: Supplementary file 11 — Movie EV9 [file EMBR-23-e52493-s017.zip › Movie EV9/Movie EV9.docx]

**Movie EV9 - Few microtubule plus-ends enter the nascent axon.** Maximum projection from confocal time lapse, dorsal view. A neuron is labelled with EB3-GFP to mark microtubule plus-ends. Each field shows the same neuron at different timepoints. Frames are every 5 seconds. Compared to the amount of microtubules in the cell body, few microtubule plus-ends grow into the basal-most section of the cell before axon initiation (-30 mins) or into the nascent (0 mins, 30 mins), but do reach the tip of the growing axon (60 mins). Arrows show axon tip.
