# Supplementary material for: Microtubules are not required to generate a nascent axon in embryonic spinal neurons in vivo
Source: EMBO Rep. 2022 Oct 4;23(11):e52493. doi: 10.15252/embr.202152493 (PMC9638849; doi:10.15252/embr.202152493)
Supplement: Supplementary file 12 — Movie EV10 [file EMBR-23-e52493-s003.zip › Movie EV10/Movie EV10.docx]

**Movie EV10 - Axon marker Kif5c560 is enriched in the nascent axon and growth cone.** Transverse reconstruction from confocal time lapse. A neuron is labelled with a membrane marker (grey) and Kif5c560-YFP (yellow). Kif5c560 is localised throughout the cell body before nascent axon initiation, then is gradually enriched in the nascent axon and then growth cone during axon growth. Arrows show axon tip; arrowheads indicate kif5c accumulation.
