# Supplementary material for: Microtubules are not required to generate a nascent axon in embryonic spinal neurons in vivo
Source: EMBO Rep. 2022 Oct 4;23(11):e52493. doi: 10.15252/embr.202152493 (PMC9638849; doi:10.15252/embr.202152493)
Supplement: Supplementary file 13 — Movie EV11 [file EMBR-23-e52493-s014.zip › Movie EV11/Movie EV11.docx]

**Movie EV11 - An actin-rich nascent axon-like protrusion can develop during nocodazole treatment.** Transverse reconstruction from confocal time lapse. A neuron is labelled with a membrane marker (grey) and lifeact-Ruby to mark F-actin (green). The neuron has not yet extended an axon before nocodazole treatment (0 mins). Any small protrusions are retracted upon application of nocodazole (10 mins). During nocodazole treatment the neuron extends multiple small, transient non-axonal protrusions in many directions as well as a longer, persistent, actin-rich nascent axon-like protrusion ventrally (arrows; from 130 mins).
