# Supplementary material for: Microtubules are not required to generate a nascent axon in embryonic spinal neurons in vivo
Source: EMBO Rep. 2022 Oct 4;23(11):e52493. doi: 10.15252/embr.202152493 (PMC9638849; doi:10.15252/embr.202152493)
Supplement: Supplementary file 14 — Movie EV12 [file EMBR-23-e52493-s009.zip › Movie EV12/Movie EV12.docx]

**Movie EV12 - Laminin provides a basal cue for axon initiation.** Transverse reconstruction of confocal time lapse. A neuron is labelled with membrane (grey) and centrosome (green) markers. The axon is extended ventrally while the centrosome is away from the site of axon initiation (0 mins). Asterisk shows position of axon initiation; open arrows show centrosome position; closed arrows show axon tip.
