# Supplementary material for: Microtubules are not required to generate a nascent axon in embryonic spinal neurons in vivo
Source: EMBO Rep. 2022 Oct 4;23(11):e52493. doi: 10.15252/embr.202152493 (PMC9638849; doi:10.15252/embr.202152493)
Supplement: Supplementary file 15 — PDF+ [file EMBR-23-e52493-s019.pdf]

Article

SOURCE DATA | TRANSPARENT PROCESS | OPEN ACCESS

EMBO reports

Microtubules are not required to generate a nascent axon in embryonic spinal neurons *in vivo*

Rachel E Moore[1,*], Sînziana Pop[1,2], Caché Alleyne[1] & Jonathan D W Clarke[1,**]

Abstract

Our understanding of the cell behaviours and cytoskeletal requirements of axon formation is largely derived from *in vitro* models but how these relate to axon formation *in vivo* is not clear. *In vitro*, neurons progress through a well-defined multineurite stage to form an axon and both actin and microtubules cooperate to drive the first steps in neurite and axon morphogenesis. However, these steps are not recapitulated *in vivo*, and it is not clear whether the underlying cell biological mechanisms may differ also. Here, we investigate the mechanisms that regulate axon formation in embryonic zebrafish spinal neurons *in vivo*. We find microtubule organising centres are located distant from the site of axon initiation, and microtubule plus-ends are not enriched in the axon during axon initiation. Focal F-actin accumulation precedes axon formation, and we find that nocodazole-treated neurons with no detectable microtubules are still able to form nascent axonal protrusions that are approximately 10-µm long, dilated and relatively long-lived. We suggest spinal axon formation *in vivo* is fundamentally different from axon formation in *in vitro* models.

Keywords actin; axon initiation; microtubules; neuronal differentiation; zebrafish
Subject Categories Cell Adhesion, Polarity & Cytoskeleton; Development; Neuroscience
DOI 10.15252/embr.202152493 | Received 20 January 2021 | Revised 5 September 2022 | Accepted 8 September 2022 | Published online 4 October 2022
EMBO Reports (2022) 23: e52493

Introduction

During development, neurons polarise by forming an axon and multiple dendrites. This is essential for circuit formation and for the directed propagation of information through the nervous system, but many of the fundamental mechanisms that initiate and build axons and dendrites are not understood. Our current understanding is that the initial appearance of an axonal protrusion requires the interdependent action of both actin and microtubule cytoskeletons (e.g. reviewed in Sakakibara *et al*, 2013; Pacheco & Gallo, 2016). Because observing neuronal morphogenesis at high resolution is technically difficult *in vivo* most of our current understanding of the cell biology of axon initiation is derived from *in vitro* studies. *In vitro*, dissociated rodent hippocampal neurons are spherical until symmetry is broken by the formation of several neurites (Dotti *et al*, 1988). These initial neurites are neither axons nor dendrites. The first step of neuritogenesis is the formation of actin-rich filopodia (Smith, 1994a; Dent *et al*, 2007). An increase in actin dynamics at the cortex then allows microtubule invasion into the filopodia to dilate and consolidate the protrusion (Dent *et al*, 2007; Flynn *et al*, 2012). This can occur via microtubule polymerisation and/or the transport of stable microtubules into the filopodium (Smith, 1994b; Dent *et al*, 2007).

Neuronal polarisation—the segregation of the neuron into axonal and dendritic compartments—then occurs when one neurite is specified to become the axon and grows longer and faster than the others, which subsequently become dendrites (Dotti *et al*, 1988; and reviewed in Barnes & Polleux, 2009). Specification of a neurite into an axon again involves the reorganisation of actin at the neurite tip to allow the invasion of growing microtubules. Microtubule stabilisation precedes and is sufficient to induce axon formation (Kunda *et al*, 2001; Witte *et al*, 2008; and reviewed in Conde & Cáceres, 2009) and the earliest axonal markers that accumulate in the neurite that will become the future axon are microtubule-associated proteins (Jacobson *et al*, 2006; van Beuningen *et al*, 2015). Several signalling pathways that can regulate neuronal polarisation affect microtubule dynamics (Inagaki *et al*, 2001; Shi *et al*, 2003; Ménager *et al*, 2004; Kishi *et al*, 2005; Shelly *et al*, 2007), while others mainly influence the actin cytoskeleton (Kunda *et al*, 2001; Schwamborn & Püschel, 2004). Further, the microtubule and actin cytoskeletal arrays can interact with each other during neuronal development (Geraldo *et al*, 2008; Zhai *et al*, 2017). From these *in vitro* studies, the common mechanistic proposal for both neurite initiation from the cell body and subsequent axon specification from a neurite is one in which actin dynamics lead to microtubule invasion, but both are required to work cooperatively to achieve neurite or axon formation.

We recently showed that *in vivo* morphogenesis of newborn spinal neurons does not follow the same morphogenetic steps involved in axon formation *in vitro*. Spinal neurons *in vivo* undergo a very stereotyped sequence of morphogenesis that includes the generation of a single axonal protrusion directly from the cell body (Hadjivasiliou *et al*, 2019). The neuronal cell bodies move to the

1 Centre for Developmental Neurobiology, Institute of Psychiatry, Psychology and Neuroscience, King's College London, London, UK
2 The Francis Crick Institute, London, UK
*Corresponding author. Tel: +44 2078486416; E-mail: rachel.moore@kcl.ac.uk
**Corresponding author. Tel: +44 2078486463; E-mail: jon.clarke@kcl.ac.uk

© 2022 The Authors. Published under the terms of the CC BY 4.0 license

basal surface of the neuroepithelium while maintaining an attachment to the apical surface and then extend two long, transient protrusions rostrally and caudally along the basal surface of the neural tube. In contrast to the neurites observed in polarising neurons in culture (Dotti *et al*, 1988), these protrusions have stereotyped orientations, are transient and are fully retracted along with the apical attachment prior to axon extension. The axon is then initiated directly from the cell body rather than developing from a pre-existing neurite and well before the appearance of dendrites (Hadjivasiliou *et al*, 2019). Given this difference from the canonical *in vitro* sequence of multiple neurite extension and subsequent axon specification, we decided to assess whether the basic cytoskeletal mechanisms of axon initiation *in vivo* might also be different from those found during neurite initiation *in vitro*.

Here we analyse axon initiation in spinal neurons *in vivo* and test the requirement for microtubules in this mechanism. We focussed on the stage after basal protrusion retraction to investigate what regulates the initiation of axonal outgrowth in the embryonic spinal neurons. We used time-lapse imaging and observed that the axon is consistently initiated from the basal and ventral side of the soma, and microtubule organising centres (MTOCs) are at the opposite side of the cell at the time of axon initiation. Like other examples of axonal or neuritic protrusions, F-actin localises to the future axon initiation site before the accumulation of microtubule plus-ends. However, we also found that microtubules are not required for the formation of nascent axonal protrusions. We propose that for spinal neurons *in vivo*, microtubules are not required for nascent axon establishment but likely do contribute to its stability and dilation. F-actin appears to be the primary cytoskeletal requirement for initial axon development *in vivo*.

Results

Axon initiation *in vivo* is highly stereotyped

To observe neuronal polarisation *in vivo*, we sparsely and randomly labelled zebrafish embryonic spinal cord cells with a membrane marker and imaged them using time-lapse confocal microscopy from 16 h post fertilisation (hpf; Fig 1A). We previously reported that differentiating neurons in the zebrafish spinal cord go through a distinctive and very stereotyped T-shaped morphology before extending an axon (Fig 1B; Movie EV1; Hadjivasiliou *et al*, 2019). To better understand axon initiation, we focussed on the time immediately after retraction of the basal protrusions and apical detachment (19 h 53 min in Fig 1B). At this initial stage neurons had no prominent or long-lasting protrusions but extended small, transient protrusions and filopodia (Fig 1C: −2 h to −20 min; Movie EV2). These transient protrusions occurred in many directions at first (Fig 1C: −1 h 30 min) but were gradually restricted to a more defined baso-ventral position on the cell body. Each neuron then extended a single dilated axonal protrusion from this position (Fig 1C: 0 h). This protrusion was much larger and more stable than the earlier filopodia, and after formation, its growth often paused for approximately 20–30 min (Figs 1C: 0 h to 30 min and EV1) suggesting this represents a distinct phase of axon initiation. After pausing, the axon extended rapidly and developed a growth cone (Fig 1C: 45 min to 2 h 20 min). We defined the time of axon initiation as the first time point that showed a persistent, dilated protrusion that subsequently matured into an axon with a growth cone (Fig 1C: 0 h). We call this large persistent protrusion the nascent axon. We defined the site of axon initiation as the position where the nascent axon protrusion emerges from the cell body at this time point (Fig 1C: 0 h, asterisk). The position of the nascent axon was analysed with respect to the cell centroid and found to be highly biased towards the baso-ventral quadrant of the soma (Fig 1D).

Several different projection neuron subtypes arise during the first few hours of neurogenesis in the embryonic zebrafish spinal cord (Bernhardt *et al*, 1990; Hale *et al*, 2001). They can be distinguished by the dorso-ventral position of the soma within the spinal cord together with their axon trajectory (Fig 1E). Our cell labelling method randomly targeted all of the early embryonic neuronal subtypes reported previously (Appendix Fig S1A; Bernhardt *et al*, 1990; Hale *et al*, 2001). Of the 86 neurons that we analysed for the site of axon initiation (Fig 1D), we were able to classify 53 by neuronal

Figure 1. Axon initiation is highly stereotyped and occurs at the baso-ventral aspect of the soma irrespective of subsequent axon trajectory.

A Diagram to illustrate the three different views shown in confocal images and 3D reconstructions throughout this paper, plus legend for arrows and asterisk.

B Image sequence from confocal time lapse shows the early steps in neuronal differentiation. Two transient basal protrusions are extended along the basal surface of the neural tube (6 h 26 min to 14 h 01 min) and then retracted (15 h 31 min to 17 h 31 min). The apical attachment is also retracted (17 h 31 min to 19 h 53 min; e.g. −1 h 30 min) before the axon is extended (20 h 38 min to 21 h 50 min). Main images are maximum projections and insets are transverse reconstructions from confocal z-stacks.

C Image sequence from confocal time lapse shows a neuron before, during and after axon initiation. Prior to axon initiation, the neuron extends multiple small, transient preaxonal protrusions (−2 h to −20 min). The nascent axon is extended (0 h) and maintained for a short period (0 h to 30 min) before axon growth begins (45 min to 2 h 20 min). Images are transverse reconstructions from confocal z-stacks.

D Plots showing axon position on the soma (e.g. asterisk in Fig 1C: 0 h) relative to the cell centroid at 0,0 for dorsal and transverse views (n = 86 cells from 8 experiments). Axon position is not random (dorsal view $P < 0.001$, mean = 95.3°; transverse view $P < 0.001$, mean = 148.9°). Data analysed using Moore's modification of the Rayleigh's test.

E Diagram showing neuronal subtypes in the zebrafish embryo's spinal cord. CiA, circumferential ascending; CiD, circumferential descending; CoB, commissural bifurcating; CoPA, commissural primary ascending; CoSA, commissural secondary ascending; DLF, dorsal longitudinal fasciculus; DoLA, dorsolateral ascending; M, motor; MLF, medial longitudinal fasciculus; RB, Rohon-Beard; UCoD, unilateral commissural descending; VeLD, ventral longitudinal descending; VLF, ventral longitudinal fasciculus.

F Lateral and transverse reconstructions of DoLA neurons at the time of axon initiation (0 h) and during axon growth (4 h).

G–M Plots showing axon position on the soma relative to cell centroid at 0,0 in dorsal and transverse views for DoLA (G; n = 7 cells), CiA (H; n = 10 cells), CiD (I; n = 3 cells), VeLD (J; n = 5 cells), CoSA (K; n = 11 cells), CoB (L; n = 6 cells) and UCoD (M; 15 cells) neuronal subtypes.

Data information: All scale bars = 10 µm.
Source data are available online for this figure.

 © 2022 The Authors

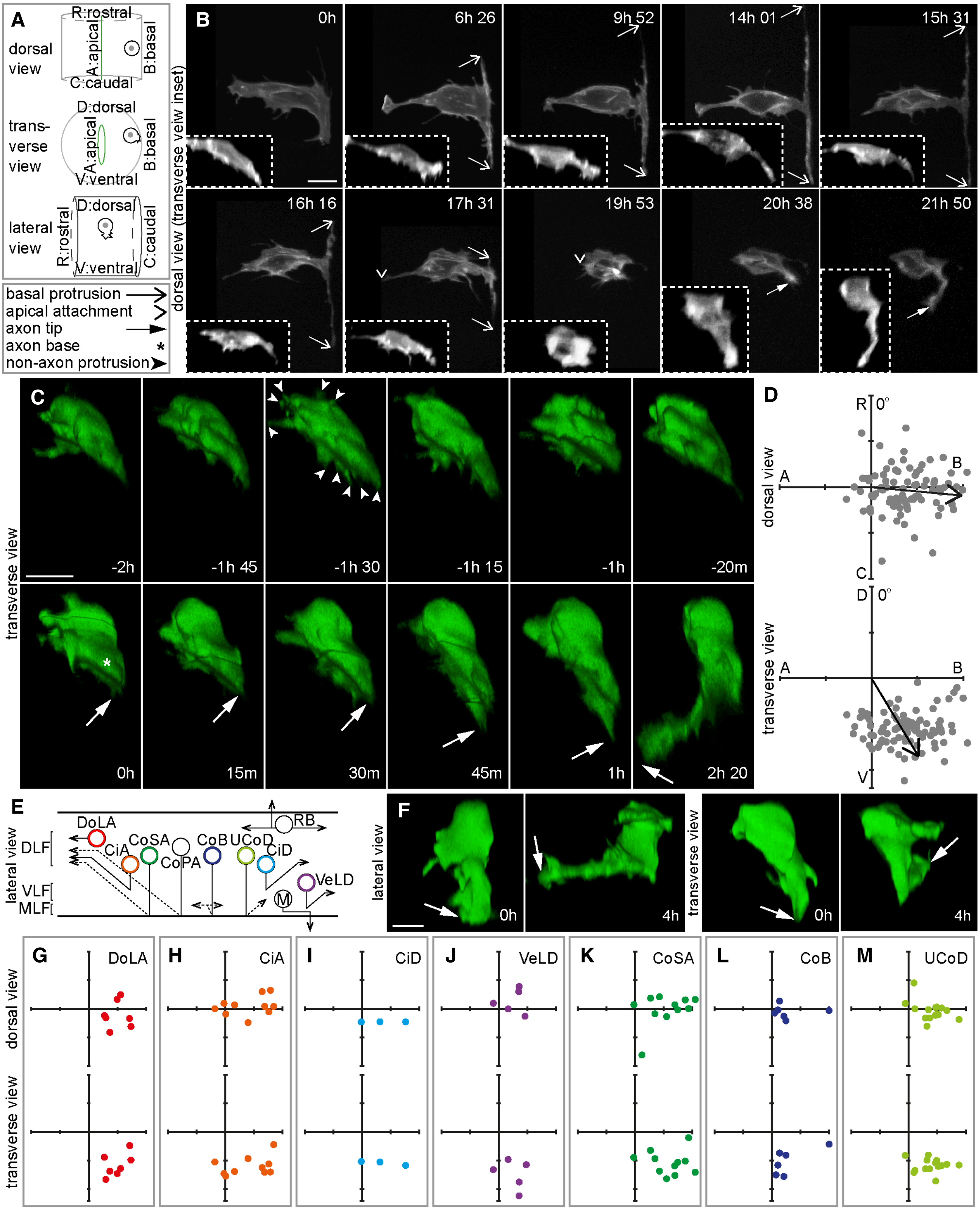
A
dorsal view
R:rostral
A:apical
B:basal
C:caudal
trans-verse view
D:dorsal
V:ventral
lateral view
basal protrusion
apical attachment
axon tip
axon base
non-axon protrusion
B
dorsal view (transverse veiw inset)
0h
6h 26
9h 52
14h 01
15h 31
16h 16
17h 31
19h 53
20h 38
21h 50
C
transverse view
-2h
-1h 45
-1h 30
-1h 15
-1h
-20m
0h
15m
30m
45m
1h
2h 20
D
dorsal view
transverse view
R 0°
C
D 0°
V
A
B
E
lateral view
DoLA
CiA
CoSA
CoPA
CoB
UCoD
CiD
RB
VeLD
M
DLF
VLF
MLF
F
lateral view
transverse view
0h
4h
G DoLA
H CiA
I CiD
J VeLD
K CoSA
L CoB
M UCoD

Figure 1.

subtype (Fig 1E and F; Appendix Fig S1B–G). The axons of many neuronal subtypes grow ventrally and circumferentially before projecting either to the contralateral side of the spinal cord (e.g. CoSA; Appendix Fig S1E) or ipsilaterally (e.g. circumferential ascending [CiA] neurons; Appendix Fig S1B). We find the site of nascent axon formation was baso-ventral for all of these neuronal subtypes (Fig 1H–M). The only neuronal subtype whose axons are not circumferential is dorsal lateral ascending (DoLA) neurons, which project their axons rostrally towards the hindbrain (Fig 1E; Bernhardt *et al*, 1990). Surprisingly, however, DoLA neurons also had a baso-ventral location for their nascent axon (Fig 1F transverse view 0 h, G; Movie EV3). Time-lapse imaging showed that, after basolateral nascent axon formation, the axon turned and grew rostrally to establish its characteristic axon trajectory (Fig 1F: lateral view 4 h; Movie EV3).

Altogether, this confirms and quantifies our previous observation that axon initiation occurs directly from the neuronal soma (Hadjivasiliou *et al*, 2019). The first persistent axonal protrusion, which we define as the nascent axon, is formed at a stereotyped baso-ventral position common to all neuronal subtypes. That this is consistent regardless of the subsequent axonal trajectory shows that axon initiation in the zebrafish spinal cord is a separate process that can be decoupled from axonal growth and guidance.

Centrosome behaviour prior to axon initiation

Previous studies have suggested that centrosome position is important for positioning the axon (de Anda *et al*, 2005; Andersen & Halloran, 2012). To get a comprehensive analysis of the centrosome leading up to and during axon initiation *in vivo* we monitored the centrosome position from its initial location at the apical surface of the neuroepithelium through to the time of nascent axon establishment. We first focussed on the time when the neuron detaches from the apical surface (see Fig 1B: 17 h 31 min to 19 h 53 min). In *ex ovo* chick neural tube slices the centrosome remains at the apical surface during neuronal differentiation until the abscission of the apical processes, when the centrosome is retracted back to the soma along with the apical process. The retracting process abscises from the apical endfoot, which is left behind at the apical surface of the neural tube along with the cilium (Das & Storey, 2014). We too found that the centrosome in zebrafish spinal cord cells is retracted along with the apical process (Fig 2A). However, unlike the chick spinal cord, most zebrafish spinal neurons do not show any apical abscission events (Fig 2B; Movie EV4). We next used a zebrafish transgenic line with GFP-tagged cilia (Tg(actb2:arl13b-GFP)) and observed cilia in the zebrafish spinal cord during a period when we know that many neurons will be retracting their apical processes. We saw many examples of cilia moving from the apical surface to close to the basal surface (Fig 2C), reminiscent of apical retraction. In several instances, we could follow a particular cilium continually for up to 45 min before it left the apical surface and then moved basally (n = 13 cilia). Cilium length did not change either while at the apical surface or while moving towards the basal surface (Fig 2E). These data show that in zebrafish the cilia are retained by most spinal neurons during apical retraction rather than being abscised and regrown. Finally, we observed centrosomes and cilia at the same time by labelling centrosomes in the zebrafish cilium line. Time-lapse videos showed that the centrosome and cilia stay in close proximity both at the apical surface and as they moved together towards the basal surface (Fig 2D; Movie EV5). Immunohistochemistry also showed cilia and centrosomes close together in several positions along the apico-basal axis of the spinal cord, and close proximity was maintained no matter where they were along this axis (Fig 2F). Altogether this data shows that the centrosome and cilia do not physically dissociate but are retracted together within the apical process to the soma during the large majority of neuronal differentiation events in the zebrafish spinal cord. Since it travels in the apical pole of the retracting process, the centrosome locates in the apical pole of the neuronal soma at the end of this phase of differentiation.

The centrosome is located on the opposite side of the cell to the nascent axon

Once apical retraction was completed the centrosome was close to the apical pole of the neuronal soma. To assess whether the proximity of the centrosome is involved in axon initiation we next analysed centrosome position in neurons during the establishment of the

Figure 2. The centrosome and cilium are retracted to the soma during apical process retraction.

A Image sequence showing the centrosome position of a neuron labelled with membrane and centrosome labels during apical process retraction. The centrosome is retracted close to the tip of the apical process. Images are maximum projections from confocal z-stacks.

B (i) Image sequence showing a neuron labelled with a membrane marker during apical retraction without any observable abscission event (n = 64/72 cells from 6 experiments). (ii) High-resolution images of boxed section in (i). Images are maximum projections from confocal z-stacks.

C (i) Low magnification overview of the spinal cord of a cilium reporter line, Tg(actb2:arl13b-GFP) from a single confocal slice. (ii) High-resolution image sequence of boxed section in (i). One GFP-labelled cilium moves from apical surface towards the basal surface of the spinal cord (n = 13 cilia from two experiments). Images are maximum projections from confocal z-stacks.

D (i) Low magnification overview of the spinal cord of a Tg(actb2:arl13b-GFP) embryo labelled with centrin-RFP from a single confocal slice. (ii) Image sequence of boxed section in (i). A cilium and centrosome move together from apical surface towards the basal surface of the spinal cord (n = 2 cells from one experiment). Insets show high magnification of cilium-centrosome pair. Images are maximum projections from confocal z-stacks.

E Graph showing the length of cilium as it moves from apical surface (0%) to close to the basal surface (100%. Cilium length did not change; $P > 0.05$ for n = 6/7 cilia from two experiments; nonlinear regression).

F Distance between centrosome and cilium in Tg(actb2:arl13b-GFP) embryos fixed and processed for immunohistochemistry against GFP to label the cilium and γ-tubulin to label the centrosome. No difference was found in the distance between the two organelles when close to the apical surface or away from the midline (n = 50 cells per position from two experiments; P = 0.8279; midline mean = 0.4956, s.d. = 0.1125; away from midline mean = 0.5004, s.d. = 0.1077; Student's unpaired t-test). Bars show mean and standard deviation.

Data information: All scale bars = 10 μm.
Source data are available online for this figure.

 © 2022 The Authors

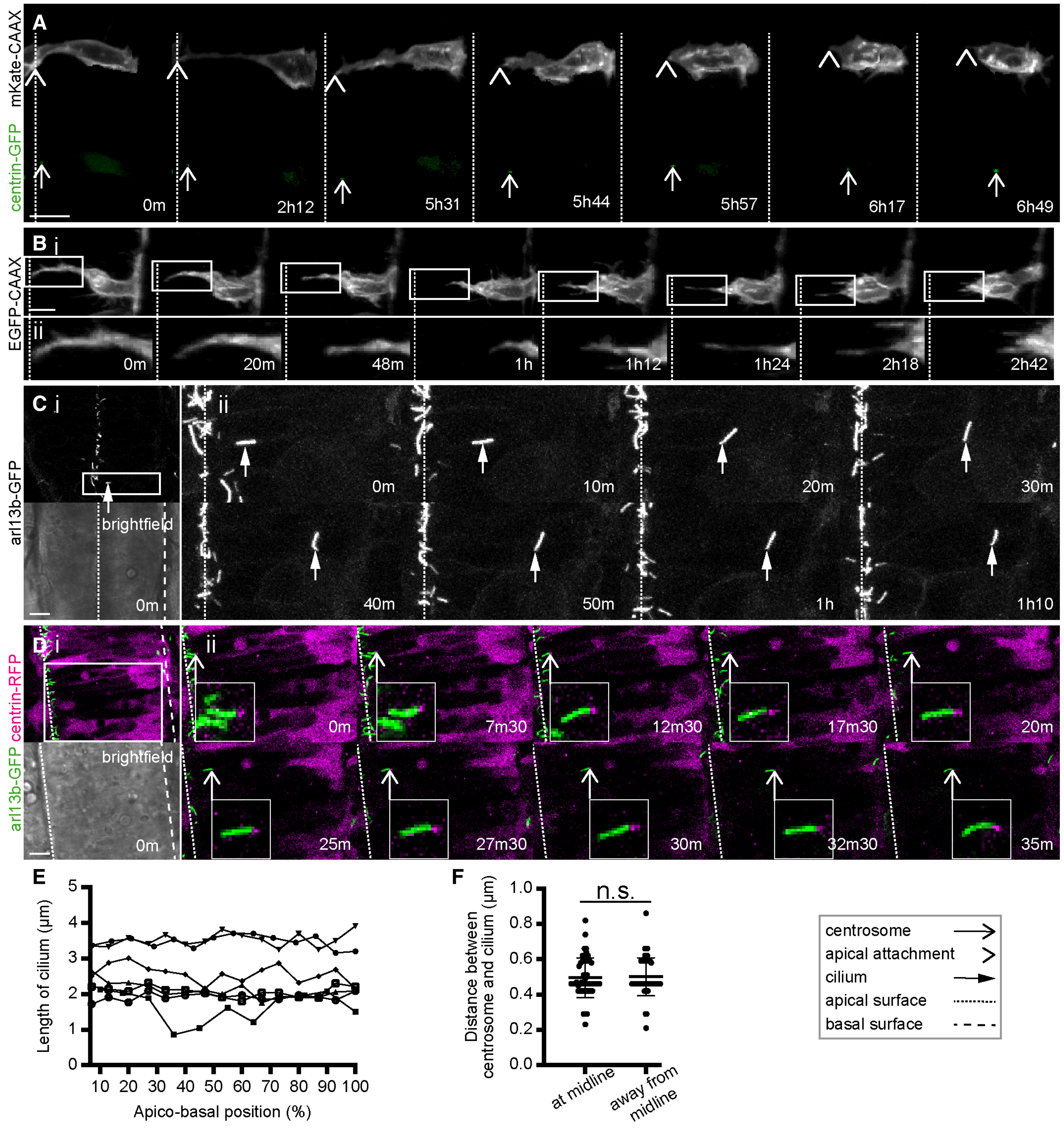

Figure 2.

nascent axon. Time-lapse imaging showed that the centrosome was not close to the position of the nascent axon (Fig 3A: 0 m; Movie EV6). When the nascent axon is first identifiable, the mean distance between the centrosome and nascent axon was 10.1 µm ± 3.3 (Fig 3C). To put this into perspective, the mean diameter of these cells' nuclei was 7.8 µm (Fig 3C: dotted line, s.d. = 0.7, n = 5 cells). To quantify the spatial relationship between the centrosome and nascent axon we analysed their positions with respect to the cell centroid at the time of axon initiation. The centrosome position was highly biased towards the apico-dorsal side of the cell (Fig 3F green dots), placing it on the opposite side of the cell to the baso-ventral site of the nascent axon (Fig 3F grey dots). Further, paired analysis of the positions of the centrosome and nascent axon in the same cell showed that these were different (Fig 3F). Finally, we analysed the

© 2022 The Authors

slope of vectors linking the centrosome and nascent axon of each cell. The centrosome-axon axis was strongly oriented from apical to basal in the dorsal view and from apico-dorsal to baso-ventral in the transverse view (Fig 3F). These results show that the centrosome is not close to the site of the nascent axon in zebrafish spinal cord neurons *in vivo*; indeed, it is normally on the opposite side of the cell. The centrosome is deposited apically and dorsally following apical retraction and remains in that quadrant of the neuron until after axon initiation.

These results for the projection neurons of the spinal cord seem to be at odds with a previous report suggesting the centrosome was close to the site of peripheral axon initiation in primary sensory Rohon-Beard neurons in zebrafish embryos (Andersen & Halloran, 2012). Rohon-Beard neurons extend three axons—ascending, descending and peripheral. We analysed the centrosome position in Rohon-Beard neurons in relation to each of these axons (Fig EV2A). In 22 of 23 events analysed, the centrosome was more than 10 μm from the site of axon initiation (Fig EV2B). When centrosome and axon positions were assessed with respect to the cell centroid, the centrosome was not close to the site of axon initiation but was located on the apical side of the soma when the ascending and descending axons were initiated, as previously reported (Fig EV2C;

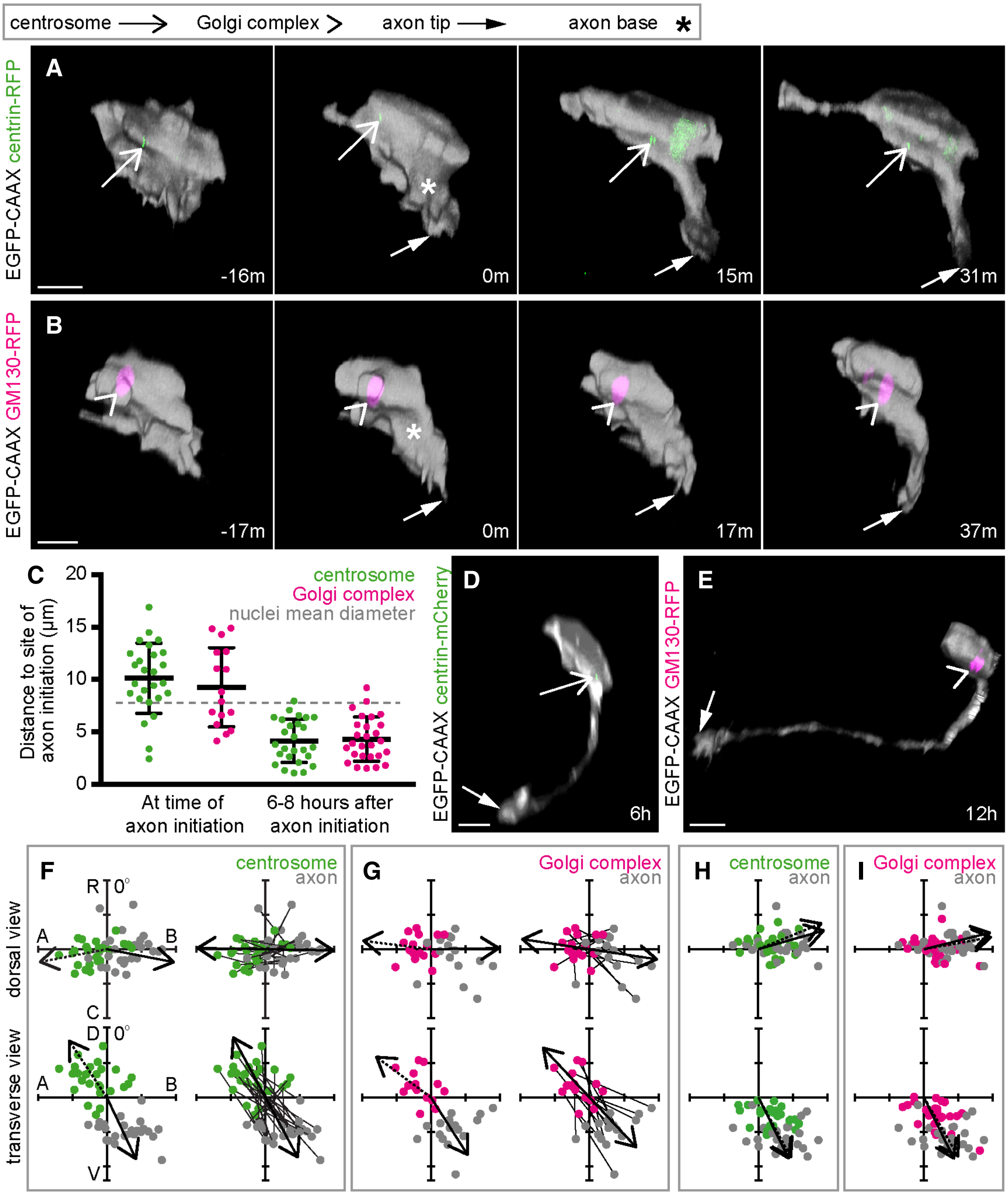

Figure 3.

 © 2022 The Authors

Figure 3. MTOCs are positioned on the opposite side of the cell to the nascent axon.

A Image sequence from confocal time lapse shows a neuron labelled with membrane and centrosome markers before (−16 m), during (0 m) and after axon initiation (15 m, 31 m). The centrosome is located on the opposite side of the cell to the nascent axon. Images are transverse reconstructions from confocal z-stacks.

B Image sequence from confocal time lapse shows a neuron labelled with membrane and Golgi complex markers before (−17 m), during (0 m) and after axon initiation (17 m, 37 m). The Golgi complex is located on the opposite side of the cell to the nascent axon. Images are transverse reconstructions from confocal z-stacks.

C Graph showing distance between centrosome or Golgi complex and base of axon at time of axon initiation and 6–8 h after axon initiation. Bars show mean and standard deviation. At time of axon initiation: centrosome-axon mean = 10.1 μm, s.d. = 3.3, n = 26 cells from three experiments; Golgi complex-axon mean = 9.3 μm, s.d. = 3.8, n = 16 cells from two experiments. At 6–8 h after axon initiation: centrosome-axon mean = 4.2 μm, s.d. = 2.1, n = 26 cells; Golgi complex-axon mean = 4.3 μm, s.d. = 2.1, n = 27 cells.

D Transverse reconstruction from confocal time lapse of a neuron labelled with membrane and centrosome markers during axon growth.

E Transverse reconstruction from confocal time lapse of a neuron labelled with membrane and Golgi complex markers during axon growth.

F Plots showing the positions of the centrosome and base of the axon at the time of axon initiation relative to the cell centroid at 0,0 for dorsal and transverse views (n = 26 cells from three experiments). Left-hand plots: centrosome position is not random (dorsal view $P < 0.001$, mean = −100.8°; transverse view $P < 0.001$, mean = −33.7°) and axon position is not random (dorsal view $P < 0.001$, mean = 101.0°; transverse view $P < 0.001$, mean = 154.8°; Moore's modification of the Rayleigh's test). Centrosome and axon positions are significantly different (dorsal view $0.001 > P$; transverse view $0.001 > P$; Moore's test for paired data). Right-hand plots: vectors linking centrosome and nascent axon of the same cell are not random (dorsal view $P < 0.001$, mean = 91.0°; transverse view $P < 0.001$, mean = 151.7°; Moore's modification of the Rayleigh's test).

G Plots showing the positions of the Golgi complex (magenta) and base of the axon (grey) at the time of axon initiation relative to the cell centroid at 0,0 for dorsal and transverse views (n = 16 cells from two experiments). Left-hand plots: Golgi complex position is not random (dorsal view $0.01 < P < 0.05$, mean = −83.0°; transverse view $P < 0.001$, mean = −53.4°) and axon position is not random (dorsal view $P < 0.001$, mean = 89.1°; transverse view $P < 0.001$, mean = 147.4°; Moore's modification of the Rayleigh's test). Golgi complex and axon positions are significantly different (dorsal view $0.001 > P$; transverse view $0.001 > P$; Moore's test for paired data). Right-hand plots: vectors linking Golgi complex and nascent axon of the same cell are not random (dorsal view $P < 0.001$, mean = 98.5°; transverse view $P < 0.001$, mean = 156.8°; Moore's modification of the Rayleigh's test).

H Plots showing the positions of the centrosome and base of the axon 6–12 h after axon initiation relative to the cell centroid at 0,0 for dorsal and transverse views (n = 26 cells). Centrosome position is not random (dorsal view $P < 0.001$, mean = 67.7°; transverse view $P < 0.001$, mean = 151.6°). Axon position is not random (dorsal view $P < 0.001$, mean = 74.0°; transverse view $P < 0.001$, mean = 153.7°; Moore's modification of the Rayleigh's test). Centrosome and axon positions are not significantly different (dorsal view $0.5 < P$; transverse view $0.1 < P < 0.5$; Moore's test for paired data).

I Plots showing the positions of the Golgi complex and base of the axon 6–12 h after axon initiation relative to the cell centroid at 0,0 for dorsal and transverse views (n = 27 cells). Golgi complex position is not random (dorsal view $P < 0.001$, mean = 79.9°; transverse view $P < 0.001$, mean = 149.9°). Axon position is not random (dorsal view $P < 0.001$, mean = 76.0°; transverse view $P < 0.001$, mean = 153.9°; Moore's modification of the Rayleigh's test). Golgi complex and axon positions are different only in transverse view (dorsal view $0.5 < P$; transverse view $0.005 < P < 0.01$; Moore's test for paired data).

Data information: All scale bars = 10 μm.
Source data are available online for this figure.

Andersen & Halloran, 2012). When the peripheral axon was initiated, the centrosome was closer to the cell centroid than to the axon for every cell analysed. The rostral-caudal position of the centrosome did not appear to correlate with the rostral-caudal position of the site of peripheral axon initiation (Fig EV2C dorsal view), further indicating that the centrosome is not close to the site of peripheral axon during initiation. Thus, although the centrosome moves towards the basal side of Rohon-Beard neurons during peripheral axon initiation and growth (Fig EV2A: 9 h; Andersen & Halloran, 2012), it is not close to the site of axon initiation when an axon is first extended.

The Golgi complex is also located on the opposite side of the cell to the nascent axon

The Golgi complex can also nucleate microtubules (Chabin-Brion *et al*, 2001) and has been reported to be close to the neurite that becomes the axon *in vitro* (de Anda *et al*, 2005). As such, it could potentially act as an alternative MTOC independently of the centrosome. To investigate this, spinal cord cells were randomly labelled with a membrane marker and a Golgi complex marker (GM130-RFP or -GFP). Time-lapse analysis showed the Golgi complex was not close to the site of nascent axon formation (Fig 3B: 0 min and C). Like the centrosome, the position of the Golgi complex was biased towards the apico-dorsal side of the cell (Fig 3G magenta dots), on the opposite side of the cell to the nascent axon (Fig 3G grey dots). The Golgi complex-axon axis was strongly oriented from apical to basal in the dorsal view and from apico-dorsal to baso-ventral in the transverse view (Fig 3G). These results show that the Golgi complex is also not close to the site of axon initiation *in vivo*.

Finally, we used immunohistochemistry to investigate the γ-tubulin location. γ-tubulin is highly likely to be required for microtubule nucleation in cells and so marks any potential MTOC (Moritz & Agard, 2001). We could only find obvious γ-tubulin accumulation at one concentrated point in each neuron that appeared to correspond with the centrosome (Appendix Fig S2A and B). Along with analysis of centrosome and Golgi positioning, these results suggest that there is no potential MTOC close to the site of axon initiation.

Both centrosome and Golgi move to the base of the axon after its initiation

Some previous studies have shown that the centrosome and Golgi complex are located at the base of axons in cell culture (de Anda *et al*, 2005; Stiess *et al*, 2010) and the centrosome moves towards the basal side of the cell during peripheral axon extension in Rohon-Beard neurons (see Fig EV2; Andersen & Halloran, 2012). We hypothesised that the proximity of these MTOCs to the axon may reflect axon growth rather than axon initiation, so we analysed the position of the centrosome and Golgi complex during axon pathfinding between 6 and 12 h after the establishment of the nascent axon (Fig 3D and E). We found that both organelles had moved close to the base of the axon during axon growth (Fig 3C). The position of all of these organelles was at the baso-ventral side of the cell and

was not random (Fig 3H and I). Paired analysis of the positions of the centrosome or Golgi complex and base of the axon in the same cell showed that these were not different in most cases (Fig 3H and I). Altogether our results show MTOCs are closely related to the base of the axon during axon growth but not during axon initiation.

Growing microtubule plus-ends are not enriched in the nascent axon

Although the location of MTOCs is not close to the site of nascent axon initiation it is still possible that microtubules accumulate at the site of axon initiation to specify this location. Alternatively, actin accumulation may precede microtubules to specify this position. To identify the sequence in which these cytoskeletal elements associate with the site of nascent axon formation *in vivo* we have compared the relative positions of F-actin, growing microtubules and the microtubule motor Kif5c during nascent axon formation.

We find F-actin is the earliest cytoskeletal element to mark the site of the nascent axon. F-actin accumulation has previously been shown to be an early indicator of the site of neurite, axon and collateral branch initiation *in vitro* (Gallo & Letourneau, 1999; Dent *et al*, 2007; Witte *et al*, 2008), as well as axon initiation in Rohon-Beard neurons in zebrafish (Andersen & Halloran, 2012). In spinal neurons, co-labelling with a membrane probe and lifeact-Ruby showed that F-actin was persistently enriched baso-ventrally in advance of the persistent protrusion of the nascent axon (Fig 4A; Appendix Fig S3; Movie EV7; n = 14/15 cells), often more than 30 min before nascent axon protrusion (Fig 4B; 10/15 cells). F-actin then remains distinctly enriched at the distalmost tips of the extending nascent axon for at least 60 min after nascent axon initiation (Fig 4A).

To understand the distribution of dynamic microtubules, we imaged EB3-GFP before, during and after axon initiation. EB3 is a microtubule plus-end binding protein that labels the tips of all growing microtubules. We found that EB3 was localised throughout the cell before axon initiation (Fig 4C). In contrast to F-actin, however, most EB3 was located in the soma during nascent axon establishment and only a few microtubule plus-ends were localised within the nascent axon itself (Fig 4C: 0 min; Appendix Figs S4 and S5A; Movie EV8). Close examination of EB3 before and during nascent axon formation showed that very few microtubule plus-ends grew from the cell body into the newborn protrusion compared with the amount of growing microtubules in the cell body (Appendix Fig S5B; Movie EV9). However, EB3 was subsequently enriched in the axonal growth cone during axon growth (Fig 4C: 50 min). To compare the timing of actin accumulation and the invasion of growing microtubules into the nascent axon we analysed the distribution of filamentous actin (F-actin) and EB3 simultaneously. Simultaneous imaging showed F-actin is enriched in the nascent axon in advance of EB3-GFP enrichment (Fig 4C: −10 min; Appendix Fig S5; n = 6/8 cells).

To examine whether specific microtubule-dependent traffic might be enriched in the nascent axon we imaged a constitutively active version of the kinesin 1 motor domain, Kif5c560, that is trafficked specifically on axonal microtubules and is an early axonal marker *in vitro* and *in vivo* (Jacobson *et al*, 2006; Randlett *et al*, 2011). In newborn neurons, Kif5c560 was fairly evenly localised throughout the cell body before nascent axon establishment and was present but not enriched in the nascent axon at the time of its establishment (Fig 4D: 0 min). It does become enriched in the nascent axon after nascent axon establishment and remains enriched in the growth cone during axonal growth (Fig 4D; Appendix Figs S6 and S7; Movie EV10; n = 10/13 cells).

To confirm the sequence of cytoskeletal enrichment in the nascent axon we imaged Kif5c560-YFP, lifeact-Ruby and EB3-GFP in the same neurons. This showed that F-actin enrichment in the nascent axon was more distal than that of both Kif5c560 and EB3-GFP (Fig 4E; Appendix Figs S3–S8, n = 3/4 cells). In total, these observations show F-actin accumulates at the site of the nascent axon before it is stabilised, precedes any enrichment of growing microtubules in the nascent axon and is the earliest cytoskeletal sign of specification of nascent axon formation *in vivo*.

Nascent axons form in the absence of microtubules

The finding that F-actin accumulation consistently preceded the formation of the nascent axon and was enriched in the nascent axon distal to the enrichment of both the microtubule motor kif5c560 and growing microtubules, suggests microtubules may not be the primary cytoskeletal elements necessary for axon initiation *in vivo*. To test this, we examined axon initiation in nocodazole-treated cells with no detectable microtubules. We labelled cells for membrane and F-actin and bathed embryos in 5 µg/ml nocodazole for 60–180 min to depolymerize microtubules (Head *et al*, 1985; Jordan & Wilson, 1998; Gallo & Letourneau, 1999). By 30 min EB3-GFP-labelled comets that label growing microtubules had disappeared from cells (Appendix Fig S9B) and 45 min after nocodazole addition immunohistochemistry against α-tubulin showed that the

Figure 4. Actin accumulation precedes enrichment of microtubule plus-ends during nascent axon formation.

A Image sequence from confocal time lapse of a neuron labelled with a membrane marker and lifeact-Ruby before, during (0 m) and after axon initiation. Images are transverse reconstructions from confocal z-stacks.

B Graph showing time (minutes) between actin accumulation and nascent axon initiation (n = 15 cells from 7 experiments). Bars show mean and standard deviation.

C Image sequence from confocal time lapse of a neuron labelled with lifeact-Ruby and EB3-GFP before, during (0 m) and after axon initiation. Images are transverse reconstructions from confocal z-stacks.

D Image sequence from confocal time lapse of a neuron labelled with a membrane marker and Kif5c560-YFP before, during (0 m) and after axon initiation. Images are transverse reconstructions from confocal z-stacks.

E Three time points from confocal time lapse of a triple labelled neuron before, during (0 m) and after nascent axon initiation. Images to left show the distribution sequence of lifeact-Ruby, kif5c560-YFP and EB3-GFP individually. Dual channel merges to the right show relative locations of pairs of fusion proteins. Images to right are maximum projections of transverse reslices of confocal z-stacks.

Data information: All scale bars = 10 µm.
Source data are available online for this figure.

 © 2022 The Authors

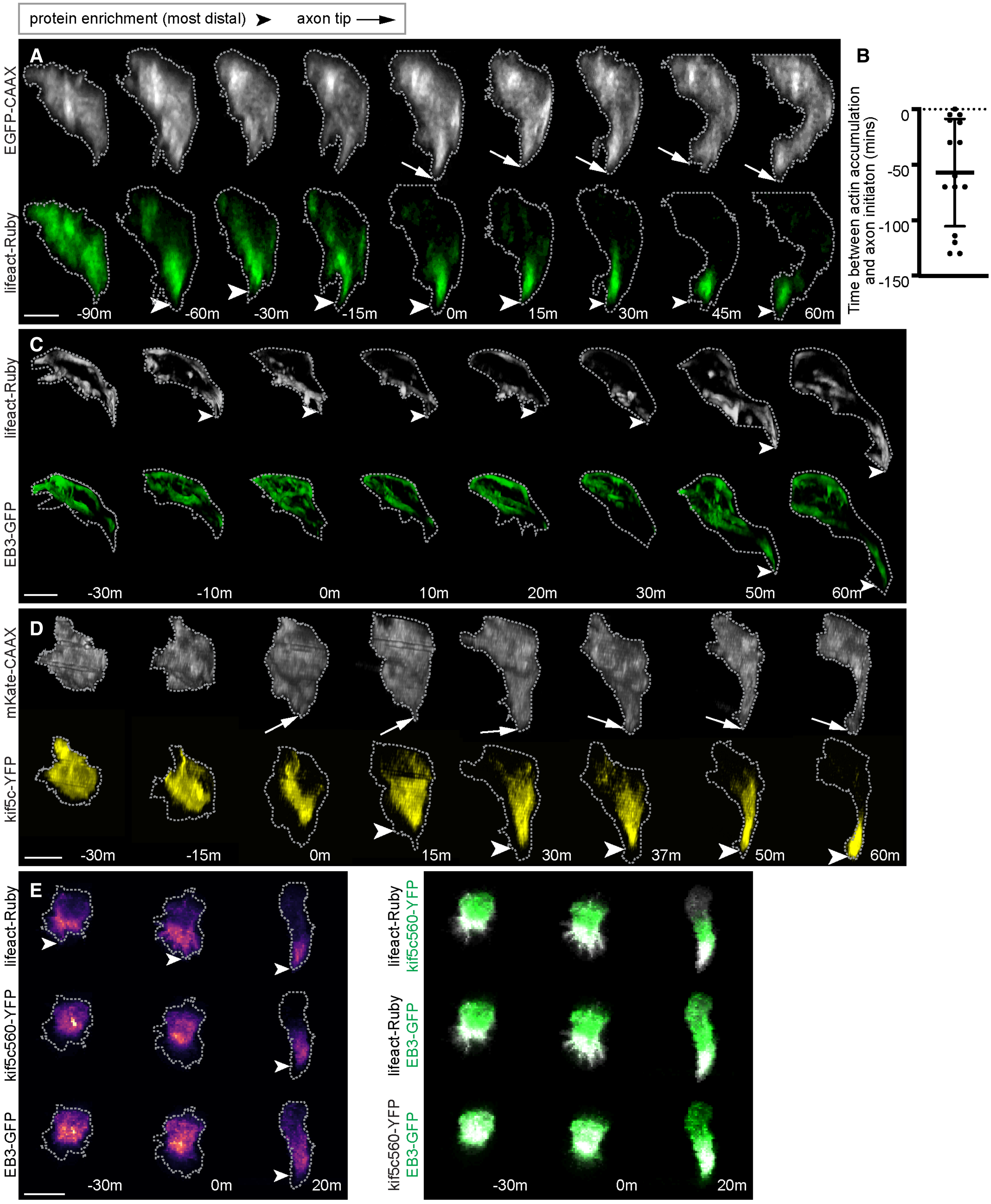

protein enrichment (most distal)
axon tip
A
EGFP-CAAX
lifeact-Ruby
-90m
-60m
-30m
-15m
0m
15m
30m
45m
60m
B
Time between actin accumulation and axon initiaton (mins)
0
-50
-100
-150
C
lifeact-Ruby
EB3-GFP
-30m
-10m
0m
10m
20m
30m
50m
60m
D
mKate-CAAX
kif5c-YFP
-30m
-15m
0m
15m
30m
37m
50m
60m
E
lifeact-Ruby
kif5c560-YFP
EB3-GFP
-30m
0m
20m
lifeact-Ruby kif5c560-YFP
lifeact-Ruby EB3-GFP
kif5c560-YFP EB3-GFP

Figure 4.

whole filamentous microtubule array is completely disrupted in newborn neurons and neuroepithelial cells (Appendix Fig S9A). We then analysed whether nascent axons could form after 45 min of nocodazole treatment, in the absence of both dynamic and stable microtubules.

We focussed on 54 neurons that had retracted their apical and basal processes and had not yet extended a nascent axon prior to nocodazole addition. Most of these cells completely retracted any small protrusions or filopodia upon nocodazole treatment (45/54 cells). However, all neurons then developed multiple short, thin, transient protrusions during nocodazole treatment (Fig 5A–E); we call these nonaxonal protrusions. They were reminiscent of preaxonal protrusions in control cells (see Figs 1C: −1 h 30 min and 5C–E). Nonaxonal protrusions were often extended from multiple locations on the cell, either consecutively or sequentially, but the vast majority protrude from the ventral side of the cell (Fig 5A, H, and I). This suggests cells can either still respond to ventral cues or else maintain their polarity in the absence of microtubules. During nocodazole treatment, a quarter (14/54) of imaged neurons developed a *de novo* protrusion, enriched with F-actin and with characteristics reminiscent of a nascent axon (Fig 5B; Movie EV11). These protrusions, like nascent axons on normal neurons (see Fig 1D), were predominantly initiated from the baso-ventral quadrant of the cell and were longer and wider and more persistent than nonaxonal protrusions, lasting at least 20 min and up to 90 min, or until the end of nocodazole treatment (Fig 5C–G). Compared with normal nascent axon protrusions, those in nocodazole-treated cells were shorter, less persistent and less dilated (Fig 5C–E). To assess the efficiency with which nocodazole-treated neurons produce nascent axons, we analysed how many control neurons produced nascent axons when imaged for a similar time period (135 min). We found 19/30 (63%) control neurons produced a nascent axon within 135 min of imaging, compared with 14/54 (26%) neurons in nocodazole-treated embryos. In contrast to control neurons, the microtubule motor Kif5c560 was not enriched in the axon-like protrusions in nocodazole-treated neurons (Fig 5J and K), supporting the view that nocodazole disrupted microtubule-based cargo in nascent axon protrusions. These results show microtubules are not required for the establishment of a nascent axon, although our results suggest they are important for its subsequent dilation and stabilisation. Together with our results showing that F-actin localisation preceded that of microtubule markers, this suggests that F-actin accumulation is the key cytoskeletal element for nascent axon initiation.

Figure 5. Nascent axons form in the presence of nocodazole, a microtubule polymerisation inhibitor.

A Image sequence from confocal time lapse of a neuron that does not extend a nascent axon during nocodazole treatment labelled with a membrane marker and lifeact-Ruby before (−10 m) and during (0 m to 170 m) nocodazole treatment. Protrusions present before nocodazole addition are retracted upon nocodazole treatment (20 m). Short, transient nonaxonal protrusions are extended during nocodazole treatment. Images are transverse reconstructions from confocal z-stacks.

B Image sequence from confocal time lapse of a neuron that extends a nascent axon during nocodazole treatment labelled with a membrane marker and lifeact-Ruby before (−10 m) and during (0 h to 170 min) nocodazole treatment. Small protrusions present before nocodazole addition (−10 m) are retracted upon nocodazole treatment (0 m). A nascent axon-like protrusion (long, broad, long-lived) is extended during nocodazole treatment (110 m to 170 m). Transient nonaxonal protrusions are also present. Images are transverse reconstructions from confocal z-stacks.

C Graph showing duration (minutes) of nascent axon-like protrusions in nocodazole-treated cells and nonaxonal protrusions in control and nocodazole-treated cells. Control nascent axons were not analysed as they do not retract. Bars show mean and standard deviation. Nocodazole nascent axons: $n = 14$ protrusions from 14 cells from three experiments; mean = 65.17 min, s.d. = 20.33. Control nonaxons: $n = 40$ protrusions from 10 cells from three experiments; mean = 17.15 min, s.d. = 8.804. Nocodazole nonaxons: $n = 41$ protrusions from 16 cells from three experiments; mean = 16.182 min, s.d. = 7.147. One-way ANOVA with multiple comparisons: $P < 0.0001$; nocodazole nascent axon vs nocodazole nonaxon $P < 0.0001$; control nonaxon vs nocodazole nonaxon $P = 0.9132$.

D Graph showing length (μm) of nascent axon-like protrusions and nonaxonal protrusions in control and nocodazole-treated cells. Bars show mean and standard deviation. Control nascent axons were analysed after 60 min: $n = 18$ protrusions from 18 cells; mean = 14.092 μm, s.d. = 5.149. Nocodazole nascent axons: $n = 14$ protrusions from 14 cells; mean = 9.596 μm, s.d. = 2.371. Control nonaxons: $n = 40$ protrusions from 10 cells; mean = 5.802 μm, s.d. = 2.553. Nocodazole nonaxons: $n = 41$ protrusion from 16 cells; mean = 4.407 μm, s.d. = 2.024. One-way ANOVA with multiple comparisons: $P < 0.0001$; control nascent axon vs nocodazole nascent axon $P = 0.0002$; control nascent axon vs control nonaxon $P < 0.0001$; nocodazole nascent axon vs nocodazole nonaxon $P < 0.0001$; control nonaxon vs. nocodazole nonaxon $P = 0.1485$.

E Graph showing width (μm) at the base of nascent axon-like protrusions and nonaxonal protrusions in control and nocodazole-treated cells. Bars show mean and standard deviation. Control nascent axons: $n = 19$ protrusions from 19 cells from six experiments; mean = 7.806 μm, s.d. = 3.163. Nocodazole nascent axons: $n = 14$ protrusions from 14 cells from three experiments; mean = 4.188 μm, s.d. = 1.637. Control nonaxons from three experiments: $n = 40$ protrusions from 10 cells; mean = 1.061 μm, s.d. = 0.652. Nocodazole nonaxons from three experiments: $n = 41$ protrusions from 16 cells; mean = 0.984 μm, s.d. = 0.757. One-way ANOVA with multiple comparisons: $P < 0.0001$; control nascent axon vs nocodazole nascent axon $P < 0.0001$; control nascent axon vs control nonaxon $P < 0.0001$; nocodazole nascent axon vs nocodazole nonaxon $P < 0.0001$; control nonaxon vs nocodazole nonaxon $P = 0.9958$.

F Plots showing nascent axon position on the soma of control cells relative to the cell centroid at 0,0 for dorsal and transverse views ($n = 19$ cells from six experiments). Numbers show total count of axons in each quadrant.

G Plots showing nascent axon position on the soma of nocodazole-treated cells relative to the cell centroid at 0,0 for dorsal and transverse views ($n = 14$ cells from three experiments). Numbers show total count of axons in each quadrant.

H Plots showing the position of nonaxonal protrusions on the soma of control cells relative to the cell centroid at 0,0 for dorsal and transverse views ($n = 40$ protrusions from 10 cells from three experiments). Numbers show total count of protrusions in each quadrant.

I Plots showing the position of nonaxonal protrusions on the soma of nocodazole-treated cells relative to the cell centroid at 0,0 for dorsal and transverse views ($n = 41$ protrusions from 16 cells from three experiments). Numbers show total count of protrusions originating in each quadrant.

J Graph showing the ratio of kif5c560-YFP fluorescence intensity in the soma compared to the axon with or without nocodazole. Bars show mean and standard deviation. Control: $n = 6$ cells from four experiments; mean = 1.81, s.d. = 1.59. Nocodazole: $n = 7$ cells from four experiments; mean = 0.75, s.d. = 0.19.

K Image sequence from confocal time lapse of a neuron that extends a nascent axon during nocodazole treatment labelled with a membrane marker and kif5c560-YFP before (−10 m) and during (0 h to 144 min) nocodazole treatment. A nascent axon-like protrusion is extended during nocodazole treatment (92 m to 144 m), but kif5c560-YFP does not accumulate there. Images are transverse reconstructions from confocal z-stacks.

Data information: All scale bars = 10 μm. ***$P < 0.001$. ****$P < 0.0001$.
Source data are available online for this figure.

▶

 © 2022 The Authors

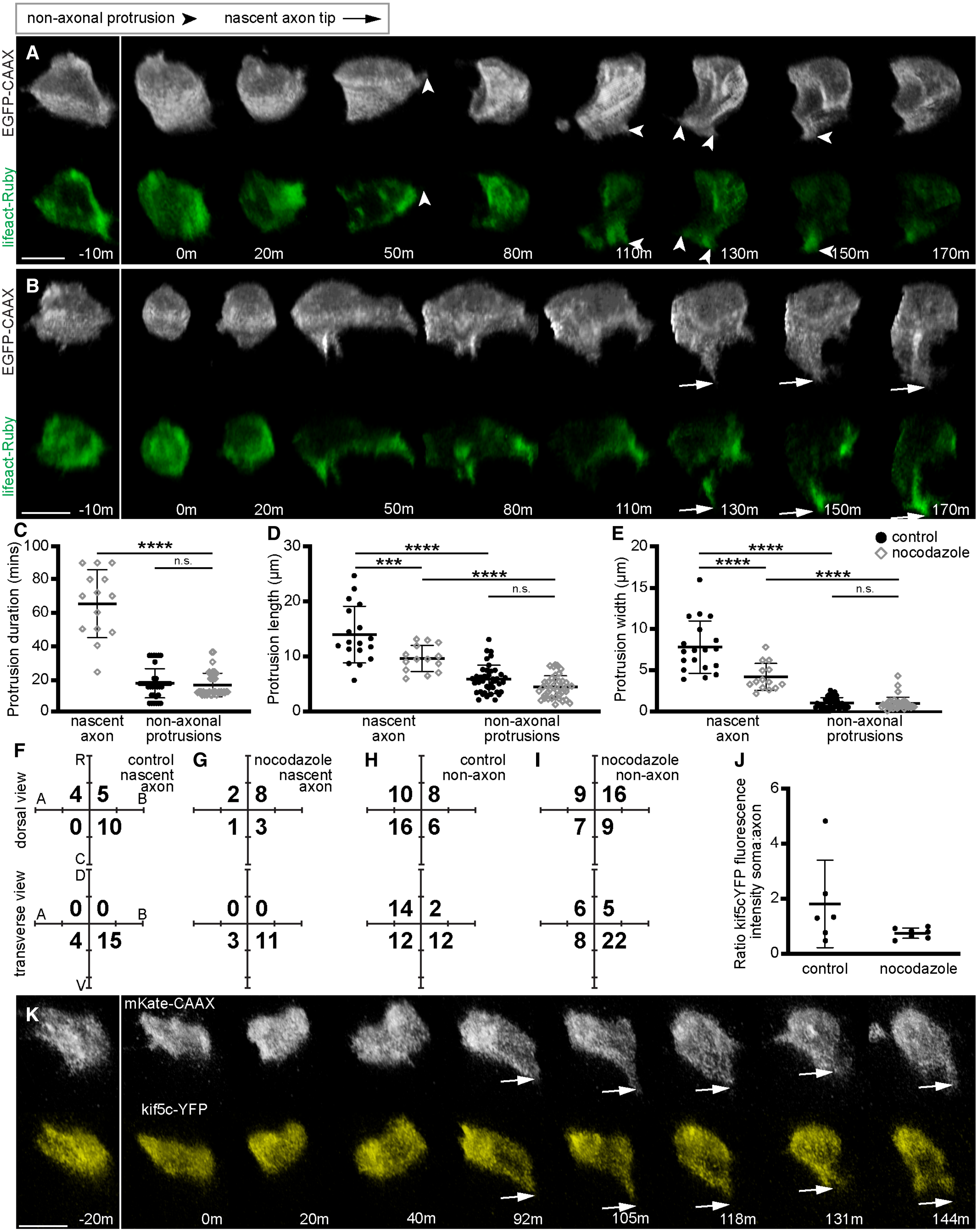
non-axonal protrusion
nascent axon tip
A
EGFP-CAAX
lifeact-Ruby
-10m
0m
20m
50m
80m
110m
130m
150m
170m
B
EGFP-CAAX
lifeact-Ruby
C
Protrusion duration (mins)
nascent axon
non-axonal protrusions
****
n.s.
D
Protrusion length (µm)
***
E
Protrusion width (µm)
control
nocodazole
F
dorsal view
transverse view
control nascent axon
R
C
D
V
A
B
G
nocodazole nascent axon
H
control non-axon
I
nocodazole non-axon
J
Ratio kif5cYFP fluorescence intensity soma:axon
K
mKate-CAAX
kif5c-YFP
-20m
40m
92m
105m
118m
131m
144m

Figure 5.

Laminin provides a basal cue for axon initiation

Finally, we investigated external factors that may be responsible for directing nascent axon formation. As axon initiation occurs from the baso-ventral side of the soma, we hypothesised that the nascent axon is extended close to the neuroepithelial basal surface. We investigated this by randomly labelling cells with a membrane marker in a utrophin reporter line that marks the neuroepithelial basal surface (Tg(actb1:*utr-mCherry*); Fig 6A). We used time-lapse imaging with Airyscan acquisition and processing to observe the position of the neuronal soma, the base of the nascent axon and the tip of the nascent axon protrusion with respect to the neuroepithelial basal surface at the time of axon initiation (Fig 6C). Fluorescence intensity analysis showed that all three regions of the neuron were within a few microns of the basal edge of the neuroepithelium (Fig 6C and E).

One neuronal subtype in the zebrafish spinal cord derives from the division of a nonapical progenitor (Vsx1 progenitors) close to the basal surface of the neural tube. The Vsx1 progenitors are rare and undergo final mitosis producing two neurons that rapidly extend axons (McIntosh *et al*, 2017). We observed two cases of nonapical progenitor divisions in which mitotic cleavage resulted in one daughter adjacent to the basal surface and the other apparently not in contact with the basal surface (Fig 6B). Interestingly, the daughter without contact with the basal surface initiated its axon from the ventral but not basal side of the soma while the daughter adjacent to the basal surface initiated its axon baso-ventrally along the basal surface as expected (Fig 6B: 0 min; n = 2/2 divisions).

This led us to hypothesise that the basal surface of the neural tube may provide a directional cue for nascent axon specification in spinal cord neurons. The extracellular matrix protein Laminin, a component of the basal lamina, can influence neuronal polarity and promote axon outgrowth *in vitro* (Esch *et al*, 1999), and orient axon outgrowth in zebrafish retinal ganglion cells *in vivo* (Randlett *et al*, 2011), so it is a good candidate to provide a directional cue. We used utrophin reporter line embryos injected with LamininC1 morpholino (lamMO; Parsons *et al*, 2002) to ask whether the presence of laminin influenced the site of axon initiation. Fluorescence intensity analysis showed that in the Laminin-depleted embryos the site of axon initiation and the tip of the nascent axon protrusion were significantly further away from the edge of the basal lamina than in control embryos (Fig 6D and E). The position of the soma was not

Figure 6. Laminin provides a basal cue for axon initiation.

A Transverse section from a confocal z-stack showing the whole neural tube of a utr-mCherry embryo, showing localisation to the basal surface.

B Transverse sections from a confocal z-stack of a nonapical progenitor labelled with a membrane marker in a utr-mCherry embryo to identify the basal surface of the spinal cord. Image sequence from confocal time lapse shows the nonapical progenitor (−44 m) undergoing mitosis to produce two neurons (−22 m), of which one is not in contact with the basal surface. Both neurons extend nascent axons (0 m).

C Transverse sections from a confocal z-stack of a neuron at the time of nascent axon initiation labelled with a membrane marker in a utr-mCherry embryo to identify the basal surface of the spinal cord. Three different sections show the middle of the soma, the axon initiation site and the axon tip. Green and magenta peaks in graphs show relative positions of cell membrane and basal surface, respectively.

D Transverse sections from confocal z-stacks of a neuron at the time of nascent axon initiation labelled with a membrane marker in a utr-mCherry embryo injected with lamMO. Three different sections show the middle of the soma, the axon initiation site and the axon tip. Green and magenta peaks in graphs show relative positions of cell membrane and basal surface, respectively.

E Graphs showing the distance (µm) between the basal edge of the soma, the axon initiation site or the axon tip and the basal surface of the spinal cord. Measurements were performed by measuring between basal-most green and magenta peaks in graphs of relative grey values (C, D). Bars show mean and standard deviation. WT: n = 5 cells from one experiment; lamMO: n = 11 cells from two experiments. Soma: WT mean = 2.007 µm, s.d. = 1.055; lamMO mean = 1.871 µm, s.d. = 1.830. Student's two-tailed test, P = 0.880. Axon initiation site: WT mean = 1.366 µm, s.d. = 3.13; lamMO mean = 2.353 µm, s.d. = 2.125. Student's two-tailed test, P = 0.468. Axon tip: WT mean = 0.637 µm, s.d. = 1.078; lamMO mean = 2.903 µm, s.d. = 2.0433. Student's two-tailed test, P = 0.037.

F Image sequence from confocal time lapse shows a neuron in a *Sly*$^{-/-}$ embryo labelled with membrane and centrosome markers before (−40 m), during (0) and after axon initiation (20 m). Images are transverse reconstructions from confocal z-stacks.

G Plots showing axon position on the soma relative to the cell centroid at 0,0 for dorsal and transverse views in *Sly*$^{-/-}$ embryos (n = 18 cells from three experiments). Axon position is not random (dorsal view $P < 0.001$, mean = 98.8°; transverse view $P < 0.001$, mean = 161.1°; Moore's modification of the Rayleigh's test).

H Plots showing merge of WT and *Sly*$^{-/-}$ axon positions on the cell body relative to cell centroid at 0,0 for dorsal and transverse views. Axon positions in WT and *Sly*$^{-/-}$ are not significantly different in dorsal view ($0.2 < P < 0.5$) but are in the transverse view ($P < 0.001$; Batschelet's alternative to the Hotelling test). WT: n = 86 cells from eight experiments; *Sly*$^{-/-}$: n = 18 cells from three experiments.

I Graph showing the distance between centrosome and base of axon at time of axon initiation in WT and *Sly*$^{-/-}$ embryos. Bars show mean and standard deviation. WT: n = 26 cells from three experiments, mean = 10.13 µm, s.d. = 3.35. *Sly*$^{-/-}$: n = 15 cells from two experiments, mean = 12.41 µm, s.d. = 3.281. One-way ANOVA, P = 0.123.

J Plots showing centrosome position relative to the cell centroid at 0,0 for dorsal and transverse views in *Sly*$^{-/-}$ embryos (n = 15 cells from two experiments). Centrosome position is not random (dorsal view $P < 0.001$, mean = −129.0°; transverse view $0.001 < P < 0.005$, mean = −57.0°; Moore's modification of the Rayleigh's test).

K Plots showing merge of WT and *Sly*$^{-/-}$ centrosome positions on the cell body relative to cell centroid at 0,0 for dorsal and transverse views. Centrosome positions are not significantly different between WT and *Sly*$^{-/-}$ (dorsal view $P > 0.2$, transverse view $0.1 < P < 0.2$; Batschelet's alternative to the Hotelling test). WT: n = 26 cells from three experiments; *Sly*$^{-/-}$: n = 15 cells from two experiments.

L Plots showing the positions of the centrosome and base of the axon in *Sly*$^{-/-}$ embryos at the time of axon initiation relative to the cell centroid at 0,0 for dorsal and transverse views (n = 15 cells from two experiments). Left-hand plots: centrosome position is not random (dorsal view $P < 0.001$, mean = −129.0°; transverse view $P < 0.001$, mean = −57.0) and axon position is not random (dorsal view $P < 0.001$, mean = 95.1°; transverse view $P < 0.001$, mean = 168.8°, Moore's modification of the Rayleigh's test). Centrosome and axon positions are significantly different (dorsal view $0.001 > P$; transverse view $0.001 > P$; Moore's test for paired data). Right-hand plots: vectors connecting centrosome and nascent axon from the same cell are not random (dorsal view $P < 0.001$, mean = 66.1°; transverse view $P < 0.001$, mean = 150.9°).

Data information: All scale bars = 5 µm. *$P < 0.05$.
Source data are available online for this figure.

 © 2022 The Authors

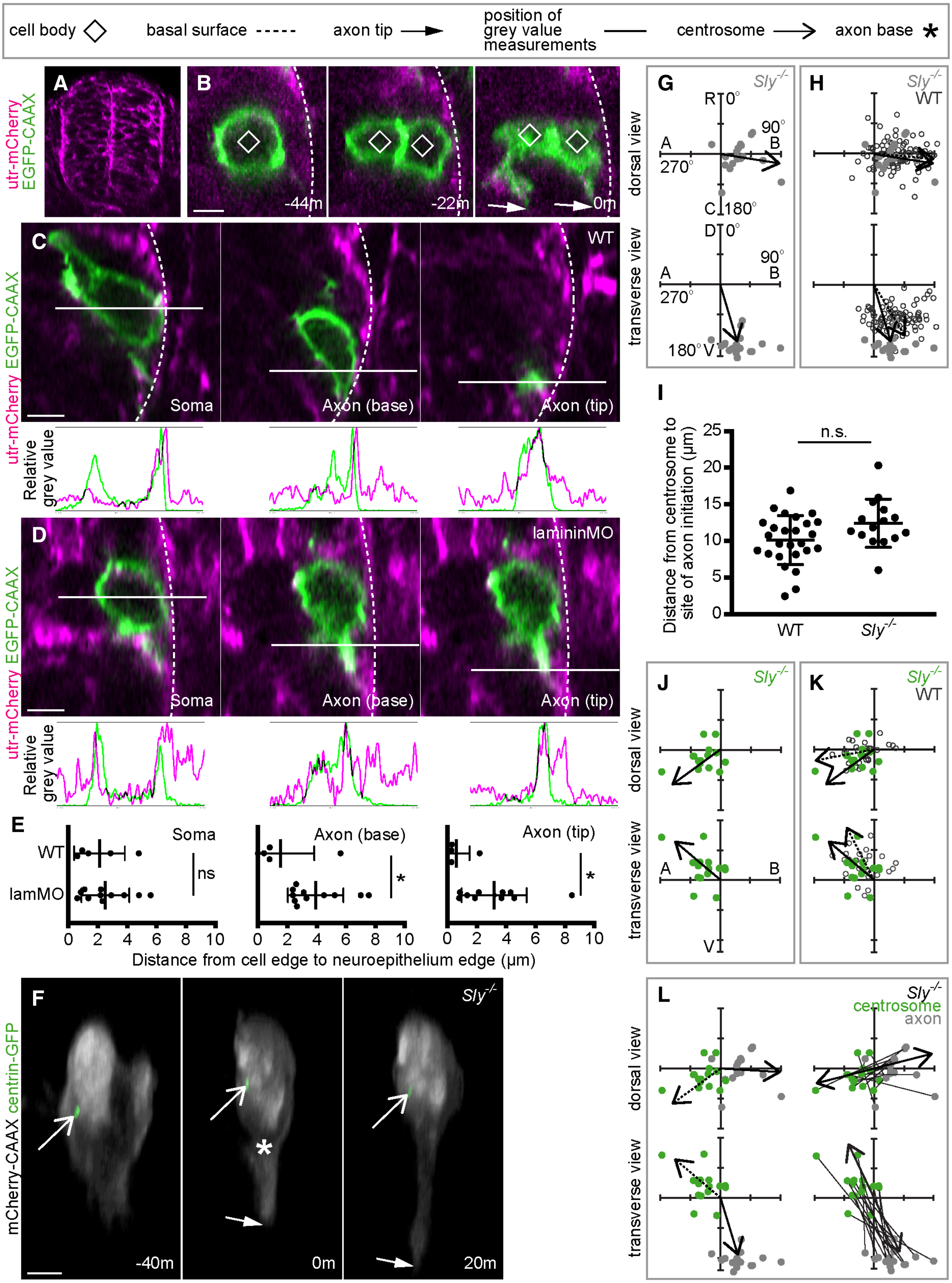
cell body
basal surface
axon tip
position of grey value measurements
centrosome
axon base
A
B
utr-mCherry EGFP-CAAX
-44m
-22m
0m
C
WT
Soma
Axon (base)
Axon (tip)
Relative grey value
D
lamininMO
E
Soma
Axon (base)
Axon (tip)
WT
lamMO
ns
Distance from cell edge to neuroepithelium edge (µm)
F
Sly-/-
mCherry-CAAX centrin-GFP
-40m
0m
20m
G
H
Sly-/-
WT
dorsal view
transverse view
R 0°
90°
A
B
270°
C 180°
D 0°
180° V
I
Distance from centrosome to site of axon initiation (µm)
n.s.
WT
Sly-/-
J
K
L
centrosome
axon
V

Figure 6.

affected, indicating that this is not due to incorrect positioning of the neuron (Fig 6E).

To examine the role of Laminin in neuronal polarity more closely we analysed the site of axon initiation in the *Sly/LamC1* zebrafish mutant line, which has no detectable Laminin expression in the basal lamina at this embryonic stage (Fig 6F, compare to Fig 1C; Movie EV9). There was no difference in the position of nascent axon initiation between controls and $Sly^{-/-}$ embryos when analysed from the dorsal perspective, but analysis from the transverse perspective showed the nascent axon was less basal and more ventral compared with controls (Fig 6F: 0 min, G, and H). LamMO-injected embryos had a similar but more severe phenotype than $Sly^{-/-}$ embryos. The basal bias of the site of the nascent axon was lost in cells in lamMO-injected embryos and the ventral bias was increased compared with controls (Appendix Fig S10A: 0 m, B, and C). These results suggest that Laminin promotes the basal positioning of the nascent axon formation site in zebrafish spinal cord neurons.

We next assessed the position of the centrosome at the time of axon initiation in Laminin-deficient embryos. As the position of the centrosome tends to be on the opposite side of the cell to the nascent axon position in wildtype cells (Fig 3), we hypothesised that the change in the position of the nascent axon observed in Laminin-deficient embryos may be mirrored by a change in centrosome position. However, there was no difference in the position of the centrosome in $Sly^{-/-}$ embryos (Fig 6J and K; Movie EV12), in the distance between the centrosome and the site of nascent axon in $Sly^{-/-}$ or lamMO-injected embryos compared with controls (Fig 6I; Appendix Fig S10D), or in the centrosome-axon axes between $Sly^{-/-}$ or lamMO-injected embryos and controls (Fig 6L; Appendix Fig S10G), although centrosome position is more ventral in lamMO-injected embryos than controls (Appendix Fig S10E and F). Overall, although the centrosome tends to be opposite the nascent axon site in wildtype, changes in axon position resulting from Laminin depletion are not accompanied by significant alterations in centrosome position. This further supports the view that centrosome location is not critical for the location of the nascent axon.

Discussion

We investigated the earliest steps in axon formation in spinal projection neurons *in vivo*. Our key findings are:

i an accumulation of F-actin is an early molecular indicator of axon initiation, precedes the generation of a stable nascent axonal protrusion and precedes microtubule accumulation.
ii nocodazole-treated neurons, with no detectable microtubules or Kif5c560 enrichment, are still able to generate a nascent axon, albeit less dilated and less stable.
iii axon initiation is extremely stereotyped across different spinal neuron subtypes irrespective of subsequent axon guidance.
iv MTOCs, the centrosome and Golgi apparatus, are located on the opposite side of the cell to the site of axon initiation but move to the base of the axon during later axon growth.
v Laminin is not required for axon initiation or early growth but is a positional cue for axon initiation.

The polarisation of individual neurons into axonal and dendritic compartments is critical for correct nervous system development. The mechanisms that may define axon initiation have been studied for several decades, but many of these previous works are compromised by studying this process in neurons growing *in vitro* that lack the complex 3-dimensional architecture and molecular environment of *in vivo* settings. In fact, a recent study using 3-dimensional gel matrices shows that 3-dimensionality significantly enhances axon formation *in vitro* (Santos *et al*, 2020). Additionally, some *in vitro* studies may describe the repolarisation of neurons that had previously polarised *in vivo* rather than neurons polarising for the first time (reviewed in Barnes & Polleux, 2009). Further, axon initiation *in vitro* is the differentiation of a pre-existing neurite to become an axon (Dotti *et al*, 1988), making it difficult to differentiate between axon initiation and axonal growth (Jiang & Rao, 2005; Barnes & Polleux, 2009). For some existing models of neuronal differentiation *in vivo*, it can be difficult to disentangle axon initiation from neurite growth and neuronal migration (Barnes & Polleux, 2009). Our study of spinal neurons *in vivo* overcomes these reservations. The cell bodies of neurons in the zebrafish spinal cord move to the basal surface of the neuroepithelium before delaminating from the apical surface and remain established at the basal surface for several hours before axon initiation (Hadjivasiliou *et al*, 2019), thus the remodelling of cell polarity in this system is not complicated by polarity changes related to cell migration. Axon formation occurs directly from the cell body, so can be clearly identified and separated from axon growth, and we find that it occurs from a stereotyped position in all of the spinal neuron subtypes that we investigated, independently of the subsequent direction of axon projection (except for Rohon-Beard neurons, which elaborate three axons). As such, the zebrafish embryonic spinal cord provides a complex *in vivo* system where we can definitively separate axon initiation from both axonal growth and neuronal migration. We define the first phase of axon initiation as the nascent axon—this is a large, wide and persistent protrusion that is beginning to take on the characteristics of an axon and would normally become an axon when stabilised by microtubules. Once formed the growth of this nascent axon pauses before the extension is reinitiated.

Our principal finding is that microtubules are neither enriched in nor required for nascent axon establishment. The earliest indication of axon initiation is a biased accumulation of F-actin in the baso-ventral quadrant of the neuron cell body. This first coincides with unstable protrusions from the baso-ventral soma and then with a stable protrusion that we term the nascent axon (Fig 4). Although microtubules rapidly invade the nascent axon, we find nascent axons can still be formed in the absence of microtubules (Fig 5). Thus, nascent axon formation from spinal neurons *in vivo* is an F-actin-based protrusion that forms directly from a specific location on the cell body. Although nascent axons form in the absence of microtubules, they are less persistent than normal axons (but can last for up to 90 min) and have a smaller calibre at their proximal end. Microtubules therefore probably add stability and girth to the nascent axon. The retraction of nascent axons that existed before nocodazole treatment (6/7 cells) further suggests microtubules are important for maintaining nascent axons, in addition to their previously suggested role in axon maintenance and growth (Letourneau & Ressler, 1984; Hahn *et al*, 2019). We also observe that fewer neurons generate a nascent axon in the imaging period in nocodazole neurons compared with control cells. We do not know the reason for this, but there are several possibilities. Microtubules may

© 2022 The Authors

increase the likelihood or speed with which focal accumulations of F-actin can initiate a nascent axon. Alternatively, it could be that elements of the local cellular environment that are required for, or enhance, nascent axon initiation in the marginal zone are disrupted. The neuroepithelial scaffold itself is likely to be altered as neuroepithelial cells in the nocodazole-treated embryos will also lack microtubules, and we have previously shown that microtubules are required for correct apico-basal polarisation of neuroepithelial cells (Buckley *et al*, 2013).

Our work shows that nascent axon formation can occur in the absence of any observable microtubules. This is in contrast to the generally held view that actin and microtubule dynamics work together and are both required for axon formation (Sakakibara *et al*, 2013; Pacheco & Gallo, 2016) but is in agreement with previous work on neurite initiation in sympathetic and hippocampal neurons in culture, which observed that neurites (Smith, 1994b) or focal filopodial protrusions (Zhang *et al*, 2016) could be initiated in the presence of nocodazole. The generation of a single stereotyped axonal protrusion from spinal neurons *in vivo* is very different from the axon selection process from multiple random neurites seen in the *in vitro* neuronal polarisation models (e.g. Dotti *et al*, 1988) and reviewed in (Barnes & Polleux, 2009), and this very likely reflects the difference in complexity of environmental cues in these two cases. The very stereotyped location of nascent axon formation from the baso-ventral quadrant of spinal neurons suggests this process is strongly influenced by local environmental cues in the early neural tube and we uncover that one of those cues is Laminin (Fig 6; Appendix Fig S10), an extracellular protein abundant at the basal surface of the neural tube. Laminin may play a common role in the differentiation of early-born neurons as it has previously been shown to influence retinal ganglion cell axon initiation in the retina (Randlett *et al*, 2011) and axonal growth in the primary sensory Rohon-Beard neurons in zebrafish neural tube (Andersen & Halloran, 2012).

Although dilated nascent axons can be produced and maintained for over an hour in the absence of microtubules, they are nonetheless less stable and less dilated than controls and eventually tend to retract, demonstrating a probable requirement for microtubules to stabilise and increase dilation of this protrusion. Perhaps in this respect microtubules cooperate with dynamic actin in similar ways as they do to facilitate turning in neuronal growth cones (reviewed in Geraldo & Gordon-Weeks, 2009) and to promote axon specification (Bradke & Dotti, 1999; Geraldo *et al*, 2008; Witte *et al*, 2008; Zhai *et al*, 2017) and neurite initiation *in vitro* (Dent *et al*, 2007; Flynn *et al*, 2012). Increased numbers of microtubules and enriched microtubule plus-ends could play an important role in anterograde transport (reviewed in Schelski & Bradke, 2017). However, in spinal neurons *in vivo* we see few EB3-labelled growing microtubule plus-ends in both preaxonal protrusions and the nascent axon, illustrating that there are few growing microtubules until axonal growth commences. Nonetheless, it is possible that only a few stable microtubules are required for the next steps in axon differentiation.

Consistent with our observation that microtubules are not required for nascent axon formation, we also show neither the centrosome nor the Golgi complex is close to the site of axon initiation in spinal neurons. Several previous studies have suggested the centrosome and Golgi complex are close to the base of the neurite that becomes the axon *in vitro* (Zmuda & Rivas, 1998; de Anda *et al*, 2005). Centrosome and Golgi complex proximity is associated with the development of a neurite into an axon in multipolar embryonic mouse neocortical neurons *in vivo* (de Anda *et al*, 2010), although it seems that the centrosome translocates to the base of the leading process irrespective of whether it is the axon or leading migratory process (Sakakibara *et al*, 2014). Our results support previous observations that show centrosome proximity is not required for axon initiation (Dotti & Banker, 1991; Zolessi *et al*, 2006; Distel *et al*, 2010; Gärtner *et al*, 2012). There are several potential explanations for discrepancies between these findings. There may be innate differences in cytoskeletal organisation between different neuronal subtypes or species; differences in substrate properties may alter cytoskeletal organisation, as has been shown for migrating cells (Pouthas *et al*, 2008); or alternatively, studies showing MTOCs close to the base of the axon may be looking after the phase of axon initiation. This is supported by our observation that both the centrosome and Golgi complex move close to the base of the axon during pathfinding and a previous study showing that the centrosome is not close to the site of axon initiation in zebrafish retinal ganglion cells, in which axon initiation can also be easily identified (Zolessi *et al*, 2006). The centrosome has previously been described as being associated with peripheral axon formation in Rohon-Beard neurons and to be important for its growth (Andersen & Halloran, 2012), but we find that the centrosome is not close to the base of the axon at the time of initiation of any Rohon-Beard axon, including the peripheral axon. Combining these findings and others (Stiess *et al*, 2010) suggests that centrosome proximity is not required for axon initiation or axon growth, although we cannot rule out that it is associated with the subsequent stabilisation of a nascent axon and transition to a growing axon.

Although not close to the site of axon initiation the centrosome is not positioned randomly in spinal neurons; instead, it is consistently opposite the site of axon initiation. As the centrosome is situated apically while the new neuron is still attached to the apical surface and is retracted into the neuronal cell body upon delamination, it may be that its medial position in the soma at axon initiation is simply related to the location of the retracting apical process. We found no evidence that apical abscission is required for apical process retraction in the zebrafish spinal cord. This is in contrast to chick and mouse but in agreement with some observations in the zebrafish retina (Zolessi *et al*, 2006; Das & Storey, 2014; Lepanto *et al*, 2016). Interestingly, modelling has shown that stochastic microtubule dynamics can lead to stabilisation of the longest microtubules (Seetapun & Odde, 2010), suggesting a method by which the distant centrosome may stabilise the nascent axon on the opposite side of the cell where only the longest microtubules can reach.

The position of the nascent axon is influenced by the extracellular matrix protein Laminin at the basal surface of the neural tube, as the loss of Laminin leads to the loss of the basal bias to the nascent axon position. In the retina, Laminin stabilises newly initiated axons and promotes axonal growth (Randlett *et al*, 2011). The neurons that we observed were among the earliest that differentiated, meaning that they were almost always already adjacent to the Laminin-rich basal surface when extending an axon. It would be interesting to compare this with later-born neurons, which would have earlier-born neurons between them and the basal surface. Nonetheless, we show that axon initiation, stabilisation and growth can occur

© 2022 The Authors

robustly in the spinal cord without Laminin. Although we were unable to find evidence of a dorso-ventrally oriented cue that was required for ventral directional bias of the nascent axon, we cannot rule out that one exists. An alternative to a molecular cue that directs the ventral bias of the nascent axon could be the overall physical architecture of the cells in the early neural tube. All neuroepithelial cells and neurons have a curved morphology in transverse sections, with the lateral poles of both neuroepithelial progenitors and neurons curving ventrally as they approach the basal surface (see Fig 1B, transverse inserts). It seems possible this morphological organisation of cells could provide a 3-dimensional physical substrate or orientation that encourages ventral growth of nascent axon protrusions.

Materials and Methods

All animal procedures were performed according to the UK Animal (Scientific Procedures) Act 1986 and carried out under Home Office Project Licence number PPL P70880F4C, which was subject to local AWERB Committee review and UK Home Office Approval. Wildtype (WT; AB/Tuebingen), transgenic (Tg(actb2:arl13b-GFP)), ZFIN ID: ZDB-ALT-100721-1, (Borovina *et al*, 2010; Tg(actb1:*utr-mCherry*); Krens *et al*, 2017) and mutant (*Sly*/*lamC1*, ZFIN ID: ZDB-FISH-150901-23200; Kettleborough *et al*, 2013) zebrafish lines were maintained under standard conditions in a 14/10 h light/dark cycle (Westerfield, 2000). Embryos were raised in aquarium water at 28.5°C.

To observe individual cells, we injected zebrafish embryos at 32–64 cell stage with mRNA encoding fluorescently-tagged proteins: EGFP-CAAX (Kwan *et al*, 2007), mKate-CAAX (Hadjivasiliou *et al*, 2019), H2B-RFP (Megason & Fraser, 2003), lifeact-Ruby (Riedl *et al*, 2008), Kif5c560-YFP (Randlett *et al*, 2011), EB3-GFP (Norden *et al*, 2009), centrin2-EGFP (Distel *et al*, 2010), centrin2-RFP, GM130-EGFP or GM130-RFP (Durdu *et al*, 2014). Occasionally we coinjected mRNA coding for dominant negative Suppressor of Hairless (dnSuH; Wettstein *et al*, 1997) to increase the likelihood of labelled cells differentiating into neurons. WT or Tg(actb1:*utr-mCherry*) embryos were injected at the one-cell stage with 3.4 ng of LamininC1 morpholino (lamMO; 5′-TGTGCCTTTTGCTATTGCGACCTC-3′; Parsons *et al*, 2002) to disrupt laminin expression.

For immunohistochemistry, embryos were dechorionated, anaesthetised with MS-222 (Sigma-Aldrich/Merck, St. Louis, USA) and fixed in 4% PFA overnight at 4°C. Blocking was performed for 2 h at room temperature in appropriate serum. Antibodies were diluted in a blocking solution. Embryos were incubated in primary antibody overnight at 4° C (chick α-GFP, Abcam, Cat# AB13970, Lot# GF305729-1; mouse α- γ-tubulin, Sigma-Aldrich, Cat# T6557, Lot# 066M4858V; rabbit α- α-tubulin, Abcam, Cat# AB233661) and in secondary antibody for 2 h at room temperature (Alexa goat α-chick 488, Life Technologies, Cat# A11039, Lot# 1812246; Alexa goat α-mouse 568, Life Technologies, Cat# A1104, Lot# 1863187; Alexa goat α-rabbit 633, Life Technologies, Cat# A21071, Lot# 558885).

Imaging was performed from 16 hpf. Embryos were dechorionated, mounted in low-melting point agarose (Sigma-Aldrich) and anaesthetised with MS-222 (Sigma-Aldrich/Merck) if required (Alexandre *et al*, 2010). Confocal imaging was performed on a spinning disc confocal (PerkinElmer, Waltham, U.S.A.) or LSM880 laser scanning confocal (Zeiss, Oberkochen, Germany) with or without Airyscan, using a 20× water immersion objective with a numerical aperture of 0.95 or higher. For high-resolution imaging, we used Zeiss Airyscan acquisition and processing. Lightsheet imaging was performed on a Zeiss LightSheet Z.1 microscope using 10x illumination objectives and 20× water immersion detection objectives. If required, nocodazole (5 mg/ml stock in DMSO) was diluted in fish water to a final concentration of 5 μg/ml. Following treatment, nocodazole was washed out of the imaging chamber with fish water.

Images were acquired from the embryo's dorsal surface as 40–100 μm deep z-stacks. For time lapse, stacks were acquired every 2 s to 10 min over 5–15 h depending on the experiment. Images and videos in the manuscript result from maximum projections of z-stacks using ImageJ (Schindelin *et al*, 2012) or 3D reconstructions using Volocity (Perkin Elmer). Surrounding cells were occasionally edited from the field of view using ImageJ or Imaris (Bitplane, Belfast, UK) to more clearly show behaviours of the individual cells under investigation.

Sample sizes (number of cells) were determined by the number of imaged live labelled cells that were at an appropriate stage of development. Analysis was performed on cells for which the relevant structure/organelle could be identified unambiguously. To analyse organelle position with respect to the cell centroid, the field of view was reoriented so the basal surface was to the right. The cell centroid was determined using the ImageJ 3D Object Counter plugin, and the 3D coordinates of the site of axon initiation, centrosome or Golgi complex were determined manually. Trigonometry was used to calculate the distance and angle of each organelle with respect to the cell centroid and this was analysed using Moore's modification of the Rayleigh statistical test. Trigonometry was also used to calculate the distance between two different organelles within the same cell. These positions were analysed using the Moore's test for paired data. Organelle positions in different conditions were compared using Batschelet's alternative to the Hotelling test. Distances between organelles in different conditions were analysed using the Student's unpaired *t*-test except for centrosome-axon distance in $Sly^{-/-}$ and lamMO-injected embryos, which were compared with WT using one-way ANOVA. Change in cilium length over time was measured using ImageJ and analysed using nonlinear regression to compare each slope to 0 (Prism 8, GraphPad, San Diego, USA). Microtubules were tracked using the ImageJ Manual Tracking plugin and images generated by making a maximum z-projection of microtubule tracks. Protrusion length, width and duration were measured using Volocity and compared using one-way ANOVA with multiple comparisons (Prism 8). As the mean duration of nascent axon-like protrusions in nocodazole-treated cells was approximately 60 min the length of nascent axons in control cells were measured after 60 min for comparison. Nonaxonal protrusions were measured in cells in which protrusions could be unambiguously analysed, including cells that did and cells that did not develop nascent axon-like protrusions. Between one and four nonaxonal protrusions were analysed per cell. Fluorescence intensity analysis was performed using the ImageJ Plot Profile plugin. Distances between neuron and basal surface were compared using the Student's unpaired *t*-test. Where statistical tests assumed normality, the data followed a normal distribution.

© 2022 The Authors

Data availability

This study includes no data deposited in external repositories.

Expanded View for this article is available online.

Acknowledgements

R.E.M. and J.C. were supported by the Wellcome Trust (102895/Z/13/Z). S.P. was supported by an MRC studentship. Thanks to Christopher Rookyard for the image in Fig 6A, Corinne Houart's lab for kindly providing the α-tubulin antibody, and to past and present members of the Clarke lab and CDN for constructive feedback and discussion.

Author contributions

Jonathan DW Clarke: Conceptualization; supervision; funding acquisition; validation; writing – original draft; writing – review and editing. **Rachel E Moore:** Conceptualization; formal analysis; supervision; investigation; visualization; methodology; writing – original draft; writing – review and editing. **Sinziana Pop:** Investigation. **Cache Alleyne:** Investigation.

Disclosure and competing interest statement

The authors declare that they have no conflict of interest.

References

Alexandre P, Reugels AM, Barker D, Blanc E, Clarke JD (2010) Neurons derive from the more apical daughter in asymmetric divisions in zebrafish neural tube. *Nat Neurosci* 13: 673 – 679

de Anda FC, Pollarolo G, Da Silva JS, Camoletto PG, Dotti CG (2005) Centrosome localization determines neuronal polarity. *Nature* 436: 704 – 708

de Anda FC, Meletis K, Ge X, Rei D, Tsai LH (2010) Centrosome motility for initial axon formation in the neocortex. *J Neurosci* 30: 10391 – 10406

Andersen EF, Halloran MC (2012) Centrosome movements *in vivo* correlate with specific neurite formation downstream of LIM homeodomain transcription factor activity. *Development* 139: 3590 – 3599

Barnes AP, Polleux F (2009) Establishment of axon-dendrite polarity in developing neurons. *Annu Rev Neurosci* 32: 347 – 381

Bernhardt RR, Chitnis AB, Lindamer L, Kuwada JY (1990) Identification of spinal neurons in the embryonic and larval zebrafish. *J Comp Neurol* 302: 603 – 616

van Beuningen SFB, Will L, Harterink M, Chazeau A, van Battum EY, Frias CP, Franker MAM, Katrukha EA, Stucchi R, Vocking K *et al* (2015) TRIM46 controls neuronal polarity and axon specification by driving the formation of parallel microtubule arrays. *Neuron* 88: 1208 – 1226

Borovina A, Superina S, Voskas D, Ciruna B (2010) Vangl2 directs the posterior tilting and asymmetric localization of motile primary cilia. *Nat Cell Biol* 12: 407 – 412

Bradke F, Dotti CG (1999) The role of local Actin instability in axon formation. *Science* 283: 1931 – 1934

Buckley CE, Ren X, Ward LC, Girdler GC, Araya C, Green MJ, Clark BS, Link BA, Clarke JD (2013) Mirror-symmetric microtubule assembly and cell interactions drive lumen formation in the zebrafish neural rod. *EMBO J* 32: 30 – 44

Chabin-Brion K, Marceiller J, Perez F, Settegrana C, Drechou A, Durand G, Poüs C (2001) The Golgi complex is a microtubule-organizing organelle. *Mol Biol Cell* 12: 2047 – 2060

Conde C, Cáceres A (2009) Microtubule assembly, organization and dynamics in axons and dendrites. *Nat Rev Neurosci* 10: 319 – 332

Das RM, Storey KG (2014) Apical abscission alters cell polarity and dismantles the primary cilium during neurogenesis. *Science* 343: 200 – 204

Dent EW, Kwiatkowski AV, Mebane LM, Philippar U, Barzik M, Rubinson DA, Gupton S, Van Veen JE, Furman C, Zhang J *et al* (2007) Filopodia are required for cortical neurite initiation. *Nat Cell Biol* 9: 1347 – 1359

Distel M, Hocking JC, Volkmann K, Köster RW (2010) The centrosome neither persistently leads migration nor determines the site of axonogenesis in migrating neurons *in vivo*. *J Cell Biol* 191: 875 – 890

Dotti CG, Banker G (1991) Intracellular organization of hippocampal neurons during the development of neuronal polarity. *J Cell Sci Suppl* 15: 75 – 84

Dotti CG, Sullivan CA, Banker GA (1988) The establishment of polarity by hippocampal neurons in culture. *J Neurosci* 8: 1454 – 1468

Durdu S, Iskar M, Revenu C, Schieber N, Kunze A, Bork P, Schwab Y, Gilmour D (2014) Luminal signalling links cell communication to tissue architecture during organogenesis. *Nature* 515: 120 – 124

Esch T, Lemmon V, Banker G (1999) Local presentation of substrate molecules directs axon specification by cultured hippocampal neurons. *J Neurosci* 19: 6417 – 6426

Flynn KC, Hellal F, Neukirchen D, Jacob S, Tahirovic S, Dupraz S, Stern S, Garvalov BK, Gurniak C, Shaw AE *et al* (2012) ADF/cofilin-mediated Actin retrograde flow directs neurite formation in the developing brain. *Neuron* 76: 1091 – 1107

Gallo G, Letourneau PC (1999) Different contributions of microtubule dynamics and transport to the growth of axons and collateral sprouts. *J Neurosci* 19: 3860 – 3873

Gärtner A, Fornasiero EF, Munck S, Vennekens K, Seuntjens E, Huttner WB, Valtorta F, Dotti CG (2012) N-cadherin specifies first asymmetry in developing neurons. *EMBO J* 31: 1893 – 1903

Geraldo S, Khanzada UK, Parsons M, Chilton JK, Gordon-Weeks PR (2008) Targeting of the F-Actin-binding protein drebrin by the microtubule plus-tip protein EB3 is required for neuritogenesis. *Nat Cell Biol* 10: 1181 – 1189

Geraldo S, Gordon-Weeks PR (2009) Cytoskeletal dynamics in growth-cone steering. *J Cell Sci* 122: 3595 – 3604

Hadjivasiliou Z, Moore RE, McIntosh R, Galea GL, Clarke JGW, Alexandre P (2019) Basal protrusions mediate spatiotemporal patterns of spinal neuron differentiation. *Dev Cell* 49: 907 – 919.e10

Hahn I, Voelzmann A, Liew YT, Costa-Gomes B, Prokop A (2019) The model of local axon homeostasis - explaining the role and regulation of microtubule bundles in axon maintenance and pathology. *Neural Dev* 14: 11

Hale ME, Ritter DA, Fetcho JR (2001) A confocal study of spinal interneurons in living larval zebrafish. *J Comp Neurol* 437: 1 – 16

Head J, Lee LL, Field DJ, Lee JC (1985) Equilibrium and rapid kinetic studies on nocodazole-tubulin interaction. *J Biol Chem* 260: 11060 – 11066

Inagaki N, Chihara K, Arimura N, Ménager C, Kawano Y, Matsuo N, Nishimura I, Amano M, Kaibuchi K (2001) CRMP-2 induces axons in cultured hippocampal neurons. *Nat Neurosci* 4: 781 – 782

Jacobson C, Schnapp B, Banker GA (2006) A change in the selective translocation of the Kinesin-1 motor domain marks the initial specification of the axon. *Neuron* 49: 797 – 804

Jiang H, Rao Y (2005) Axon formation: Fate versus growth. *Nat Neurosci* 8: 544 – 546

Jordan MA, Wilson L (1998) Use of drugs to study role of microtubule assembly dynamics in living cells. *Methods Enzymol* 298: 252 – 276

Kettleborough RN, Busch-Nentwich EM, Harvey SA, Dooley CM, de Bruijn E, van Eeden F, Sealy I, White RJ, Herd C, Nijman IJ *et al* (2013) A systematic

© 2022 The Authors

genome-wide analysis of zebrafish protein-coding gene function. *Nature* 496: 494–497

Kishi M, Pan YA, Crump JG, Sanes JR (2005) Mammalian SAD kinases are required for neuronal polarization. *Science* 307: 929–932

Krens SFG, Veldhuis JH, Barone V, Čapek D, Maître JL, Brodland GW, Heisenberg CP (2017) Interstitial fluid osmolarity modulates the action of differential tissue surface tension in progenitor cell segregation during gastrulation. *Development* 144: 1798–1806

Kunda P, Paglini G, Quiroga S, Kosik K, Caceres A (2001) Evidence for the involvement of Tiam1 in axon formation. *J Neurosci* 21: 2361–2372

Kwan KM, Fujimoto E, Grabher C, Mangum BD, Hardy ME, Campbell DS, Parant JM, Yost HJ, Kanki JP, Chien CB (2007) The Tol2kit: A multisite gateway-based construction kit for Tol2 transposon transgenesis constructs. *Dev Dyn* 236: 3088–3099

Lepanto P, Davison C, Casanova G, Badano JL, Zolessi FR (2016) Characterization of primary cilia during the differentiation of retinal ganglion cells in the zebrafish. *Neural Dev* 11: 10

Letourneau PC, Ressler AH (1984) Inhibition of neurite initiation and growth by taxol. *J Cell Biol* 98: 1355–1362

McIntosh R, Norris J, Clarke JD, Alexandre P (2017) Spatial distribution and characterization of non-apical progenitors in the zebrafish embryo central nervous system. *Open Biol* 7: 160312

Megason SG, Fraser SE (2003) Digitizing life at the level of the cell: high-performance laser-scanning microscopy and image analysis for in toto imaging of development. *Mech Dev* 120: 1407–1420

Ménager C, Arimura N, Fukata Y, Kaibuchi K (2004) PIP3 is involved in neuronal polarization and axon formation. *J Neurochem* 89: 109–118

Moritz M, Agard DA (2001) Gamma-tubulin complexes and microtubule nucleation. *Curr Opin Struct* 11: 174–181

Norden C, Young S, Link BA, Harris WA (2009) Actomyosin is the main driver of interkinetic nuclear migration in the retina. *Cell* 138: 1195–1208

Pacheco A, Gallo G (2016) Actin filament-microtubule interactions in axon initiation and branching. *Brain Res Bull* 126: 300–310

Parsons MJ, Pollard SM, Saúde L, Feldman B, Coutinho P, Hirst EM, Stemple DL (2002) Zebrafish mutants identify an essential role for laminins in notochord formation. *Development* 129: 3137–3146

Pouthas F, Girard P, Lecaudey B, Ly TB, Gilmour D, Boulin C, Pepperkok R, Reynaud EG (2008) In migrating cells, the Golgi complex and the position of the centrosome depend on geometrical constraints of the substratum. *J Cell Sci* 121: 2406–2414

Randlett O, Poggi L, Zolessi FR, Harris WA (2011) The oriented emergence of axons from retinal ganglion cells is directed by laminin contact *in vivo*. *Neuron* 70: 266–280

Riedl J, Crevenna AH, Kessenbrock K, Yu JH, Neukirchen D, Bista M, Bradke F, Jenne D, Holak TA, Werb Z *et al* (2008) Lifeact: a versatile marker to visualize F-Actin. *Nat Methods* 5: 605–607

Sakakibara A, Ando R, Sapir T, Tanaka T (2013) Microtubule dynamics in neuronal morphogenesis. *Open Biol* 3: 130061

Sakakibara A, Sato T, Ando R, Nogichi N, Masaoka M, Miyata T (2014) Dynamics of centrosome translocation and microtubule organisation in neocortical neurons during distince modes of polarization. *Cerebl Cortex* 24: 1301–1310

Santos TE, Schaffran B, Broguière N, Meyn L, Zenobi-Wong M, Bradke F (2020) Axon growth of CNS neurons in three dimensions is amoeboid and independent of adhesions. *Cell Rep* 32: 107907

Schelski M, Bradke F (2017) Neuronal polarization: From spatiotemporal signaling to cytoskeletal dynamics. *Mol Cell Neurosci* 84: 11–28

Schindelin J, Arganda-Carreras I, Frise E, Kaynig V, Longair M, Pietzsch T, Preibisch S, Rueden C, Saalfeld S, Schmid B *et al* (2012) Fiji: an open-source platform for biological-image analysis. *Nat Methods* 9: 676–682

Schwamborn JC, Püschel AW (2004) The sequential activity of the GTPases Rap1B and Cdc42 determines neuronal polarity. *Nat Neurosci* 7: 923–929

Seetapun D, Odde DJ (2010) Cell-length-dependent microtubule accumulation during polarization. *Curr Biol* 20: 979–998

Shelly M, Cancedda L, Heilshorn S, Sumbre G, Poo MM (2007) LKB1/STRAD promotes axon initiation during neuronal polarization. *Cell* 129: 565–577

Shi S-H, Jan LY, Jan Y-N (2003) Hippocampal neuronal polarity specified by spatially localized mPar3/mPar6 and PI 3-kinase activity. *Cell* 112: 63–75

Smith CL (1994a) Cytoskeletal movements and substrate interactions during initiation of neurite outgrowth by sympathetic neurons *in vitro*. *J Neurosci* 14: 384–398

Smith CL (1994b) The initiation of neurite outgrowth by sympathetic neurons grown *in vitro* does not depend on assembly of microtubules. *J Cell Biol* 127: 1407–1418

Stiess M, Maghelli N, Kapitein LC, Gomis-Rüth S, Wilch-Bräuninger M, Hoogenraad CC, Tolić-Nørrelykke IM, Bradke F (2010) Axon extension occurs independently of centrosomal microtubule nucleation. *Science* 327: 704–707

Westerfield M (2000) *The zebrafish book: a guide for the laboratory use of zebrafish (Danio rerio)*, 4th edn. Eugene: University of Oregon Press

Wettstein DA, Turner DL, Kintner C (1997) The xenopus homolog of drosophila suppressor of hairless mediates notch signaling during primary neurogenesis. *Development* 124: 693–702

Witte H, Neukirchen D, Bradke F (2008) Microtubule stabilization specifies initial neuronal polarization. *J Cell Biol* 180: 619–632

Zhai B, Meka DP, Scharrenberg R, König T, Schwanke B, Kobler O, Windhorst S, Kreutz MR, Mikhaylova M, Calderon de Anda F (2017) Microtubules modulate F-Actin dynamics during neuronal polarization. *Sci Rep* 7: 9583

Zhang S-X, Duan L-H, Yu X (2016) Actin aggregations mark the site of neurite initiation. *Neurosci Bull* 32: 1–15

Zmuda JF, Rivas RJ (1998) The Golgi apparatus and the centrosome are localized to the sites of newly emerging axons in cerebellar granule neurons *in vitro*. *Cell Motil Cytoskeleton* 41: 18–38

Zolessi FR, Poggi L, Wilkinson CJ, Chien CB, Harris WA (2006) Polarization and orientation of retinal ganglion cells *in vivo*. *Neural Dev* 1: 2

License: This is an open access article under the terms of the Creative Commons Attribution License, which permits use, distribution and reproduction in any medium, provided the original work is properly cited.

Expanded View Figures

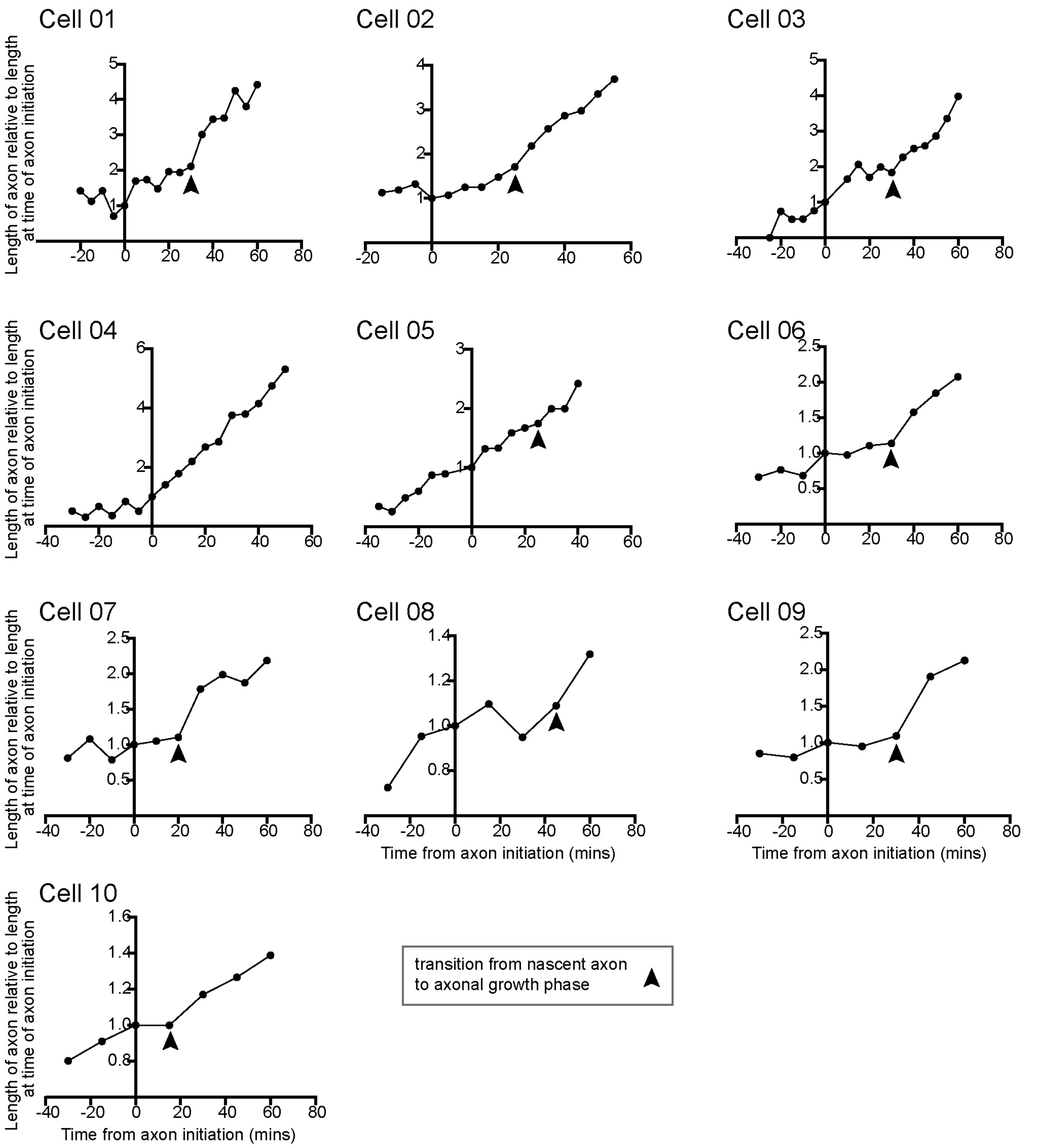

Figure EV1. Axon initiation often has two distinct phases.

Graphs showing the maximum protrusion length from ten cells from four experiments before, during (0 min) and after axon initiation. Length is shown relative to length at the time of axon initiation (0 min). Axon initiation identified by the persistent length and position of a dilated protrusion that later transitions to growing axon. Arrowheads show transition from nascent axon to growth phase.

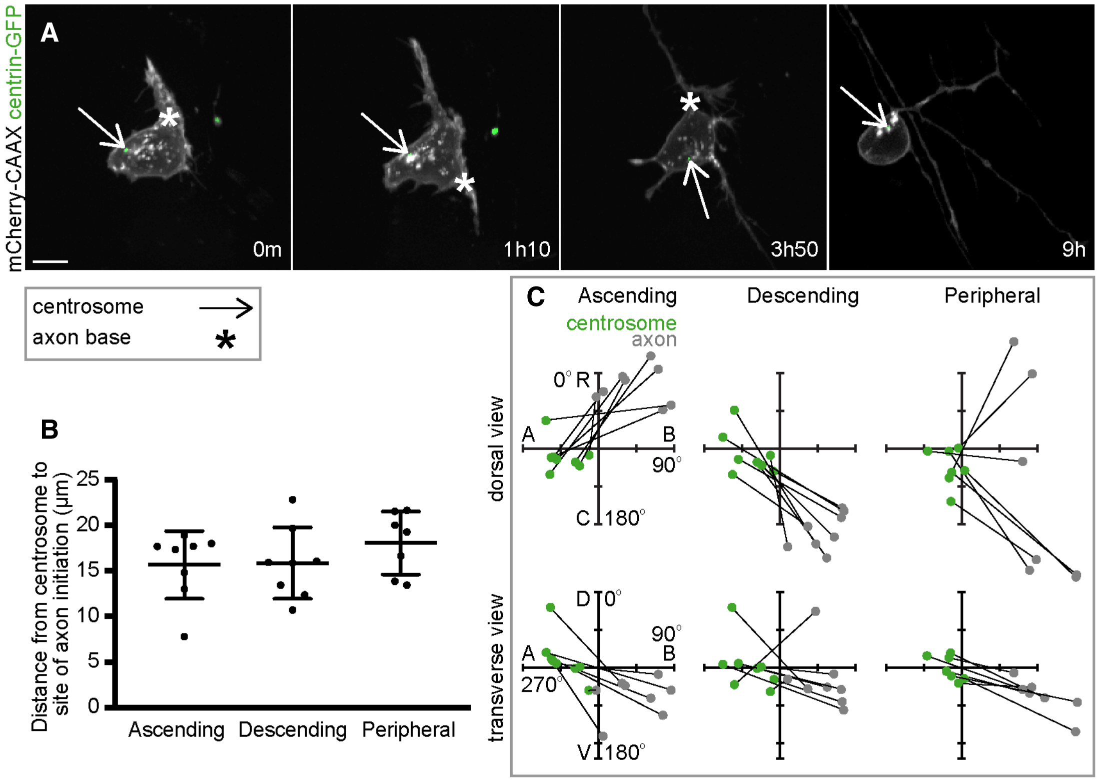

Figure EV2. The centrosome is not close to Rohon-Beard neuron axons during initiation.

A Image sequence from confocal time lapse shows a Rohon-Beard neuron labelled with membrane and centrosome markers during the initiation of the ascending (0 m), descending (1 h10 min) and peripheral axons (3 h 50 min), and during axon pathfinding. The centrosome is located away from the base of each axon but moves close to the peripheral axon during pathfinding. Images are maximum projections from confocal z-stacks. Scale bar = 10 µm.

B Graph showing distance between centrosome and base of the axon at time of initiation of each axon (n = 23 events from 8 cells from three experiments). Bars show mean and standard deviation.

C Plots showing the positions of the centrosome and base of the axon at the time of axon initiation relative to the cell centroid at 0,0 for dorsal and transverse views for ascending (n = 8 cells), descending (n = 8 cells) and peripheral axons (n = 7 cells). Lines connect centrosome and nascent axon from the same cell.

 © 2022 The Authors

APPENDIX

Table of Contents:

Appendix Figure S1. Cell labelling randomly targets all early embryonic neuronal subtypes.
Appendix Figure S2. ɣ-tubulin accumulates at one concentrated point during axon initiation.
Appendix Figure S3. Lifeact-Ruby is enriched in the nascent axon before nascent axon establishment.
Appendix Figure S4. EB3-GFP is not enriched in the nascent axon during nascent axon establishment.
Appendix Figure S5. Lifeact-Ruby is enriched in the nascent axon before EB3-GFP.
Appendix Figure S6. Kif5c560-YFP is enriched in the nascent axon following nascent axon establishment.
Appendix Figure S7. Lifeact-Ruby is enriched in the nascent axon before kif5c650-YFP.
Appendix Figure S8. Lifeact-Ruby is enriched in the nascent axon before EB3-GFP or kif5c560-YFP
Appendix Figure S9. Nocodazole treatment completely disrupts the microtubule array in newborn neurons and neuroepithelial cells.
Appendix Figure S10. LamMO-injected embryo have a similar phenotype to *Sly*$^{-/-}$ embryos.

A

RB	29
DoLA	9
CoPA	5
CoSA	28
CiA	19
CiD	19
CoB	14
VeLD	4
M	1
UCoD	24

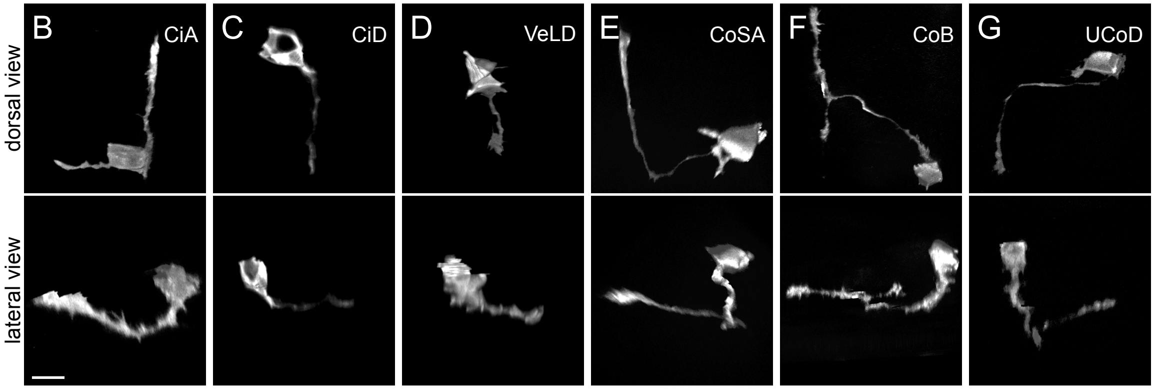

Appendix Figure S1 - Cell labelling randomly targets all early embryonic neuronal subtypes.

A Number of each neuronal subtype labelled.

B-G Examples of CiA (B), CiD (C), VeLD (D), CoSA (E), CoB (F) and CoD (G) neurons and their axon trajectories as dorsal and lateral reconstructions from confocal z-stacks. Scale bar = 10 μm.

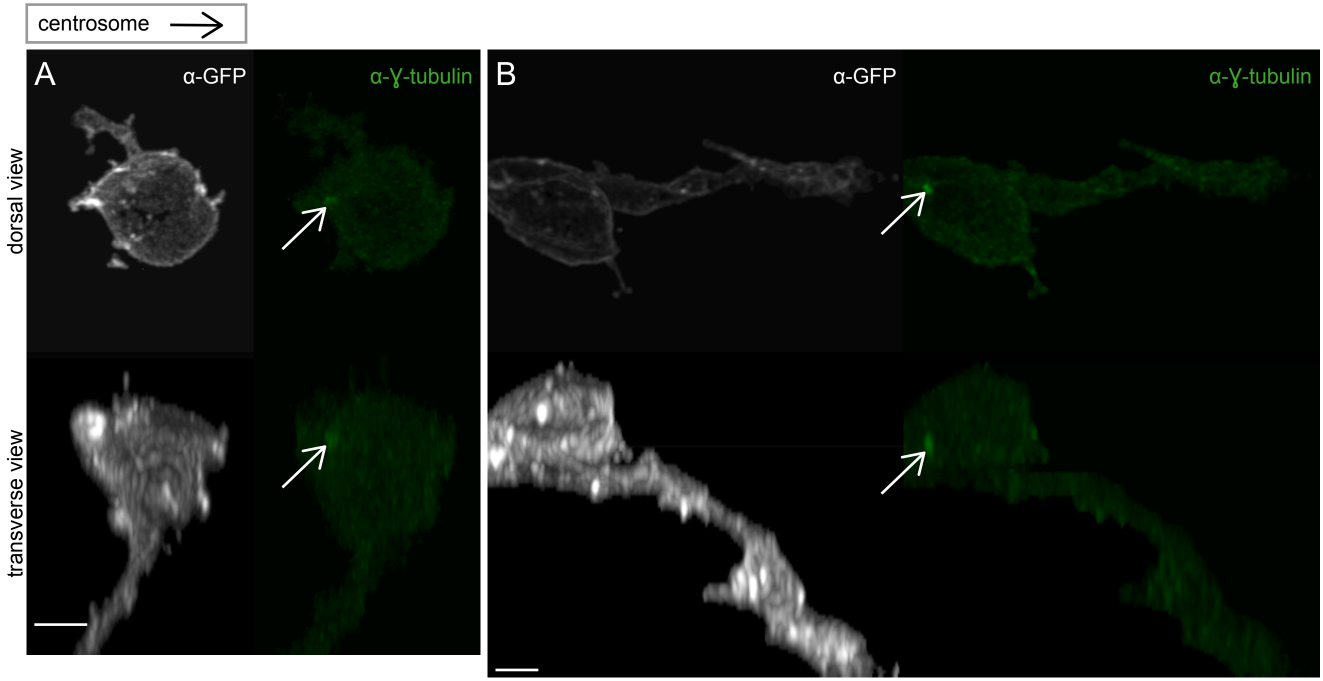

Appendix Figure S2 - γ-tubulin accumulates at one concentrated point during axon initiation.

A, B Two examples of neurons labelled with membrane label EGFP-CAAX in embryos processed for immunohistochemistry against GFP and γ-tubulin. γ-tubulin accumulations appear to correspond with the centrosome. Dorsal views are maximum projections from confocal z-stacks. Transverse views are 3D reconstructions from confocal z-stacks. Scale bars = 5 μm.

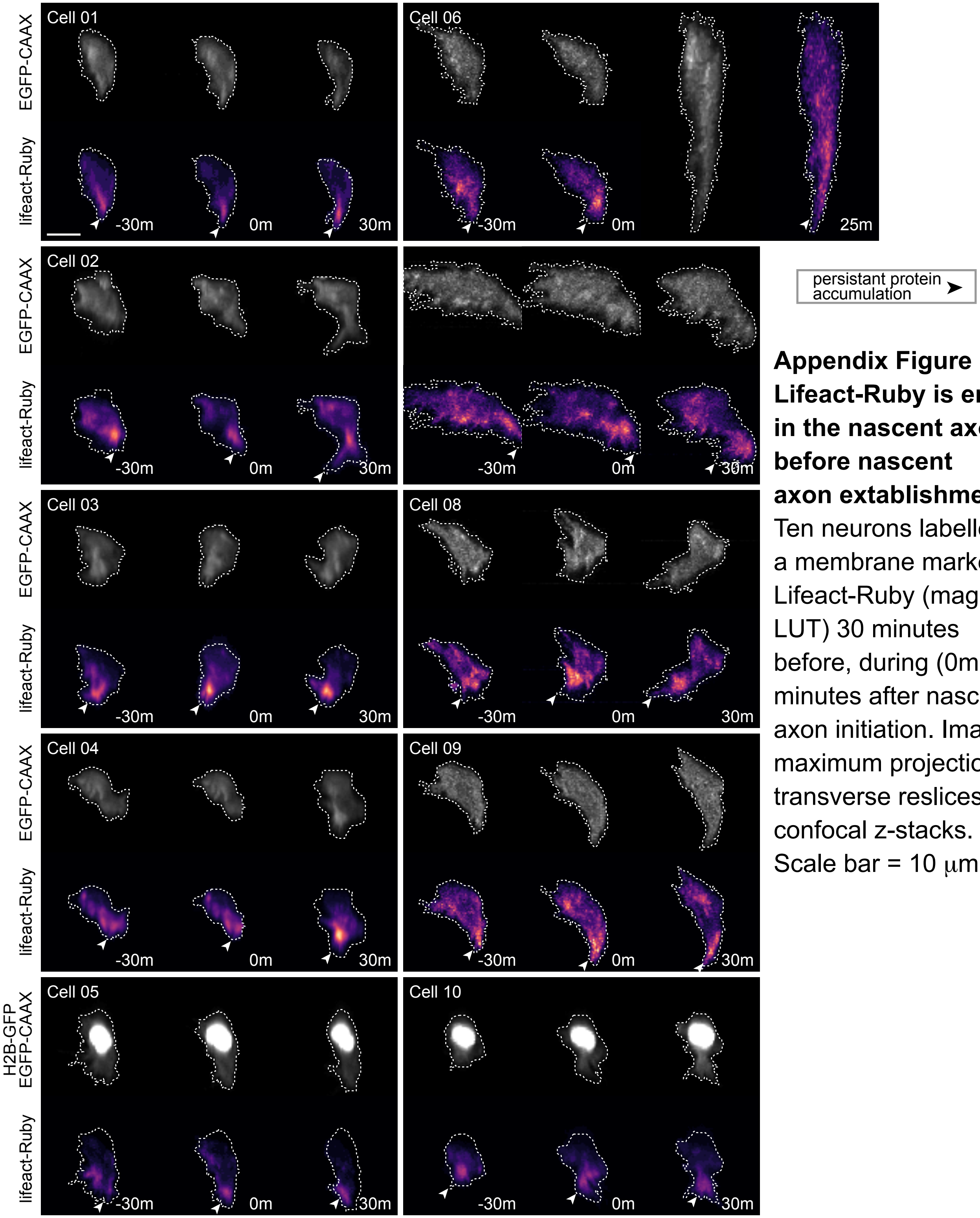

Appendix Figure S3 - Lifeact-Ruby is enriched in the nascent axon before nascent axon extablishment. Ten neurons labelled with a membrane marker and Lifeact-Ruby (magma LUT) 30 minutes before, during (0m) and 30 minutes after nascent axon initiation. Images are maximum projections of transverse reslices of confocal z-stacks. Scale bar = 10 μm.

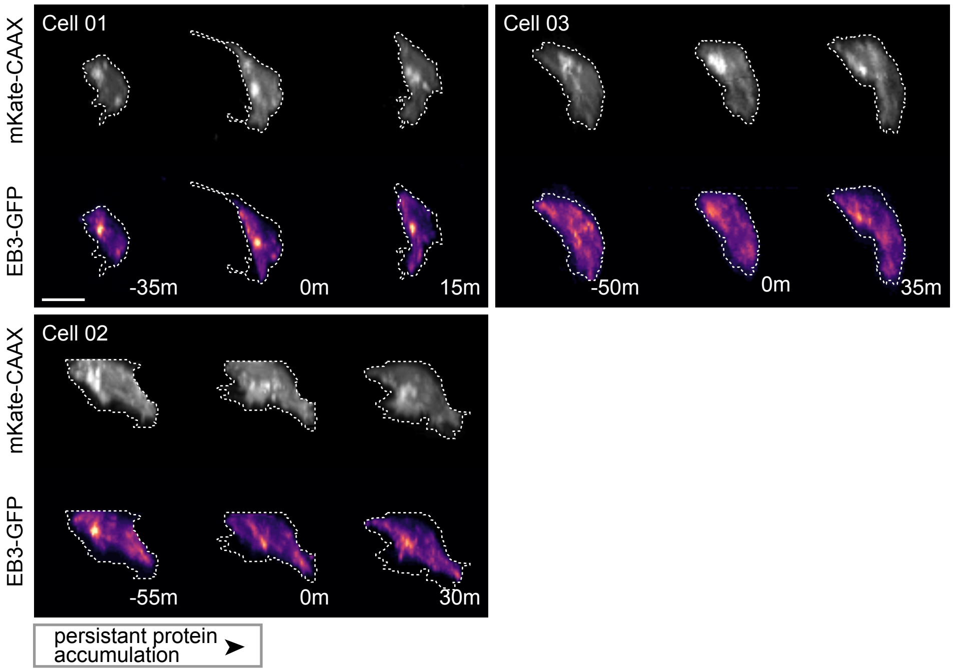

Appendix Figure S4 - EB3-GFP is not enriched in the nascent axon during nascent axon establishment.

Three neurons labelled with a membrane marker and EB3-GFP (magma LUT) 30 minutes before, during (0m) and 30 minutes after nascent axon initiation. Images are maximum projections of transverse reslices of confocal z-stacks. Scale bar = 10 μm.

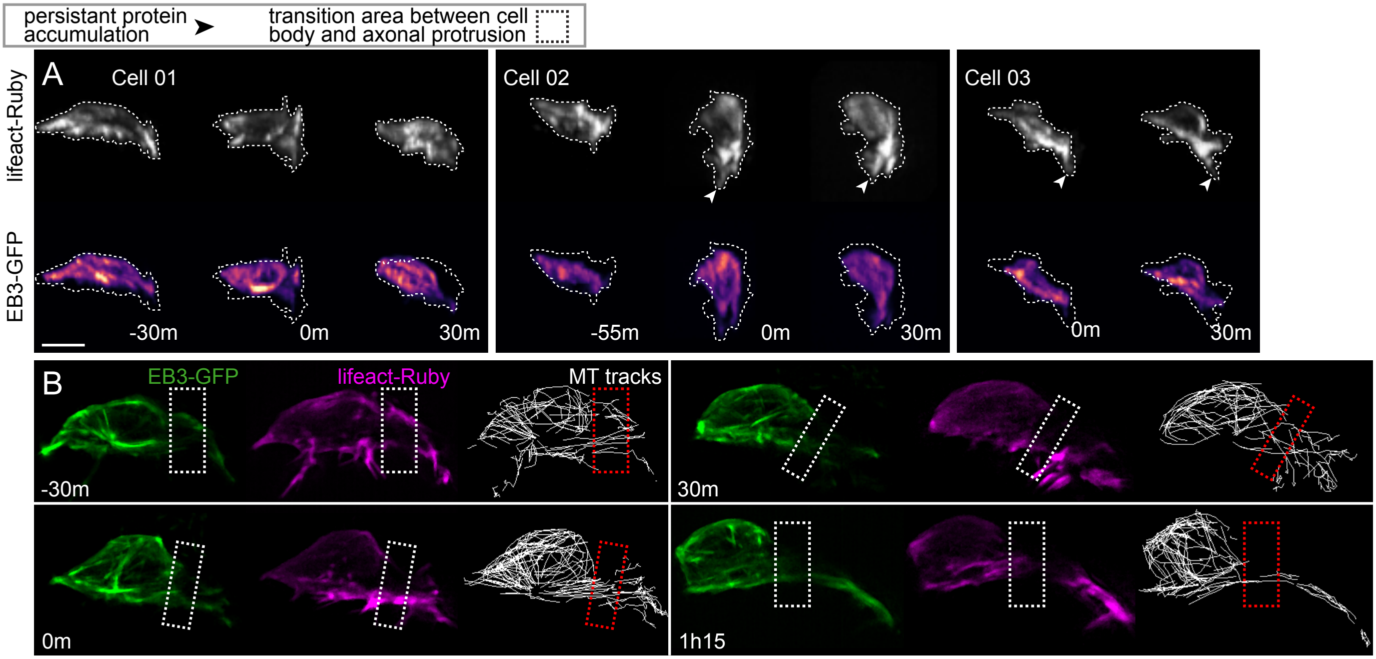

Appendix Figure S5 - Lifeact-Ruby is enriched in the nascent axon before EB3-GFP.

A Three neurons labelled with lifeact-Ruby and EB3-GFP (magma LUT) 30 minutes before, during (0m) and 30 minutes after nascent axon initiation. Images are maximum projections of transverse reslices of confocal z-stacks. Scale bar = 10 μm.

B Maximum projections of neuron in Figure 4C showing EB3-GFP, lifeact-Ruby and microtubule tracks before, during (0m) and after axon initiation.

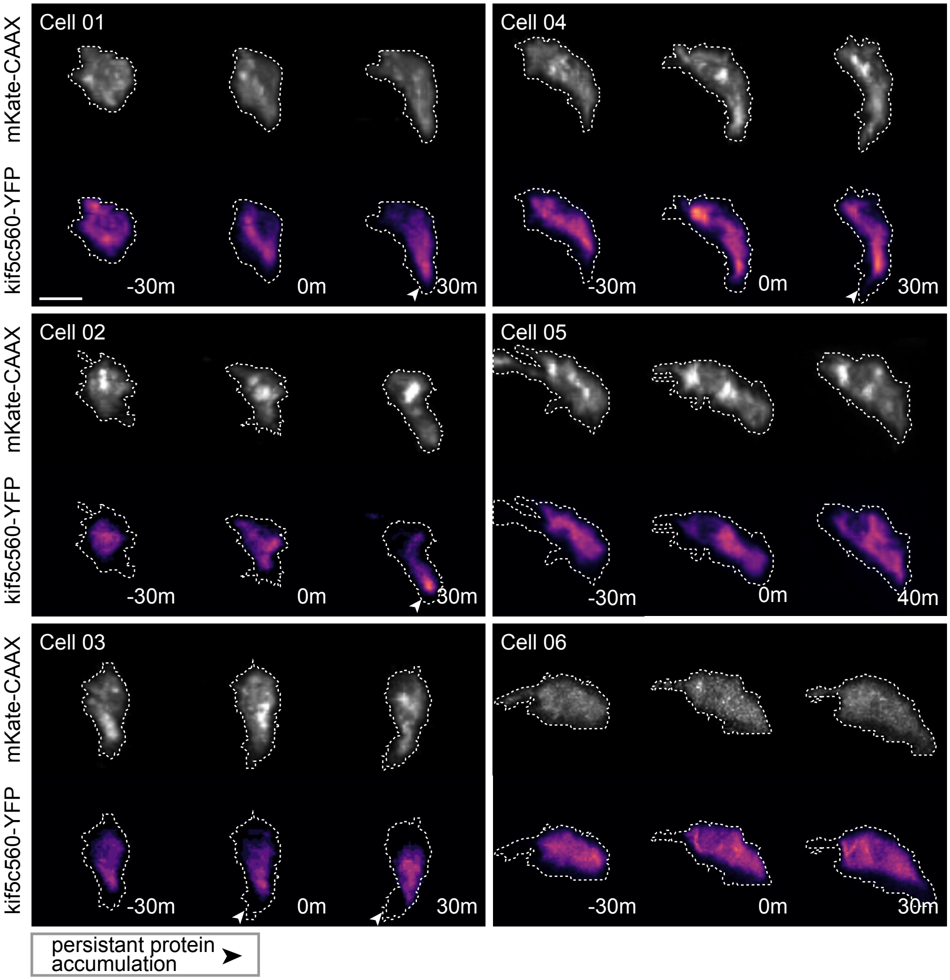

Appendix Figure S6 - Kif5c560-YFP is enriched in the nascent axon following nascent axon extablishment.

Six neurons labelled with a membrane marker and kif5c560-YFP (magma LUT) 30 minutes before, during (0m) and 30 minutes after nascent axon initiation. Images are maximum projections of transverse reslices of confocal z-stacks. Scale bar = 10 μm.

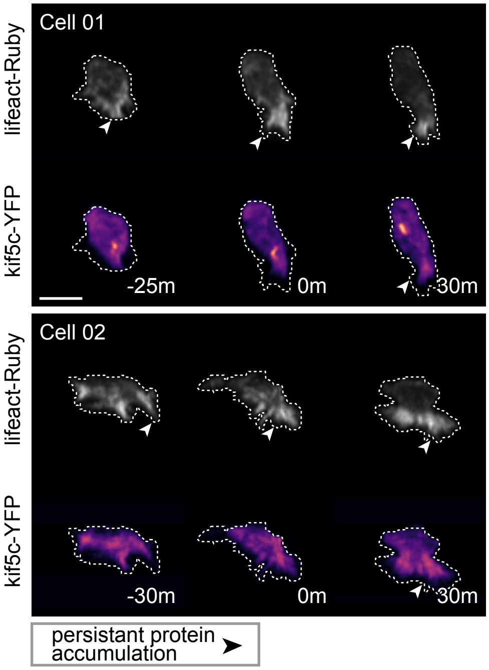

Appendix Figure S7 - Lifeact-Ruby is enriched in the nascent axon before kif5c560-YFP. Two neurons labelled with lifeact-Ruby and kif5c560-YFP (magma LUT) 30 minutes before, during (0m) and 30 minutes after nascent axon initiation. Images are maximum projections of transverse reslices of confocal z-stacks. Scale bar = 10 μm.

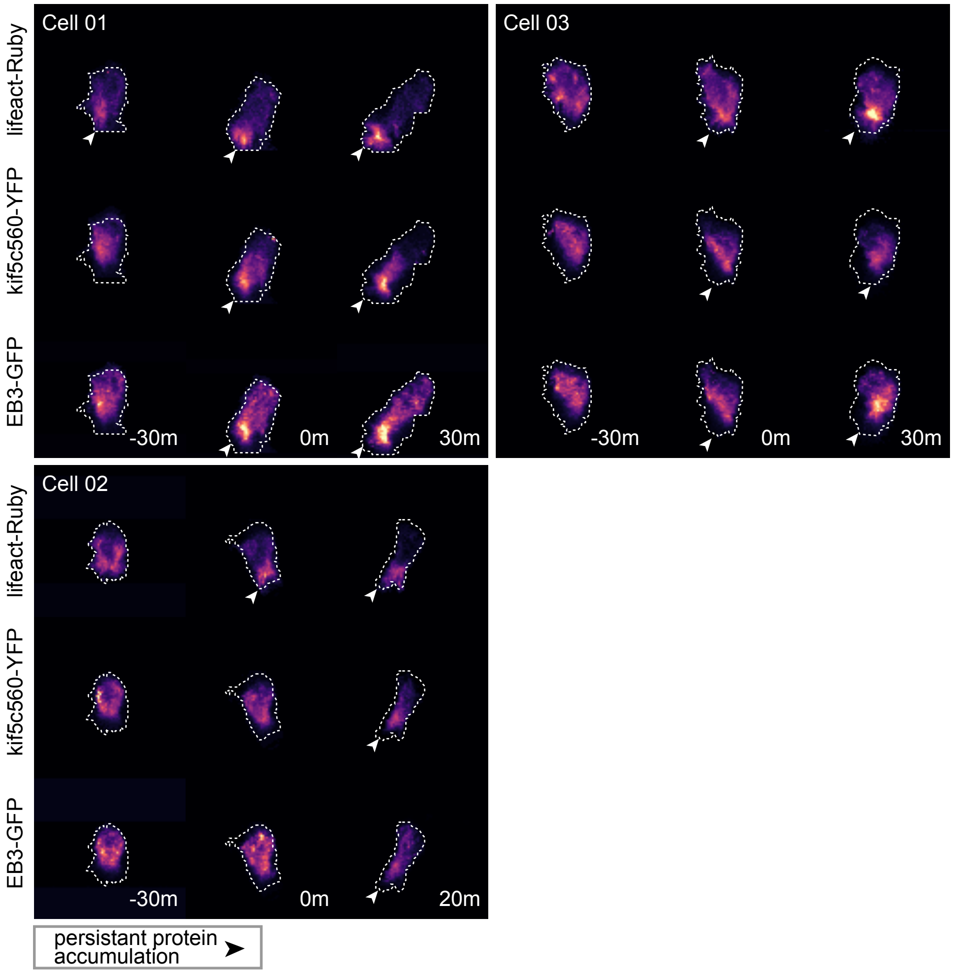

Appendix Figure S8 - Lifeact-Ruby is enriched in the nascent axon before EB3-GFP or kif5c560-YFP.

Four neurons labelled with lifeact-Ruby, kif5c560-YFP and EB3-GFP (all magma LUT) 30 minutes before, during (0m) and 30 minutes after nascent axon initiation. Images are maximum projections of transverse reslices of confocal z-stacks. Scale bar = 10 µm.

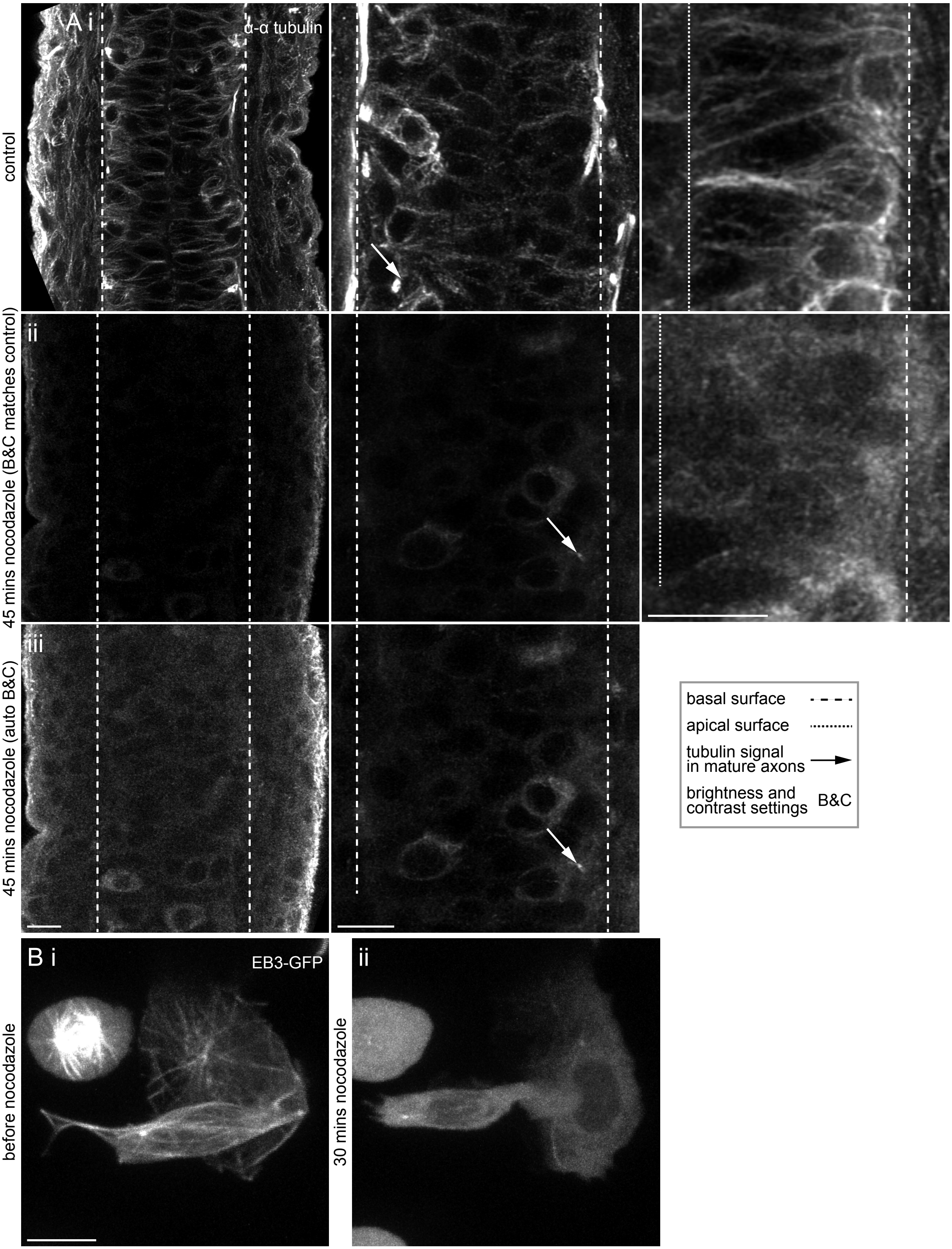

A i
α-α tubulin
control
ii
45 mins nocodazole (B&C matches control)
iii
45 mins nocodazole (auto B&C)
basal surface
apical surface
tubulin signal in mature axons
brightness and contrast settings
B&C
B i
EB3-GFP
before nocodazole
ii
30 mins nocodazole

Appendix Figure S9 - Nocodazole treatment completely disrupts the microtubule array in newborn neurons and neuroepithelial cells.

A Maximum projections of shallow z-stacks showing zebrafish embryos incubated in (i) fish water (control), or (ii, iii) in 5 μg/mL nocodazole for 45 minutes and processed for immunohistochemistry against α-tubulin. Three panels showing three different embryos imaged at different magnifications are shown for each condition. (ii) and (iii) show the same image but with brightness and contrast adjested to be the same as control (ii) or automatically adjusted (iii). Control images show clear microtubule arrays in all cells. After 45 minutes of nocodazole treatment the microtubule array is completely disrupted in neuroepithelial cells and newborn neurons, although remnants can be seen in mature neurons and their axons.

B Maximum projections of cells labelled with EB3-GFP before and after nocodazole treatment. EB3 comets labelling microtubule plus-ends present before nocodazole treatment are not visible within 30 minutes of nocodazole treatment.

Scale bars = 10 μm.

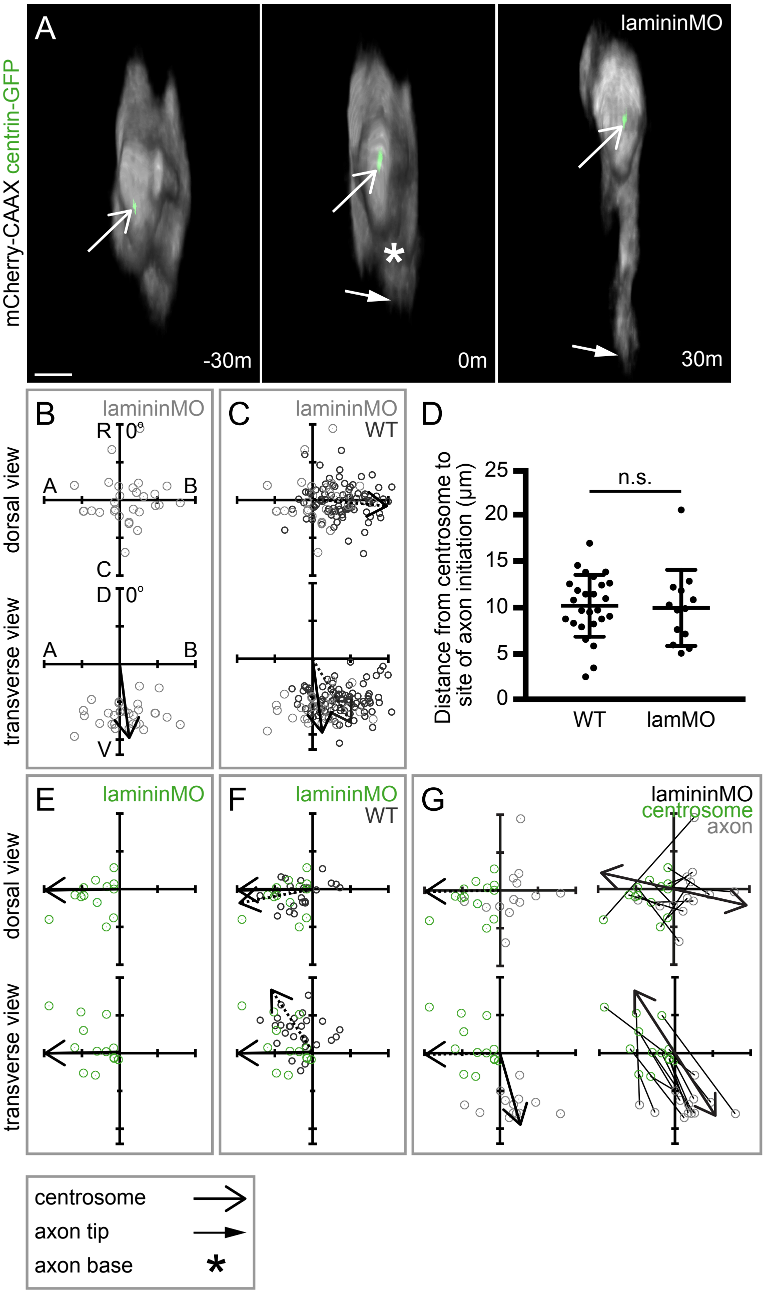

A
mCherry-CAAX centrin-GFP
lamininMO
-30m
0m
30m
B
lamininMO
dorsal view
transverse view
R
0°
A
B
C
D
0°
V
C
lamininMO
WT
D
Distance from centrosome to site of axon initiation (µm)
n.s.
25
20
15
10
5
0
WT
lamMO
E
lamininMO
F
lamininMO
WT
G
lamininMO
centrosome
axon
centrosome
axon tip
axon base

Appendix Figure S10 - LamMO-injected embryos have similar phenotype to *Sly*$^{-/-}$ embryos.

A Image sequence from confocal time lapse shows a neuron in a lamMO-injected embryo labelled with membrane and centrosome markers before (-30m), during (0m) and after (30m) axon initiation. Images are transverse reconstructions from confocal z-stacks.

B Plots showing axon position on the soma relative to the cell centroid at 0,0 for dorsal and transverse view in lamMO-injected embryos (n = 29 cells from three experiments). Axon position is random in dorsal view ($0.1 < P < 0.5$) but not in transverse view ($P < 0.001$, mean = 172.6^{o}; Moore's modification of Rayleigh's test).

C Plots showing merge of WT and lamMO axon positions on the cell body relative to the cell centroid at 0,0 for dorsal and transverse views. WT: n = 86 cells from 8 experiments; lamMO: n = 29 cells from three experiments. Axon positions in WT and lamMO-injected embryos are significantly different (dorsal view $0.01 < P < 0.02$, transverse view $P < 0.001$; Batschelet's alternative to Hotelling test).

D Graph showing distance between centrosome and base of axon at time of axon initiation in WT and lamMO-injected embryos. Bars show mean and standard deviation. WT: n = 26 cells from three experiments, mean = 10.13 μm, s.d. 3.35; lamMO: n = 13 cells from three experiments, mean = 9.9 μm, s.d. = 4.11. One-way ANOVA, $P = 0.980$.

E Plots showing centrosome position relative to the cell centroid at 0,0 for dorsal and transverse view in lamMO-injected embryos (n = 13 cells from three experiments). Centrosome position is not random (dorsal view $P < 0.001$, mean = -91.0^{o}; transverse view $P < 0.001$, mean = -91.1^{o}; Moore's modification of Rayleigh's test).

F Plots showing merge of WT and lamMO centrosome positions on the cell body relative to the cell centroid at 0,0 for dorsal and transverse views. WT: n = 26 cells from three experiments. lamMO: n = 13 cells from three experiments. Centrosome positions are not significantly different between WT and lamMO-injected embryos in dorsal view ($0.2 < P < 0.5$) but are in transverse view ($0.02 < P < 0.05$; Batschelet's alternative to Hotelling test).

G Plots showing positions of the centrosome and base of axon in lamMO-injected embryos at the time of axon initiation relative to the cell centroid at 0,0 for dorsal and transverse view (n = 13 cells from three experiments). Left-hand plots: centrosome position is not random (dorsal view $P < 0.001$; mean = -91.0^{o}; transverse view $P < 0.001$, mean = -91.1^{o}) and axon position is random in dorsal view ($0.05 < P < 0.1$) but not in transverse view ($P < 0.001$, mean = 164.1^{o}; Moore's modification of Rayleigh's test). Centrosome and axon positions are significantly different (dorsal view $P < 0.001$, transverse view $P < 0.001$; Moore's test for paired data). Right-hand plots: vectors linking centrosome and nascent axon from the same cell are not random (dorsal view $P < 0.001$, mean = 102.6^{o}; transverse view $P < 0.001$, mean = 147.1^{o}).

Scale bars = 10 μm.
