# Supplementary material for: Microtubules are not required to generate a nascent axon in embryonic spinal neurons in vivo
Source: EMBO Rep. 2022 Oct 4;23(11):e52493. doi: 10.15252/embr.202152493 (PMC9638849; doi:10.15252/embr.202152493)
Supplement: Supplementary file 22 [file EMBR-23-e52493-s020.pdf]

EMBO reports

Microtubules are not required to generate a nascent axon in embryonic spinal neurons *in vivo*

Rachel Moore, Sinziana Pop, Cache Alleyne and Jonathan Clarke
DOI: 10.15252/embr.202152493

Corresponding author(s): Jonathan Clarke (jon.clarke@kcl.ac.uk)

Review Timeline:

Submission Date:	20th Jan 21
Editorial Decision:	8th Feb 21
Revision Received:	20th Feb 22
Editorial Decision:	11th Apr 22
Revision Received:	5th Sep 22
Accepted:	8th Sep 22

Editor: Esther Schnapp

Transaction Report:

(Note: With the exception of the correction of typographical or spelling errors that could be a source of ambiguity, letters and reports are not edited. Depending on transfer agreements, referee reports obtained elsewhere may or may not be included in this compilation. Referee reports are anonymous unless the Referee chooses to sign their reports.)

Dear Prof. Clarke,

Thank you for the submission of your manuscript to EMBO reports. We have now received the full set of referee reports that is pasted below.

As you will see, all referees acknowledge that the findings are potentially interesting. However, they also point out that the data should be improved and strengthened. I think all comments are reasonable and should be addressed, especially the points raised by referee 2. Testing the role of the centrosome might be less important, but I will ask the referees now for cross-comments on this point. If you disagree or have any other comments, we can also discuss the revisions by video chat, just let me know when is a good time for you.

I would thus like to invite you to revise your manuscript with the understanding that the referee concerns must be fully addressed and their suggestions taken on board. Please address all referee concerns in a complete point-by-point response. Acceptance of the manuscript will depend on a positive outcome of a second round of review. It is EMBO reports policy to allow a single round of major revision only and acceptance or rejection of the manuscript will therefore depend on the completeness of your responses included in the next, final version of the manuscript.

Revised manuscripts should be submitted within three months of a request for revision; they will otherwise be treated as new submissions. Please contact us if a 3-months time frame is not sufficient for the revisions so that we can discuss this further. You can either publish the study as a short report or as a full article. For short reports, the revised manuscript should not exceed 27,000 characters (including spaces but excluding materials & methods and references) and 5 main plus 5 expanded view figures. The results and discussion sections must further be combined, which will help to shorten the manuscript text by eliminating some redundancy that is inevitable when discussing the same experiments twice. For a normal article there are no length limitations, but it should have more than 5 main figures and the results and discussion sections must be separate.

Regarding data quantification, please specify the number "n" for how many independent experiments were performed, the bars and error bars (e.g. SEM, SD) and the test used to calculate p-values in the respective figure legends. This information must be provided in the figure legends. Please also include scale bars in all microscopy images.

IMPORTANT NOTE: we perform an initial quality control of all revised manuscripts before re-review. Your manuscript will FAIL this control and the handling will be DELAYED if the following APPLIES:
1) A data availability section providing access to data deposited in public databases is missing. If you have not deposited any data, please add a sentence to the data availability section that explains that.
2) Your manuscript contains statistics and error bars based on n=2. Please use scatter blots in these cases. No statistics should be calculated if n=2.

When submitting your revised manuscript, please carefully review the instructions that follow below. Failure to include requested items will delay the evaluation of your revision.

1) a .docx formatted version of the manuscript text (including legends for main figures, EV figures and tables). Please make sure that the changes are highlighted to be clearly visible.

2) individual production quality figure files as .eps, .tif, .jpg (one file per figure).
See https://wol-prod-cdn.literatumonline.com/pb-assets/embo-site/EMBOPress_Figure_Guidelines_061115-1561436025777.pdf for more info on how to prepare your figures.

3) We replaced Supplementary Information with Expanded View (EV) Figures and Tables that are collapsible/expandable online. A maximum of 5 EV Figures can be typeset. EV Figures should be cited as 'Figure EV1, Figure EV2" etc... in the text and their respective legends should be included in the main text after the legends of regular figures.

- For the figures that you do NOT wish to display as Expanded View figures, they should be bundled together with their legends in a single PDF file called *Appendix*, which should start with a short Table of Content. Appendix figures should be referred to in the main text as: "Appendix Figure S1, Appendix Figure S2" etc. See detailed instructions regarding expanded view here: <https://www.embopress.org/page/journal/14693178/authorguide#expandedview>

- Additional Tables/Datasets should be labeled and referred to as Table EV1, Dataset EV1, etc. Legends have to be provided in a separate tab in case of .xls files. Alternatively, the legend can be supplied as a separate text file (README) and zipped together with the Table/Dataset file.

4) a .docx formatted letter INCLUDING the reviewers' reports and your detailed point-by-point responses to their comments. As part of the EMBO Press transparent editorial process, the point-by-point response is part of the Review Process File (RPF), which will be published alongside your paper.

5) a complete author checklist, which you can download from our author guidelines <https://www.embopress.org/page/journal/14693178/authorguide>. Please insert information in the checklist that is also reflected in the manuscript. The completed author checklist will also be part of the RPF.

6) Please note that all corresponding authors are required to supply an ORCID ID for their name upon submission of a revised manuscript (<https://orcid.org/>). Please find instructions on how to link your ORCID ID to your account in our manuscript tracking system in our Author guidelines
<https://www.embopress.org/page/journal/14693178/authorguide#authorshipguidelines>

7) Before submitting your revision, primary datasets produced in this study need to be deposited in an appropriate public database (see https://www.embopress.org/page/journal/14693178/authorguide#datadeposition). Please remember to provide a reviewer password if the datasets are not yet public. The accession numbers and database should be listed in a formal "Data Availability" section placed after Materials & Method (see also https://www.embopress.org/page/journal/14693178/authorguide#datadeposition). Please note that the Data Availability Section is restricted to new primary data that are part of this study. * Note - All links should resolve to a page where the data can be accessed. *
If your study has not produced novel datasets, please mention this fact in the Data Availability Section.

8) We would also encourage you to include the source data for figure panels that show essential data. Numerical data should be provided as individual .xls or .csv files (including a tab describing the data). For blots or microscopy, uncropped images should be submitted (using a zip archive if multiple images need to be supplied for one panel). Additional information on source data and instruction on how to label the files are available at
<https://www.embopress.org/page/journal/14693178/authorguide#sourcedata>.

9) Our journal also encourages inclusion of *data citations in the reference list* to directly cite datasets that were re-used and obtained from public databases. Data citations in the article text are distinct from normal bibliographical citations and should directly link to the database records from which the data can be accessed. In the main text, data citations are formatted as follows: "Data ref: Smith et al, 2001" or "Data ref: NCBI Sequence Read Archive PRJNA342805, 2017". In the Reference list, data citations must be labeled with "[DATASET]". A data reference must provide the database name, accession number/identifiers and a resolvable link to the landing page from which the data can be accessed at the end of the reference. Further instructions are available at https://www.embopress.org/page/journal/14693178/authorguide#referencesformat

We would also welcome the submission of cover suggestions, or motifs to be used by our Graphics Illustrator in designing a cover.

As part of the EMBO publication's Transparent Editorial Process, EMBO reports publishes online a Review Process File (RPF) to accompany accepted manuscripts. This File will be published in conjunction with your paper and will include the referee reports, your point-by-point response and all pertinent correspondence relating to the manuscript.

You are able to opt out of this by letting the editorial office know (emboreports@embo.org). If you do opt out, the Review Process File link will point to the following statement: "No Review Process File is available with this article, as the authors have chosen not to make the review process public in this case."

I look forward to seeing a revised version of your manuscript when it is ready. Please let me know if you have questions or comments regarding the revision.

Kind regards,
Esther

Esther Schnapp, PhD
Senior Editor
EMBO reports

Referee #1:

During neuronal polarization, one neurite elongates more than the other and becomes the axon. One important question in neurobiology is the mechanism by which only one neurite elongates during this transitional period. In this study, Moore et al. explore this morphological differentiation in embryonic zebrafish spinal neurons.

The authors showed that the first spinal neurons underwent somal translocation, apical detachment, and the transient formation

of a "multipolar stage" before the initial axon formation. They described that during axon formation microtubules and kif5c populated the initial axonal protrusion together with the enrichment of F-actin at the tip of this protrusion. The centrosome and Golgi are not localized close to the place of this initial elongation. However, these organelles eventually migrate at the base of the growing axon. Moreover, they show that microtubules disruption does not affect initial protrusion and the accumulation of F-actin, although the axon elongation is ultimately precluded. Finally, lack of laminin does not affect drastically the position of axon emergence.

First, it is highly commendable that an effort was made to tackle such a fundamentally important problem in neuronal polarity. Specifically, analyzing neuronal differentiation in vivo. The authors use an elegant approach to describe axon formation in vivo and the data is nicely presented. Having said this, a number of issues should be considered to have a good interpretation of their results.

Some points to be considered are listed below:

• I suggest the authors consider that in situ (developing cortex) it was already shown that the motility of the centrosome precedes the formation of an axon (e.g., Calderon de Anda et al. J. Neurosci. 2010 and Sakakibara et al. Cerebral Cortex, 2014). Specifically, it was shown that in multipolar cells the centrosome translocates towards the neurite that eventually grows as an axon. Although it is not the same model system used in the current manuscript, it seems that the centrosome/Golgi consistently translocates at the base of the growing axon. Whether this is before or after the initial extension is not the crucial point to prove (according to me), but the involvement of these organelles in the axonal fate. In other words, why these organelles consistently move at the base of the growing axon?

• Therefore, I suggest the authors directly affect centrosomal activities to test the potential role of the centrosome in axonal fate.

• Nocodazole is affecting all microtubules (centrosomal and acentrosomal ones). Thus, is not a suitable approach to test the role of centrosomal activities in axonal fate. In this regard, Stiess et al, Science, 2010 showed that axon elongation (not initiation) is independent of centrosomal microtubules. Contrary to this, the authors stated (page 22): "Combining these findings and others (e.g. (Stiess et al., 2010)) suggests that centrosome proximity is not required for axon initiation but is required for axon growth in vertebrates, at least at early stages". This has to be corrected to avoid misleading the readers.

Referee #2:

Report:
In their manuscript "Microtubules are not required to generate a nascent axon in new-born spinal neurons in vivo" Moore et al used the embryonic Zebrafish model to analyze axon initiation by spinal neurons in vivo. With focus in cytoskeletal requirements, the authors stablished a comparison between the morphogenetic steps leading to axon initiation in vivo by spinal neurons and those from currently available in vitro neuronal models. In particular, the authors addressed the role and hierarchy of the actin and microtubule networks during axon initiation and scrutinize the contribution of structures and organelles deemed potential regulators of axon initiation (i.e. the Centrosome/MTOC and the Golgi network). The authors found that the accumulation of the actin cytoskeleton precedes microtubule in the initiation of the prospective axon in spinal neurons. The authors presented evidences indicating that the microtubule system does not contribute to the initiation of the axon but is instead required for axon extension and maintenance. Furthermore, the authors showed that the Centrosome/MTOC and the Golgi apparatus typically located opposite to the axon initiation site and only relocated to the base of the axon during pathfinding and elongation, thus indicating they are not required to support axon initiation.

Overall, the manuscript is very interesting, well written and nicely structured. The in-vivo approach is impressive. Most of the key findings of this study are well backed by the presented data. However, the evidences supporting its major claim "microtubules are not required for nascent axon establishment" are not convincing enough.

General comments

The authors clearly show that an axon cannot proceed as such without the support of the microtubule network. Moreover, the actin-based protrusion the authors define as nascent axon requires microtubules, since it will not only fail to elongate but will eventually also retract without them, indication of a rather transient structure. In addition, the relatively small number of neurons that manage to develop a nascent axon in absence of microtubules compared to the cell pool analyzed appears to indicate an exception rather than a rule (Please see below in Major concerns for more detail). In consequence, the authors may have overinterpreted some of the evidence leading to their major finding.

In summary, the authors convincingly show that reorganization/accumulation of the actin cytoskeleton precedes microtubule invasion during axon initiation. However, the same data suggest that actin cytoskeleton functions more like a landmark for initiation (or a MT capturing platform) rather than a scaffold capable of mounting a stable axon-like protrusion on its own.

Therefore, this reviewer considers that the authors should likely downturn their claim on the non-essential status of microtubules during axon initiation in spinal neurons unless more convincing evidences could be presented. Accordingly, this manuscript should be atoned in light of the present arguments before being considered for publication by EMBO reports.

Major points

➢ In the experiments involving Nocodazole treatment (related to Figure 4), the authors indicate they analyzed 54 cells, from which 83% (45/54) retracted their protrusions - and some even their proto-axonal process- after Nocodazole treatment. Later, (45m) into Nocodazole treatment only 25% of these neurons (14/54) developed a de novo protrusion, enriched with F-actin and with characteristics reminiscent of a nascent axon. This is basically the central observation holding the main claim of this work that microtubules are not necessary to initiate an axon in vivo. However, the data also show that 75% of the neurons (40/54) fail to developed a de novo axon-like protrusion (i.e. fail to initiate an axon). Are these 75% of cells not accounted for? What is the significance of the 14 neurons that develop a nascent axon against the other 40 neurons that did not? Causality is not clear at this point.

➢ Although Nocodazole treatment largely (typically a better word than completely in these cases) disassemble the microtubule network it is yet possible that few remnants of the network could still be present in the analyzed neurons and be sufficient to contribute to the initiation of the nascent axon. In this line, the authors should determine the status of the microtubule network in the individual neurons they analyzed, for instance by fixing and staining the cells against α-Tubulin at the end of their time lapses. Such experiment would facilitate the assessment of their claim.

➢ In order to better compare how Nocodazole treatment affect spinal neuron dynamics, it would be useful to have a similar analysis as the one shown in Figures 4A-G but for non-treated neurons. Also reporting the efficiency of axon initiation in non-treated neurons would be good for comparison. For instance, if analyzing a similar amount of non-treated neurons (approx. 50 cells), how many of them will initiate an axon the same time frame as for the Nocodazole treated ones?

➢ It is not clear how the grouping for quantification was performed in 4C-G. Were both non-axonal and axonal protrusions parameters measured in two groups of 16 neurons and then combined and averaged? Or were both type of protrusions measured in a single group of 16 cells? If so, which was the rationale to select the cells analyzed from the 54-cells group? Moreover, the text indicates that 14/54 neurons had initiated axon like protrusion but in the graphs the number of axon-like protrusion reported is 16. Does this mean that some cells extended more than 2 protrusions?

➢ In Figure 4 H, the kif5c-YFP signal at 144m does not correctly overlaps with its corresponding mKate-CAAX signal (which labels the cellular membrane limits) as do the rest of panels. Indeed, this image seems displaced left and upwards which would increase the apparent distance from the cell to the arrow. Please Check.

➢ In Figure 3C, it would be interesting to have these cells also stained for α-Tubulin at the different time points to determine whether the little amount of EB3 positive microtubules observed also reflect a generally reduced microtubule network in the proximity of the nascent axon or just reduced EB3-labelled microtubules in that area. Kif5c-YPF sorting Figure 3B seems to support the presence of a microtubule network reaching more distal locations than those estimated by EB3 analysis.

Minor comments:

Page 7-line 7: "After pausing the axon extended rapidly and developing a growth cone" should read "After pausing, the axon extended rapidly developing a growth cone".

Page 13-line 24 In regard to the sentence "This was surprising, as during neurite formation in vitro, microtubules protrude further into the periphery in areas that will subsequently form a neurite than those that don't (Flynn et al., 2012)". The work by Flynn et al showed that microtubules protrude towards the cell periphery when the dynamism of the actin cytoskeleton allows it. Indeed, this work showed that dynamic actin structures guide and bias microtubules to engorge filopodia that later typically become a nascent neurite, pretty much as the behavior of the actin filaments observed in this study. And the data showed that stabilizing microtubules with taxol does not enhance neurite formation.

Page 18-line 26 "The mechanisms that may define axon initiation have been studied for several decades but many of the previous works are compromised by studying this process in neurons growing in vitro that lack the complex 3-dimensional architecture and molecular environment of in vivo settings". Over this particular point, and to enrich the discussion, it should be noted that advances have been made towards understanding de the cytoskeleton dynamics of axon formation through the study of neuron polarization in 3D matrices (e.g. Santos et al., 2020; https://doi.org/10.1016/j.celrep.2020.107907).

Referee #3:

The manuscript by Moore et al provides the first in vivo analysis of the role of cytoskeletons in axon formation using zebrafish spinal sensory neurons as a model. Following an early study from the author's group that examined differentiation of these neurons (Hadjivasiliou et al, 2019), the current study expanded live cell imaging analysis during the formation of nascent axons. Using a variety of localization and functional markers, the authors demonstrate the localization of each cytoskeletal element (centrosome, actin, and microtubule) in relationship with the nascent axons. Importantly, using a depolymerizing drug, the authors show that microtubules are not required for the nascent axon formation, an observation that contradicts some earlier findings based on cultured neurons in vitro. Finally, using both mutants and morpholinos, the authors demonstrate the role of laminin in serving as a position cue for axon initiation. Overall, based on well-designed spatial and temporal analyses, the manuscript describes several exciting and novel findings of cellular regulation during initial axon development in vivo and provides new insights into the underlying mechanisms that could be interesting to both cell biologists and neurobiologists. Several points described below might be considered by the authors to broaden the significance of their interesting findings.

1) The title emphasizes on the requirement of microtubules in nascent axon formation, but the data presented have more interesting observations than that related to microtubule alone. Consider the change of the title to provide a balanced view of the data.
2) The localization of F-actin to nascent axons suggests a critical role, but does not provide a full test of the requirement of this localization. Have the authors done a similar pharmacology experiment similar to microtubule inhibition?
3) In the discussion (p.20-21), the authors suggest nascent axons without microtubules are "less stable .. and eventually tend to retract". Is this statement based on any experimental evidence? If so, it is very important because it indicates a role of microtubules in axon extension after initiation. Including such data would provide a more complete picture of the role of microtubules and strengthen the significance of the paper.
4) Description of how microtubule tracks were generated in Fig. 3D is missing.

Please find our revised manuscript, which we hope is now acceptable as a Research Article for EMBO Reports. I apologise that this resubmission has taken so long, but this was due to the lead researcher's maternity leave and my own retirement during this period. We believe we have now addressed all the referees' comments (with the exception of testing more precisely the role of the centrosome – on which we agree with your comment and the referees' cross-comments that this point should not be required for this work). We have tightened up the clarity of the writing and data presentation throughout the manuscript in addition to addressing the referees' comments. We have made small amendments to Title and Abstract.

Here are the Referees' reports in full, followed by a point by point discussion of how we have modified our manuscript in response to the very helpful referees' comments (with our responses in red text below, and highlighted in yellow in the manuscript).

Referee #1:

During neuronal polarization, one neurite elongates more than the other and becomes the axon. One important question in neurobiology is the mechanism by which only one neurite elongates during this transitional period. In this study, Moore et al. explore this morphological differentiation in embryonic zebrafish spinal neurons.

The authors showed that the first spinal neurons underwent somal translocation, apical detachment, and the transient formation of a "multipolar stage" before the initial axon formation. They described that during axon formation microtubules and kif5c populated the initial axonal protrusion together with the enrichment of F-actin at the tip of this protrusion. The centrosome and Golgi are not localized close to the place of this initial elongation. However, these organelles eventually migrate at the base of the growing axon. Moreover, they show that microtubules disruption does not affect initial protrusion and the accumulation of F-actin, although the axon elongation is ultimately precluded. Finally, lack of laminin does not affect drastically the position of axon emergence.

First, it is highly commendable that an effort was made to tackle such a fundamentally important problem in neuronal polarity. Specifically, analyzing neuronal differentiation in vivo. The authors use an elegant approach to describe axon formation in vivo and the data is nicely presented. Having said this, a number of issues should be considered to have a good interpretation of their results.

Some points to be considered are listed below:

• I suggest the authors consider that in situ (developing cortex) it was already shown that the motility of the centrosome precedes the formation of an axon (e.g., Calderon de Anda et al. J. Neurosci. 2010 and Sakakibara et al. Cerebral Cortex, 2014). Specifically, it was shown that in multipolar cells the centrosome translocates towards the neurite that eventually grows as an axon. Although it is not the same model system used in the current manuscript, it seems that the centrosome/Golgi consistently translocates at the base of the growing axon. Whether this is before or after the initial extension is not the crucial point to prove (according to me),

but the involvement of these organelles in the axonal fate. In other words, why these organelles consistently move at the base of the growing axon?

• Therefore, I suggest the authors directly affect centrosomal activities to test the potential role of the centrosome in axonal fate.

• Nocodazole is affecting all microtubules (centrosomal and acentrosomal ones). Thus, is not a suitable approach to test the role of centrosomal activities in axonal fate. In this regard, Stiess et al, Science, 2010 showed that axon elongation (not initiation) is independent of centrosomal microtubules. Contrary to this, the authors stated (page 22): "Combining these findings and others (e.g. (Stiess et al., 2010)) suggests that centrosome proximity is not required for axon initiation but is required for axon growth in vertebrates, at least at early stages". This has to be corrected to avoid misleading the readers.

Referee #2:

Report:
In their manuscript "Microtubules are not required to generate a nascent axon in new-born spinal neurons in vivo" Moore et al used the embryonic Zebrafish model to analyze axon initiation by spinal neurons in vivo. With focus in cytoskeletal requirements, the authors stablished a comparison between the morphogenetic steps leading to axon initiation in vivo by spinal neurons and those from currently available in vitro neuronal models. In particular, the authors addressed the role and hierarchy of the actin and microtubule networks during axon initiation and scrutinize the contribution of structures and organelles deemed potential regulators of axon initiation (i.e. the Centrosome/MTOC and the Golgi network). The authors found that the accumulation of the actin cytoskeleton precedes microtubule in the initiation of the prospective axon in spinal neurons. The authors presented evidences indicating that the microtubule system does not contribute to the initiation of the axon but is instead required for axon extension and maintenance. Furthermore, the authors showed that the Centrosome/MTOC and the Golgi apparatus typically located opposite to the axon initiation site and only relocated to the base of the axon during pathfinding and elongation, thus indicating they are not required to support axon initiation.

Overall, the manuscript is very interesting, well written and nicely structured. The in-vivo approach is impressive. Most of the key findings of this study are well backed by the presented data. However, the evidences supporting its major claim "microtubules are not required for nascent axon establishment" are not convincing enough.

General comments

The authors clearly show that an axon cannot proceed as such without the support of the microtubule network. Moreover, the actin-based protrusion the authors define as nascent axon requires microtubules, since it will not only fail to elongate but will eventually also retract without them, indication of a rather transient structure. In addition, the relatively small number of neurons that manage to develop a nascent axon in absence of microtubules compared to the cell pool analyzed appears to indicate an exception rather than a rule (Please see below in Major concerns for more detail). In consequence, the authors may have overinterpreted some of the evidence leading to their major finding.

In summary, the authors convincingly show that reorganization/accumulation of the actin cytoskeleton precedes microtubule invasion during axon initiation. However, the same data suggest that actin cytoskeleton functions more like a landmark for initiation (or a MT capturing platform) rather than a scaffold capable of mounting a stable axon-like protrusion on its own. Therefore, this reviewer considers that the authors should likely downturn their claim on the non-essential status of microtubules during axon initiation in spinal neurons unless more convincing evidences could be presented. Accordingly, this manuscript should be atoned in light of the present arguments before being considered for publication by EMBO reports.

Major points

➢ In the experiments involving Nocodazole treatment (related to Figure 4), the authors indicate they analyzed 54 cells, from which 83% (45/54) retracted their protrusions - and some even their proto-axonal process- after Nocodazole treatment. Later, (45m) into Nocodazole treatment only 25% of these neurons (14/54) developed a de novo protrusion, enriched with F-actin and with characteristics reminiscent of a nascent axon. This is basically the central observation holding the main claim of this work that microtubules are not necessary to initiate an axon in vivo. However, the data also show that 75% of the neurons (40/54) fail to developed a de novo axon-like protrusion (i.e. fail to initiate an axon). Are these 75% of cells not accounted for? What is the significance of the 14 neurons that develop a nascent axon against the other 40 neurons that did not? Causality is not clear at this point.

➢ Although Nocodazole treatment largely (typically a better word than completely in these cases) disassemble the microtubule network it is yet possible that few remnants of the network could still be present in the analyzed neurons and be sufficient to contribute to the initiation of the nascent axon. In this line, the authors should determine the status of the microtubule network in the individual neurons they analyzed, for instance by fixing and staining the cells against α-Tubulin at the end of their time lapses. Such experiment would facilitate the assessment of their claim.

➢ In order to better compare how Nocodazole treatment affect spinal neuron dynamics, it would be useful to have a similar analysis as the one shown in Figures 4A-G but for non-treated neurons. Also reporting the efficiency of axon initiation in non-treated neurons would be good for comparison. For instance, if analyzing a similar amount of non-treated neurons (approx. 50 cells), how many of them will initiate an axon the same time frame as for the Nocodazole treated ones?

➢ It is not clear how the grouping for quantification was performed in 4C-G. Were both non-axonal and axonal protrusions parameters measured in two groups of 16 neurons and then combined and averaged? Or were both type of protrusions measured in a single group of 16 cells? If so, which was the rationale to select the cells analyzed from the 54-cells group? Moreover, the text indicates that 14/54 neurons had initiated axon like protrusion but in the graphs the number of axon-like protrusion reported is 16. Does this mean that some cells extended more than 2 protrusions?

➢ In Figure 4 H, the kif5c-YFP signal at 144m does not correctly overlaps with its corresponding mKate-CAAX signal (which labels the cellular membrane limits) as do the rest of panels. Indeed, this image seems displaced left and upwards which would increase the

apparent distance from the cell to the arrow. Please Check.

➢ In Figure 3C, it would be interesting to have these cells also stained for α-Tubulin at the different time points to determine whether the little amount of EB3 positive microtubules observed also reflect a generally reduced microtubule network in the proximity of the nascent axon or just reduced EB3-labelled microtubules in that area. Kif5c-YPF sorting Figure 3B seems to support the presence of a microtubule network reaching more distal locations than those estimated by EB3 analysis.

Minor comments:

Page 7-line 7: "After pausing the axon extended rapidly and developing a growth cone" should read "After pausing, the axon extended rapidly developing a growth cone".

Page 13-line 24 In regard to the sentence "This was surprising, as during neurite formation in vitro, microtubules protrude further into the periphery in areas that will subsequently form a neurite than those that don't (Flynn et al., 2012)". The work by Flynn et al showed that microtubules protrude towards the cell periphery when the dynamism of the actin cytoskeleton allows it. Indeed, this work showed that dynamic actin structures guide and bias microtubules to engorge filopodia that later typically become a nascent neurite, pretty much as the behavior of the actin filaments observed in this study. And the data showed that stabilizing microtubules with taxol does not enhance neurite formation.

Page 18-line 26 "The mechanisms that may define axon initiation have been studied for several decades but many of the previous works are compromised by studying this process in neurons growing in vitro that lack the complex 3-dimensional architecture and molecular environment of in vivo settings". Over this particular point, and to enrich the discussion, it should be noted that advances have been made towards understanding de the cytoskeleton dynamics of axon formation through the study of neuron polarization in 3D matrices (e.g. Santos et al., 2020; https://doi.org/10.1016/j.celrep.2020.107907).

Referee #3:

The manuscript by Moore et al provides the first in vivo analysis of the role of cytoskeletons in axon formation using zebrafish spinal sensory neurons as a model. Following an early study from the author's group that examined differentiation of these neurons (Hadjivasiliou et al, 2019), the current study expanded live cell imaging analysis during the formation of nascent axons. Using a variety of localization and functional markers, the authors demonstrate the localization of each cytoskeletal element (centrosome, actin, and microtubule) in relationship with the nascent axons. Importantly, using a depolymerizing drug, the authors show that microtubules are not required for the nascent axon formation, an observation that contradicts some earlier findings based on cultured neurons in vitro. Finally, using both mutants and morpholinos, the authors demonstrate the role of laminin in serving as a position cue for axon initiation. Overall, based on well-designed spatial and temporal analyses, the manuscript describes several exciting and novel findings of cellular regulation during initial axon development in vivo and provides new insights into the underlying mechanisms that could be interesting to both cell biologists and neurobiologists. Several points described below might be considered by the authors to broaden the significance of

their interesting findings.

1) The title emphasizes on the requirement of microtubules in nascent axon formation, but the data presented have more interesting observations than that related to microtubule alone. Consider the change of the title to provide a balanced view of the data.
2) The localization of F-actin to nascent axons suggests a critical role, but does not provide a full test of the requirement of this localization. Have the authors done a similar pharmacology experiment similar to microtubule inhibition?
3) In the discussion (p.20-21), the authors suggest nascent axons without microtubules are "less stable .. and eventually tend to retract". Is this statement based on any experimental evidence? If so, it is very important because it indicates a role of microtubules in axon extension after initiation. Including such data would provide a more complete picture of the role of microtubules and strengthen the significance of the paper.
4) Description of how microtubule tracks were generated in Fig. 3D is missing.

Our responses:

Referee #1:

Some points to be considered are listed below:

• I suggest the authors consider that in situ (developing cortex) it was already shown that the motility of the centrosome precedes the formation of an axon (e.g., Calderon de Anda et al. J. Neurosci. 2010 and Sakakibara et al. Cerebral Cortex, 2014). Specifically, it was shown that in multipolar cells the centrosome translocates towards the neurite that eventually grows as an axon. Although it is not the same model system used in the current manuscript, it seems that the centrosome/Golgi consistently translocates at the base of the growing axon. Whether this is before or after the initial extension is not the crucial point to prove (according to me), but the involvement of these organelles in the axonal fate. In other words, why these organelles consistently move at the base of the growing axon?

We have clarified previous work on this point in the Discussion (page 20-21) and noted that centrosomes also move to leading processes in migrating cells, so may not be an axon specific phenomenon.

• Therefore, I suggest the authors directly affect centrosomal activities to test the potential role of the centrosome in axonal fate.

We agree with the Editor and later referee cross-commenting that this is not essential here, as the role of the centrosome in later development of the axon is not the focus of this manuscript.

• Nocodazole is affecting all microtubules (centrosomal and acentrosomal ones). Thus, is not a suitable approach to test the role of centrosomal activities in axonal fate. In this regard, Stiess et al, Science, 2010 showed that axon elongation (not initiation) is independent of centrosomal microtubules. Contrary to this, the authors stated (page 22): "Combining these findings and others (e.g. (Stiess et al., 2010)) suggests that centrosome proximity is not required for axon initiation but is required for axon growth in vertebrates, at least at early

stages". This has to be corrected to avoid misleading the readers.

We corrected this point as requested by removing our misleading sentence from Discussion (page 20-21)

Referee #2:

Report:

In summary, the authors convincingly show that reorganization/accumulation of the actin cytoskeleton precedes microtubule invasion during axon initiation. However, the same data suggest that actin cytoskeleton functions more like a landmark for initiation (or a MT capturing platform) rather than a scaffold capable of mounting a stable axon-like protrusion on its own. Therefore, this reviewer considers that the authors should likely downturn their claim on the non-essential status of microtubules during axon initiation in spinal neurons unless more convincing evidences could be presented. Accordingly, this manuscript should be atoned in light of the present arguments before being considered for publication by EMBO reports.

We argue in the following responses to this referee's major points that our conclusion remains safe.

Major points

➢ In the experiments involving Nocodazole treatment (related to Figure 4), the authors indicate they analyzed 54 cells, from which 83% (45/54) retracted their protrusions - and some even their proto-axonal process- after Nocodazole treatment. Later, (45m) into Nocodazole treatment only 25% of these neurons (14/54) developed a de novo protrusion, enriched with F-actin and with characteristics reminiscent of a nascent axon. This is basically the central observation holding the main claim of this work that microtubules are not necessary to initiate an axon in vivo. However, the data also show that 75% of the neurons (40/54) fail to developed a de novo axon-like protrusion (i.e. fail to initiate an axon). Are these 75% of cells not accounted for? What is the significance of the 14 neurons that develop a nascent axon against the other 40 neurons that did not? Causality is not clear at this point.

We deal with this good point in our reply to the next but one point.

➢ Although Nocodazole treatment largely (typically a better word than completely in these cases) disassemble the microtubule network it is yet possible that few remnants of the network could still be present in the analyzed neurons and be sufficient to contribute to the initiation of the nascent axon. In this line, the authors should determine the status of the microtubule network in the individual neurons they analyzed, for instance by fixing and staining the cells against α-Tubulin at the end of their time lapses. Such experiment would facilitate the assessment of their claim.

We agree that the experiment suggested by the reviewer would be ideal but also a major undertaking for these technically challenging in vivo imaging studies. However we also believe our current analyses already address this point, although not in the same cells that we time-lapse. Our current analyses (Appendix Figures S9A and S9B) show there are no

detectable EB3 comets or α-tubulin positive filaments in newborn neurons or adjacent neuroepithelial cells. Additionally we already show by time-lapse analysis that we cannot detect any differential expression of the microtubule motor Kif5c560 into nascent axons following nocodazole treatment (Figure 5J and K). We think these observations already strongly support our claim that nascent axons can be generated in a microtubule-free manner. While it is formally possible that the 14 nocodazole neurons that make nascent axons could be less affected by nocodazole than all the other neurons we have analysed, this seems a little unlikely. We suspect the referee's worries on this point are heightened by the fact that many nocodazole neurons do not make nascent axons in the imaging period of our experiments, and we address this worry in our reply to the next point.

➢ In order to better compare how Nocodazole treatment affect spinal neuron dynamics, it would be useful to have a similar analysis as the one shown in Figures 4A-G but for non-treated neurons. Also reporting the efficiency of axon initiation in non-treated neurons would be good for comparison. For instance, if analyzing a similar amount of non-treated neurons (approx. 50 cells), how many of them will initiate an axon the same time frame as for the Nocodazole treated ones?

This is a very good point that we now address and discuss. We have now re-analysed our normal neuron data to assess how many control neurons would not generate a nascent axon within the imaging period of 135 minutes used for the nocodazole treated embryos. We found 19/30 (63%) normal neurons produced a nascent axon within 135 minutes of imaging, compared to 14/54 (26%) neurons in nocodazole. Thus 37% of normal neurons do not produce an axon in the experimental time frame. However the efficiency of nascent axon production is halved in nocodazole. We do not know why the efficiency is reduced but we suspect a strong candidate will be that the local cellular (and possibly molecular) environment of the marginal zone is disrupted by nocodazole, as microtubules are also lost from the scaffold of neuroepithelial cells. In fact we have previously shown that apicobasal polarity of neuroepithelial cells requires microtubules (Buckley et al 2013). Thus our experimental neurons are attempting to generate nascent axons not only in the absence of their own microtubule cytoskeleton, but also in a disrupted marginal zone environment. It seems very likely this will negatively affect a neuron's ability to generate a nascent axon. We have added this important data and discussion to Results page 13 to 14 and Discussion page 18 to 19.

➢ It is not clear how the grouping for quantification was performed in 4C-G. Were both non-axonal and axonal protrusions parameters measured in two groups of 16 neurons and then combined and averaged? Or were both type of protrusions measured in a single group of 16 cells? If so, which was the rationale to select the cells analyzed from the 54-cells group? Moreover, the text indicates that 14/54 neurons had initiated axon like protrusion but in the graphs the number of axon-like protrusion reported is 16. Does this mean that some cells extended more than 2 protrusions?

We have included extra information in the Methods (page 25) to explain how we chose the cells to analyse. We thank the reviewer for picking up an error in the number of neurons that initiated an axon in the presence of nocodazole – it is 14/54 and we have corrected the figure legend (Figure 5C-E, G; page 34 to 35).

➢ In Figure 4 H, the kif5c-YFP signal at 144m does not correctly overlaps with its

corresponding mKate-CAAX signal (which labels the cellular membrane limits) as do the rest of panels. Indeed, this image seems displaced left and upwards which would increase the apparent distance from the cell to the arrow. Please Check.

(Old figure 4 is now Figure 5.) The referee is right. We have corrected this displacement in new Fig 5H.

➢ In Figure 3C, it would be interesting to have these cells also stained for α-Tubulin at the different time points to determine whether the little amount of EB3 positive microtubules observed also reflect a generally reduced microtubule network in the proximity of the nascent axon or just reduced EB3-labelled microtubules in that area. Kif5c-YPF sorting Figure 3B seems to support the presence of a microtubule network reaching more distal locations than those estimated by EB3 analysis.

We agree the precise relationship between the microtubule network and EB3 positive microtubules would be interesting to know, but it would be very challenging to get to this comparison in individual neurons. To clarify our findings we have reorganised and rewritten this whole section (page 11 – 12). We are careful now to say in Results and Discussion that our analysis of nascent axon formation compared the relative positions of F-actin to the enrichment of growing tips of microtubules, so we do not think our conclusions are misleading. We think the merged images in new Figure 4E convincingly show F-actin is more distal than the enrichments of EB3 and Kif5c. Additionally, like Kif5c, a few EB3 comets are sometimes seen the distal tip of the nascent axon (new Figure 4C) but they do not appear to be enriched compared to the rest of the neuron. In our reworked ms the old Figure 3 is now Figure 4.

Minor comments:

Page 7-line 7: "After pausing the axon extended rapidly and developing a growth cone" should read "After pausing, the axon extended rapidly developing a growth cone".

We have made the suggested modification (top of page 5).

Page 13-line 24 In regard to the sentence "This was surprising, as during neurite formation in vitro, microtubules protrude further into the periphery in areas that will subsequently form a neurite than those that don't (Flynn et al., 2012)". The work by Flynn et al showed that microtubules protrude towards the cell periphery when the dynamism of the actin cytoskeleton allows it. Indeed, this work showed that dynamic actin structures guide and bias microtubules to engorge filopodia that later typically become a nascent neurite, pretty much as the behavior of the actin filaments observed in this study. And the data showed that stabilizing microtubules with taxol does not enhance neurite formation.
The referee is correct, this sentence was misleading. We have removed it (from page 11).

Page 18-line 26 "The mechanisms that may define axon initiation have been studied for several decades but many of the previous works are compromised by studying this process in neurons growing in vitro that lack the complex 3-dimensional architecture and molecular environment of in vivo settings". Over this particular point, and to enrich the discussion, it should be noted that advances have been made towards understanding de the cytoskeleton dynamics of axon formation through the study of neuron polarization in 3D matrices (e.g.

Santos et al., 2020; https://doi.org/10.1016/j.celrep.2020.107907).

We have added this reference and its relevance to 3D environments to our Discussion (page 17).

Referee #3:

Overall, based on well-designed spatial and temporal analyses, the manuscript describes several exciting and novel findings of cellular regulation during initial axon development in vivo and provides new insights into the underlying mechanisms that could be interesting to both cell biologists and neurobiologists. Several points described below might be considered by the authors to broaden the significance of their interesting findings.

1) The title emphasizes on the requirement of microtubules in nascent axon formation, but the data presented have more interesting observations than that related to microtubule alone. Consider the change of the title to provide a balanced view of the data.

The title length is restricted by the Journal and we believe our current title states our most interesting finding, so we do not think it should be changed. But we agree our manuscript has many other interesting findings and we hope they are clear in the main text.

2) The localization of F-actin to nascent axons suggests a critical role, but does not provide a full test of the requirement of this localization. Have the authors done a similar pharmacology experiment similar to microtubule inhibition?

We have not tested the requirement for F-actin or its control. This would indeed be an interesting next step, but we feel it is too large an endeavour to add to our current study. We are careful not to claim any functional requirement for actin in our manuscript, we state F-actin accumulation is an early marker of the location of nascent axon formation and it precedes the enrichment of microtubules in the nascent axon. We briefly propose F-actin is the primary cytoskeletal requirement for nascent axon formation at the end of the Introduction, but we do not claim to have shown this to be the case.

3) In the discussion (p.20-21), the authors suggest nascent axons without microtubules are "less stable .. and eventually tend to retract". Is this statement based on any experimental evidence? If so, it is very important because it indicates a role of microtubules in axon extension after initiation. Including such data would provide a more complete picture of the role of microtubules and strengthen the significance of the paper.

The quantification for the persistence (time to retraction) of the nascent axon in nocodozole neurons was already included in what has now become Figure 5C and main text related to this Figure (on page 13). We have now also included comparisons to control in Figure 5 – this was in response to Reviewer 1 but may also clarify this point.

4) Description of how microtubule tracks were generated in Fig. 3D is missing.

Good point, this is now included in the Materials and Methods on page 25

Dear Prof. Clarke,

Thank you for the submission of your revised manuscript. We have now received the enclosed report from referee 2, who was asked to assess your full response to the referee comments. This referee still has a few minor suggestions that I would like you to incorporate before we can proceed with the official acceptance of your manuscript.

A few other editorial requests also need to be addressed:

- Please correct the conflict of interest subheading to "Disclosure and competing interest statement"

- Fig 1A, 5H, 6L, Appendix Figs S1A, S2A+B, S5A+B, S10E-G callouts are missing. Please add.

- Please upload all movies as individual files, zipped with their legends.

- I attach to this email a related ms file with comments by our data editors. Please address all comments in the final ms file.

- I would like to suggest to add the word "zebrafish" to the title.

EMBO press papers are accompanied online by A) a short (1-2 sentences) summary of the findings and their significance, B) 2-3 bullet points highlighting key results and C) a synopsis image that is exactly 550 pixels wide and 200-600 pixels high (the height is variable). You can either show a model or key data in the synopsis image. Please note that text needs to be readable at the final size. Please send us this information along with the revised manuscript.

I look forward to seeing a new revised version of your manuscript as soon as possible. Please use this link to submit your revision: https://embor.msubmit.net/cgi-bin/main.plex

Best regards,
Esther

Esther Schnapp, PhD
Senior Editor
EMBO reports

Referee #2:

This reviewer appreciates the edits and corrections performed by the authors on the manuscript and acknowledges they greatly improved it.
The data presented is convincing in regard of the following points:
F-actin accumulates at the site of the nascent axon before it is stabilized.
F-actin precedes the enrichment of growing microtubules in the nascent axon.
The F-actin network is the earliest cytoskeletal sign of specification of nascent axon formation in vivo.

However, conceptually, a neurite is considered an axon when the presence of axonal markers or its functionality as such is unambiguously determined. The so called nascent axon or maybe better "prospective axon" could be in its initial stages visualized like a big filopodia that upon engorgement, and posterior stabilization, by microtubules becomes an axon. The data shows that without a microtubule network, these initial neuritic protrusion were less stable (quoting from the text "Compared to normal nascent axon protrusions, those in nocodazole treated cells were shorter, less persistent and less dilated") and had more chances to not being formed at all (success rate 63% vs 24% in control and nocodazole respectively). In this regard, the explanation by the authors that this behaviour could be due to the MT-depleted environment cells are growing in is plausible but unfortunately not demonstrable hence not helping much into clarifying the situation. This reviewer believes that a good compromise would be to strengthen through the manuscript the message that whilst actin acts as a landmark and drives the initial protrusion and extension of the prospective axon, engorgement and stabilization by the microtubule system increases the chances of successful axonal establishment.
All in all, the revised manuscript is very interesting, experimentally strong and well written and, upon addressing the above conceptual points, this reviewer would be happy to endorse this manuscritp for publication by EMBO reports.

P.S.: One comment regarding the authors' response to reviewer #1

"...Thus, is not a suitable approach to test the role of centrosomal activities in axonal fate. In this regard, Stiess et al, Science,

2010 showed that axon elongation (not initiation) is independent of centrosomal microtubules. Contrary to this, the authors stated (page 22): "Combining these findings and others (e.g. (Stiess et al., 2010)) suggests that centrosome proximity is not required for axon initiation but is required for axon growth in vertebrates, at least at early stages."

I think reviewer #1 simply says that Stiess et al show that axon extension functions independently of the centrosome.

Instead of deleting the sentence consider the following as a response to reviewer #1: Make the sentence more clear: "Combining these findings and others (e.g. (Stiess et al., 2010)) suggests that centrosome proximity is not required for axon initiation or axon growth. The role of the centrosome needs to be further tested."

Referee's main concern.
"However, conceptually, a neurite is considered an axon when the presence of axonal markers or its functionality as such is unambiguously determined. The so called nascent axon or maybe better "prospective axon" could be in its initial stages visualized like a big filopodia that upon engorgement, and posterior stabilization, by microtubules becomes an axon. The data shows that without a microtubule network, these initial neuritic protrusion were less stable (quoting from the text "Compared to normal nascent axon protrusions, those in nocodazole treated cells were shorter, less persistent and less dilated") and had more chances to not being formed at all (success rate 63% vs 24% in control and nocodazole respectively). In this regard, the explanation by the authors that this behaviour could be due to the MT-depleted environment cells are growing in is plausible but unfortunately not demonstrable hence not helping much into clarifying the situation. This reviewer believes that a good compromise would be to strengthen through the manuscript the message that whilst actin acts as a landmark and drives the initial protrusion and extension of the prospective axon, engorgement and stabilization by the microtubule system increases the chances of successful axonal establishment."

Our response
We retain the use of the term "nascent axon" in our manuscript, rather than "prospective axon" suggested by the reviewer. We believe prospective implies something about to happen or to be specified in the future, whereas nascent implies something is already coming into existence, and that is the case for the phase of axon initiation that we have investigated.

We are largely in agreement with the referee's comments on what microtubules probably do during the transition from nascent axon to later axon growth and we agree we have not tested these specifically (rather we have demonstrated what they are not required for). To clarify this we have added the words "probably" or "probable" to the various discussions on the likely role of microtubules. We disagree somewhat with the referee's suggestion that the nascent axon is a "big filopodia". Even following nocodazole treatment the nascent axon is clearly distinct from more randomly produced filopodia and it is significantly more dilated and more stable than other protrusions. In this regard microtubules are not required for the initial steps in axon dilation and stability, and we think this is clear from our data and we do not think we have overstated this in our discussions (top page 20).

We have followed the referee's suggestion regarding the Stiess et al 2010 work and amended our sentence on centrosome function (p21).

Prof. Jonathan Clarke
Kings College London
MRC Centre for Developmental Neurobiology
Guys Campus
London SE1 1UL
United Kingdom

Dear Prof. Clarke,

I am very pleased to accept your manuscript for publication in the next available issue of EMBO reports. Thank you for your contribution to our journal.

At the end of this email I include important information about how to proceed. Please ensure that you take the time to read the information and complete and return the necessary forms to allow us to publish your manuscript as quickly as possible.

Given that you as corresponding author are in the UK, you are probably eligible for publication of your article in the open access format in a way that is free of charge for the authors. Please contact either the administration at your institution or our publishers at Wiley (emboreports@wiley.com) for further questions. The general information of Wiley's agreements with the UK can be found here:
https://authorservices.wiley.com/author-resources/Journal-Authors/open-access/affiliation-policies-payments/jisc-agreement.html

As part of the EMBO publication's Transparent Editorial Process, EMBO reports publishes online a Review Process File to accompany accepted manuscripts. As you are aware, this File will be published in conjunction with your paper and will include the referee reports, your point-by-point response and all pertinent correspondence relating to the manuscript.

If you do NOT want this File to be published, please inform the editorial office within 2 days, if you have not done so already, otherwise the File will be published by default [contact: emboreports@embo.org]. If you do opt out, the Review Process File link will point to the following statement: "No Review Process File is available with this article, as the authors have chosen not to make the review process public in this case."

Thank you again for your contribution to EMBO reports and congratulations on a successful publication. Please consider us again in the future for your most exciting work.

Kind regards,
Esther

Esther Schnapp, PhD
Senior Editor
EMBO reports

****

THINGS TO DO NOW:

Please note that you will be contacted by Wiley Author Services to complete licensing and payment information. The required 'Page Charges Authorization Form' is available here: https://www.embopress.org/pb-assets/embo-site/er_apc.pdf - please download and complete the form and return to embopressproduction@wiley.com

You will receive proofs by e-mail approximately 2-3 weeks after all relevant files have been sent to our Production Office; you should return your corrections within 2 days of receiving the proofs.

Please inform us if there is likely to be any difficulty in reaching you at the above address at that time. Failure to meet our deadlines may result in a delay of publication, or publication without your corrections.

All further communications concerning your paper should quote reference number EMBOR-2021-52493V3 and be addressed to emboreports@wiley.com.

Should you be planning a Press Release on your article, please get in contact with emboreports@wiley.com as early as possible, in order to coordinate publication and release dates.

EMBO PRESS

YOU MUST COMPLETE ALL CELLS WITH A PINK BACKGROUND ↓

PLEASE NOTE THAT THIS CHECKLIST WILL BE PUBLISHED ALONGSIDE YOUR PAPER

Corresponding Author Name: Jonathan Clarke

Journal Submitted to: EMBO Reports
Manuscript Number: EMBOR-2021-52493-T

USEFUL LINKS FOR COMPLETING THIS FORM

http://www.antibodypedia.com
http://1degreebio.org
http://www.equator-network.org/reporting-guidelines/improving-bioscience-research-report
http://grants.nih.gov/grants/olaw/olaw.htm
http://www.mrc.ac.uk/Ourresearch/Ethicsresearchguidance/Useofanimals/index.htm
http://ClinicalTrials.gov
http://www.consort-statement.org
http://www.consort-statement.org/checklists/view/32-consort/66-title
http://www.equator-network.org/reporting-guidelines/reporting-recommendations-for-tumo
http://datadryad.org
http://figshare.com
http://www.ncbi.nlm.nih.gov/gap
http://www.ebi.ac.uk/ega
http://biomodels.net/
http://biomodels.net/miriam/
http://jjj.biochem.sun.ac.za
http://oba.od.nih.gov/biosecurity/biosecurity_documents.html
http://www.selectagents.gov/

Reporting Checklist For Life Sciences Articles (Rev. June 2017)

This checklist is used to ensure good reporting standards and to improve the reproducibility of published results. These guidelines are consistent with the Principles and Guidelines for Reporting Preclinical Research issued by the NIH in 2014. Please follow the journal's authorship guidelines in preparing your manuscript.

A- Figures
1. Data
The data shown in figures should satisfy the following conditions:

- the data were obtained and processed according to the field's best practice and are presented to reflect the results of the experiments in an accurate and unbiased manner.
- figure panels include only data points, measurements or observations that can be compared to each other in a scientifically meaningful way.
- graphs include clearly labeled error bars for independent experiments and sample sizes. Unless justified, error bars should not be shown for technical replicates.
- if n< 5, the individual data points from each experiment should be plotted and any statistical test employed should be justified
- Source Data should be included to report the data underlying graphs. Please follow the guidelines set out in the author ship guidelines on Data Presentation.

2. Captions

Each figure caption should contain the following information, for each panel where they are relevant:

- a specification of the experimental system investigated (eg cell line, species name).
- the assay(s) and method(s) used to carry out the reported observations and measurements
- an explicit mention of the biological and chemical entity(ies) that are being measured.
- an explicit mention of the biological and chemical entity(ies) that are altered/varied/perturbed in a controlled manner.

- the exact sample size (n) for each experimental group/condition, given as a number, not a range;
- a description of the sample collection allowing the reader to understand whether the samples represent technical or biological replicates (including how many animals, litters, cultures, etc.).
- a statement of how many times the experiment shown was independently replicated in the laboratory.
- definitions of statistical methods and measures:
  - common tests, such as t-test (please specify whether paired vs. unpaired), simple χ2 tests, Wilcoxon and Mann-Whitney tests, can be unambiguously identified by name only, but more complex techniques should be described in the methods section;
  - are tests one-sided or two-sided?
  - are there adjustments for multiple comparisons?
  - exact statistical test results, e.g., P values = x but not P values < x;
  - definition of 'center values' as median or average;
  - definition of error bars as s.d. or s.e.m.

Any descriptions too long for the figure legend should be included in the methods section and/or with the source data.

In the pink boxes below, please ensure that the answers to the following questions are reported in the manuscript itself. Every question should be answered. If the question is not relevant to your research, please write NA (non applicable). We encourage you to include a specific subsection in the methods section for statistics, reagents, animal models and human subjects.

B- Statistics and general methods

Please fill out these boxes ↓ (Do not worry if you cannot see all your text once you press return)

1.a. How was the sample size chosen to ensure adequate power to detect a pre-specified effect size?	Sample size (number of cells) was determined by the number of live labelled cells imaged at an appropriate timepoint and for which analysis could be performed unambiguously.
1.b. For animal studies, include a statement about sample size estimate even if no statistical methods were used.	N/A
2. Describe inclusion/exclusion criteria if samples or animals were excluded from the analysis. Were the criteria pre-established?	Analysis was performed on cells for which the relevant structure/organelle could be unambiguously identified.
3. Were any steps taken to minimize the effects of subjective bias when allocating animals/samples to treatment (e.g. randomization procedure)? If yes, please describe.	No.
For animal studies, include a statement about randomization even if no randomization was used.	N/A
4.a. Were any steps taken to minimize the effects of subjective bias during group allocation or/and when assessing results (e.g. blinding of the investigator)? If yes please describe.	No
4.b. For animal studies, include a statement about blinding even if no blinding was done	N/A
5. For every figure, are statistical tests justified as appropriate?	Yes.
Do the data meet the assumptions of the tests (e.g., normal distribution)? Describe any methods used to assess it.	Where statistical tests assumed normality, the data followed normal distribution.
Is there an estimate of variation within each group of data?	Yes.

Is the variance similar between the groups that are being statistically compared?	Yes.

C- Reagents

6. To show that antibodies were profiled for use in the system under study (assay and species), provide a citation, catalog number and/or clone number, supplementary information or reference to an antibody validation profile. e.g., Antibodypedia (see link list at top right), 1DegreeBio (see link list at top right).	Chick anti-GFP: Abcam, cat# AB13970<br>Mouse anti-gamma-tubulin: Sigma-Aldrich, cat# T6557<br>Rabbit anti-alpha-tubulin: Abcam, cat# AB233661<br>Alexa goat anti-chick 488: Life Technologies, cat# A11039<br>Alexa goat anti-mouse 568: Life Technologies, cat# A1104<br>Alexa goat anti-rabbit 633: Life Technologies, cat# A21071
7. Identify the source of cell lines and report if they were recently authenticated (e.g., by STR profiling) and tested for mycoplasma contamination.	N/A

* for all hyperlinks, please see the table at the top right of the document

D- Animal Models

8. Report species, strain, gender, age of animals and genetic modification status where applicable. Please detail housing and husbandry conditions and the source of animals.	Zebrafish embryos were obtained from adults maintained under standard conditions (Westerfield 2000) in a 14/10 hour light/dark cycle. Embryos were raised in aquarium water and experiments were performed up to 30 hours post fertilisation. Sex is not yet defined at these stages. The following strains were used - AB/Tuebingen (wildtype strain), Tg(actb2:arl13b-GFP) (transgenic strain) ZFIN ID: ZDB-ALT-100721-1; Tg(actb1:utr-mCherry) (transgenic strain); Sly/lamC1 (mutant strain) ZFIN ID: ZDB-FISH-150901-23200
9. For experiments involving live vertebrates, include a statement of compliance with ethical regulations and identify the committee(s) approving the experiments.	All animal procedures were performed according to the UK Animal (Scientific Procedures) Act 1986 and carried out under Home Office Project Licence number PPL P70880F4C, which was subject to local AWERB Committee review and UK Home Office Approval.
10. We recommend consulting the ARRIVE guidelines (see link list at top right) (PLoS Biol. 8(6), e1000412, 2010) to ensure that other relevant aspects of animal studies are adequately reported. See author guidelines, under 'Reporting Guidelines'. See also: NIH (see link list at top right) and MRC (see link list at top right) recommendations. Please confirm compliance.	Yes.

E- Human Subjects

11. Identify the committee(s) approving the study protocol.	N/A
12. Include a statement confirming that informed consent was obtained from all subjects and that the experiments conformed to the principles set out in the WMA Declaration of Helsinki and the Department of Health and Human Services Belmont Report.	N/A
13. For publication of patient photos, include a statement confirming that consent to publish was obtained.	N/A
14. Report any restrictions on the availability (and/or on the use) of human data or samples.	N/A
15. Report the clinical trial registration number (at ClinicalTrials.gov or equivalent), where applicable.	N/A
16. For phase II and III randomized controlled trials, please refer to the CONSORT flow diagram (see link list at top right) and submit the CONSORT checklist (see link list at top right) with your submission. See author guidelines, under 'Reporting Guidelines'. Please confirm you have submitted this list.	N/A
17. For tumor marker prognostic studies, we recommend that you follow the REMARK reporting guidelines (see link list at top right). See author guidelines, under 'Reporting Guidelines'. Please confirm you have followed these guidelines.	N/A

F- Data Accessibility

18: Provide a "Data Availability" section at the end of the Materials & Methods, listing the accession codes for data generated in this study and deposited in a public database (e.g. RNA-Seq data: Gene Expression Omnibus GSE39462, Proteomics data: PRIDE PXD000208 etc.) Please refer to our author guidelines for 'Data Deposition'.<br><br>Data deposition in a public repository is mandatory for:<br>a. Protein, DNA and RNA sequences<br>b. Macromolecular structures<br>c. Crystallographic data for small molecules<br>d. Functional genomics data<br>e. Proteomics and molecular interactions	N/A
19. Deposition is strongly recommended for any datasets that are central and integral to the study; please consider the journal's data policy. If no structured public repository exists for a given data type, we encourage the provision of datasets in the manuscript as a Supplementary Document (see author guidelines under 'Expanded View' or in unstructured repositories such as Dryad (see link list at top right) or Figshare (see link list at top right).	Data supporting the findings of this study are available within the article, supplementary material and source files.
20. Access to human clinical and genomic datasets should be provided with as few restrictions as possible while respecting ethical obligations to the patients and relevant medical and legal issues. If practically possible and compatible with the individual consent agreement used in the study, such data should be deposited in one of the major public access-controlled repositories such as dbGAP (see link list at top right) or EGA (see link list at top right).	N/A
21. Computational models that are central and integral to a study should be shared without restrictions and provided in a machine-readable form. The relevant accession numbers or links should be provided. When possible, standardized format (SBML, CellML) should be used instead of scripts (e.g. MATLAB). Authors are strongly encouraged to follow the MIRIAM guidelines (see link list at top right) and deposit their model in a public database such as Biomodels (see link list at top right) or JWS Online (see link list at top right). If computer source code is provided with the paper, it should be deposited in a public repository or included in supplementary information.	N/A

G- Dual use research of concern

22. Could your study fall under dual use research restrictions? Please check biosecurity documents (see link list at top right) and list of select agents and toxins (APHIS/CDC) (see link list at top right). According to our biosecurity guidelines, provide a statement only if it could.	No
